# Supplementary material for: Host diversity and behavior determine patterns of interspecies transmission and geographic diffusion of avian influenza A subtypes among North American wild reservoir species
Source: PLoS Pathog. 2022 Apr 13;18(4):e1009973. doi: 10.1371/journal.ppat.1009973 (PMC9037922; doi:10.1371/journal.ppat.1009973)
Supplement: S1 Text — Fig A. Evolutionary parameter estimation for North American avian influenza viruses of wild birds. Estimated parameters include A) time to most recent common ancestor (TMRCA), B) molecular clock rate, and C) effective population size. Parameters are compared across internal gene segments (blue), hemagglutinin gene subtypes (orange), and neuraminidase gene subtypes (purple) as well as between subsampling strategies, phylogenetic diversity-based sample (left, dark grey) and stratified random sample (right, light grey). Median values (black midline) indicated as well as the 95% highest posterior density (whiskers). Fig B. Host species temporal distribution of sampled North American avian influenza virus PB2 gene segment sequences, 2005–2016. Proportions of represented host species are compared between the original, unsampled data set (All), the phylogenetic diversity-based sample (PDA) and the stratified random sample (stratified). Fig C. Geographic region temporal distribution of sampled North American avian influenza virus PB2 gene segment sequences, 2005–2016. Proportions of represented host species are compared between the original, unsampled data set (All), the phylogenetic diversity-based sample (PDA) and the stratified random sample (stratified). Fig D. Host species and geographic region temporal distribution of sampled North American avian influenza virus PB2 gene segment sequences, 2005–2016. Proportions of represented host species are compared between the original, unsampled data set (All), the phylogenetic diversity-based sample (PDA) and the stratified random sample (stratified). Fig E. Heat map of supported viral transition rates among host species across avian influenza virus gene segments and subtypes. Colored cells represent the magnitude of the transition rate from the species in the first column (source) to the species in the second column (sink). White cells were transition rates that were not supported (Bayes factor < 100). Results from both subsamp [file ppat.1009973.s001.pdf]

Full Title: Host diversity and behavior determine patterns of interspecies transmission and geographic diffusion of avian Influenza A subtypes among North American wild reservoir species

Short Title: Ecology and Evolution of Avian Influenza A virus in North American wild birds

AUTHORS: Joseph T. Hicks[1], Kimberly Edwards[2,#], Xueting Qiu[1], Do-Kyun Kim[3], James E. Hixson[3], Scott Krauss[2], Richard J. Webby[2], Robert G. Webster[2], Justin Bahl[1*]

[1]Center for Ecology of Infectious Diseases, Department of Infectious Diseases, College of Veterinary Medicine, Department of Epidemiology and Biostatistics, College of Public Health, Institute of Bioinformatics, University of Georgia, Athens, Georgia, United States; [2]Department of Infectious Disease, St. Jude Children's Research Hospital, Memphis, Tennessee, United States. [3]University of Texas Health Science Center at Houston School of Public Health, Houston, Texas, United States.

# Current Address: School of Public Health, LKS Faculty of Medicine, The University of Hong Kong, Hong Kong, China, HKU-Pasteur Research Pole, School of Public Health, The University of Hong Kong, Hong Kong, China;

*justin.bahl@uga.edu

SUPPLEMENTARY INFORMATION CAPTIONS

Fig A in S1 Text. Evolutionary parameter estimation for North American avian influenza viruses of wild birds. Estimated parameters include A) time to most recent common ancestor (TMRCA), B) molecular clock rate, and C) effective population size. Parameters are compared across internal gene segments (blue), hemagglutinin gene subtypes (orange), and neuraminidase gene subtypes (purple) as well as between subsampling strategies, phylogenetic diversity-based sample (left, dark grey) and stratified random sample (right, light grey). Median values (black midline) indicated as well as the 95% highest posterior density (whiskers).

Fig B in S1 Text. Host species temporal distribution of sampled North American avian influenza virus PB2 gene segment sequences, 2005 – 2016. Proportions of represented host species are compared between the original, unsampled data set (All), the phylogenetic diversity-based sample (PDA) and the stratified random sample (stratified).

Fig C in S1 Text. Geographic region temporal distribution of sampled North American

avian influenza virus PB2 gene segment sequences, 2005 – 2016. Proportions of represented host species are compared between the original, unsampled data set (All), the phylogenetic diversity-based sample (PDA) and the stratified random sample (stratified).

Fig D in S1 Text. Host species and geographic region temporal distribution of sampled North American avian influenza virus PB2 gene segment sequences, 2005 – 2016. Proportions of represented host species are compared between the original, unsampled data set (All), the phylogenetic diversity-based sample (PDA) and the stratified random sample (stratified).

Fig E in S1 Text. Heat map of supported viral transition rates among host species across avian influenza virus gene segments and subtypes. Colored cells represent the magnitude of the transition rate from the species in the first column (source) to the species in the second column (sink). White cells were transition rates that were not supported (Bayes factor < 100). Results from both subsampling strategies (phylogenetic diversity-based sample (PDA) and stratified random sample (stratified)) are presented for comparison.

Fig F in S1 Text. Discrete trait diffusion models of North American avian influenza using a stratified random sample of genetic sequences. Host models (left) are presented for combined internal gene segment (A), hemagglutinin gene subtype (B), and neuraminidase gene subtype (C) models. Source host species on the left of the chord diagrams contribute viral diversity to sink host species on the right. The magnitude of the viral transition rate is proportional to the width of the band, and statistically supported rates darkened. Bands are colored by the host order of the source species (Charadriiformes – red; Anseriformes – blue). Similarly, geographic models (right) are summarized for combined internal gene segment (D), hemagglutinin gene subtype (E), and neuraminidase gene subtype (F) models. Arrow width is proportional to the magnitude of the transition rate, and only statistically supported rates are displayed. (AK – Alaska, AB – Alberta, BC – British Columbia, GT – Guatemala, MW – Midwest, NB – New Brunswick, NE – Northeast, NL – Newfoundland and Labrador, NRP – Northern Rockies and Plains, NS – Nova Scotia, NW – Northwest, OV – Ohio Valley, PE – Prince Edward Island, QC – Quebec, S –

South, SE – Southeast, SON – Sonora, SW – Southwest, W – West). Public domain maps of United States, Canada, and Mexico states, and provinces were accessed from Wikimedia Commons, author Alex Covarrubias (https://commons.wikimedia.org/wiki/File:North_America_second_level_political_division.svg). Public domain map of Guatemala was accessed from *The World Factbook 2021*, Central Intelligence Agency (https://www.cia.gov/the-world-factbook/static/09f14262b8d528e66631646c85e0edc0/north_america_pol.pdf).

Fig G in S1 Text. Heat map of supported viral transition rates among geographic regions across avian influenza virus gene segments and subtypes. Colored cells represent the magnitude of the transition rate from the region in the first column (source) to the region in the second column (sink). White cells were transition rates that were not supported (Bayes factor < 100). Results from both subsampling strategies (phylogenetic diversity-based sample (PDA) and stratified random sample (stratified)) are presented for comparison.

Table A in S1 Text. Demographic characteristics of 303 wild bird surveillance samples with newly sequenced avian influenza isolates, 2003 – 2016.

Table B in S1 Text. Evolutionary parameters of avian influenza virus gene segments collected from North American wild birds between 1970 and 2016. Datasets were sampled so as to maintain the total phylogenetic diversity of the original publicly available sequence sample.

Table C in S1 Text. Host and regional distribution of phylogenetic diversity-based subsample of influenza virus gene segments isolated from North American wild birds.

Table D in S1 Text. Host and regional distribution of stratified subsample of influenza virus gene segments isolated from North American wild birds.

Table E in S1 Text. Cross-tabulation of host and regional distribution of influenza virus gene segments isolated from North American wild birds. Counts and proportions of represented host species by geographic location are compared between the original, unsampled data set (all), the phylogenetic diversity-based sample (PDA) and the stratified random sample (stratified).

Table F in S1 Text. Hemagglutinin and neuraminidase subtype by host order. Counts across all segments and subtypes are compared between the original, unsampled data set (all), the phylogenetic diversity-based sample (PDA) and the stratified random sample (stratified).

Table G in S1 Text. Host species transition rate matrix from combined internal gene model. Median rates and 95% highest posterior density intervals are displayed for both subsampling strategies. Rates colored in blue are statistically supported (Bayes factor > 100). (ABD – American black duck, BUF – bufflehead, BWT – blue-winged teal, CAN – Canada goose, CIN – cinnamon teal, EMP – emperor goose, GAD – gadwall, GWF – greater white-fronted goose, GWG – glaucous-winged gull, GWT – green-winged teal, LAU – laughing gull, MAL – mallard, PIN – northern pintail, RED – redhead, RKN – red knot, RND – ring-necked duck, RUD – ruddy turnstone, SHO – northern shoveler, SND – sanderling, SNO – snow goose, WIG – American wigeon).

Table H in S1 Text. Host species transition rate matrix from combined hemagglutinin subtype model. Median rates and 95% highest posterior density intervals are displayed for both subsampling strategies. Rates colored in blue are statistically supported (Bayes factor > 100). (ABD – American black duck, BUF – bufflehead, BWT – blue-winged teal, CAN – Canada goose, CIN – cinnamon teal, EMP – emperor goose, GAD – gadwall, GWF – greater white-fronted goose, GWG – glaucous-winged gull, GWT – green-winged teal, LAU – laughing gull, MAL – mallard, PIN – northern pintail, RED – redhead, RKN – red knot, RND – ring-necked duck, RUD – ruddy turnstone, SHO – northern shoveler, SND – sanderling, SNO – snow goose, WIG – American wigeon).

Table I in S1 Text. Host species transition rate matrix from combined neuraminidase subtype model. Median rates and 95% highest posterior density intervals are displayed for both subsampling strategies. Rates colored in blue are statistically supported (Bayes factor > 100). (ABD – American black duck, BUF – bufflehead, BWT – blue-winged teal, CAN – Canada goose, CIN – cinnamon teal, EMP – emperor goose, GAD – gadwall, GWF – greater white-fronted goose, GWG – glaucous-winged gull, GWT – green-winged teal, LAU – laughing gull, MAL – mallard, PIN – northern pintail, RED – redhead, RKN – red knot, RND – ring-necked duck, RUD – ruddy turnstone, SHO – northern shoveler, SND – sanderling, SNO – snow goose, WIG – American wigeon).

Table J in S1 Text. Geographic region transition rate matrix from combined internal gene model. Median rates and 95% highest posterior density intervals are displayed for both subsampling strategies. Rates colored in blue are statistically supported (Bayes factor > 100). (AK – Alaska, ALB – Alberta, BCO – British Columbia, GUA – Guatemala, MW – Midwest, NBR – New Brunswick, NE – Northeast, NFL – Newfoundland and Labrador, RP – Northern Rockies and Plains, NSC – Nova Scotia, NW – Northwest, OV – Ohio Valley, PEI – Prince Edward Island, QUE – Quebec, S – South, SE – Southeast, SON – Sonora, SW – Southwest, W – West)

Table K in S1 Text. Geographic region transition rate matrix from combined hemagglutinin subtype model. Median rates and 95% highest posterior density intervals are displayed for both subsampling strategies. Rates colored in blue are statistically supported (Bayes factor > 100). (AK – Alaska, ALB – Alberta, BCO – British Columbia, GUA – Guatemala, MW – Midwest, NBR – New Brunswick, NE – Northeast, NFL – Newfoundland and Labrador, RP – Northern Rockies and Plains, NSC – Nova Scotia, NW – Northwest, OV – Ohio Valley, PEI – Prince Edward Island, QUE – Quebec, S – South, SE – Southeast, SON – Sonora, SW – Southwest, W – West)

Table L in S1 Text. Geographic region transition rate matrix from combined neuraminidase subtype model. Median rates and 95% highest posterior density intervals are displayed for both subsampling strategies. Rates colored in blue are statistically supported (Bayes factor > 100). (AK – Alaska, ALB – Alberta, BCO – British Columbia, GUA – Guatemala, MW – Midwest, NBR – New Brunswick, NE – Northeast, NFL – Newfoundland and Labrador, RP – Northern Rockies and Plains, NSC – Nova Scotia, NW – Northwest, OV – Ohio Valley, PEI – Prince Edward Island, QUE – Quebec, S – South, SE – Southeast, SON – Sonora, SW – Southwest, W – West)

Table M in S1 Text. Names and GenBank accession numbers of 303 newly sequenced AIV nucleotide sequences.

Fig A. Evolutionary parameter estimation for North American avian influenza viruses of wild birds. Estimated parameters include A) time to most recent common ancestor (TMRCA), B) molecular clock rate, and C) effective population size. Parameters are compared across internal gene segments (blue), hemagglutinin gene subtypes (orange), and neuraminidase gene subtypes (purple) as well as between subsampling strategies, phylogenetic diversity-based sample (left, dark grey) and stratified random sample (right, light grey). Median values (black midline) indicated as well as the 95% highest posterior density (whiskers).

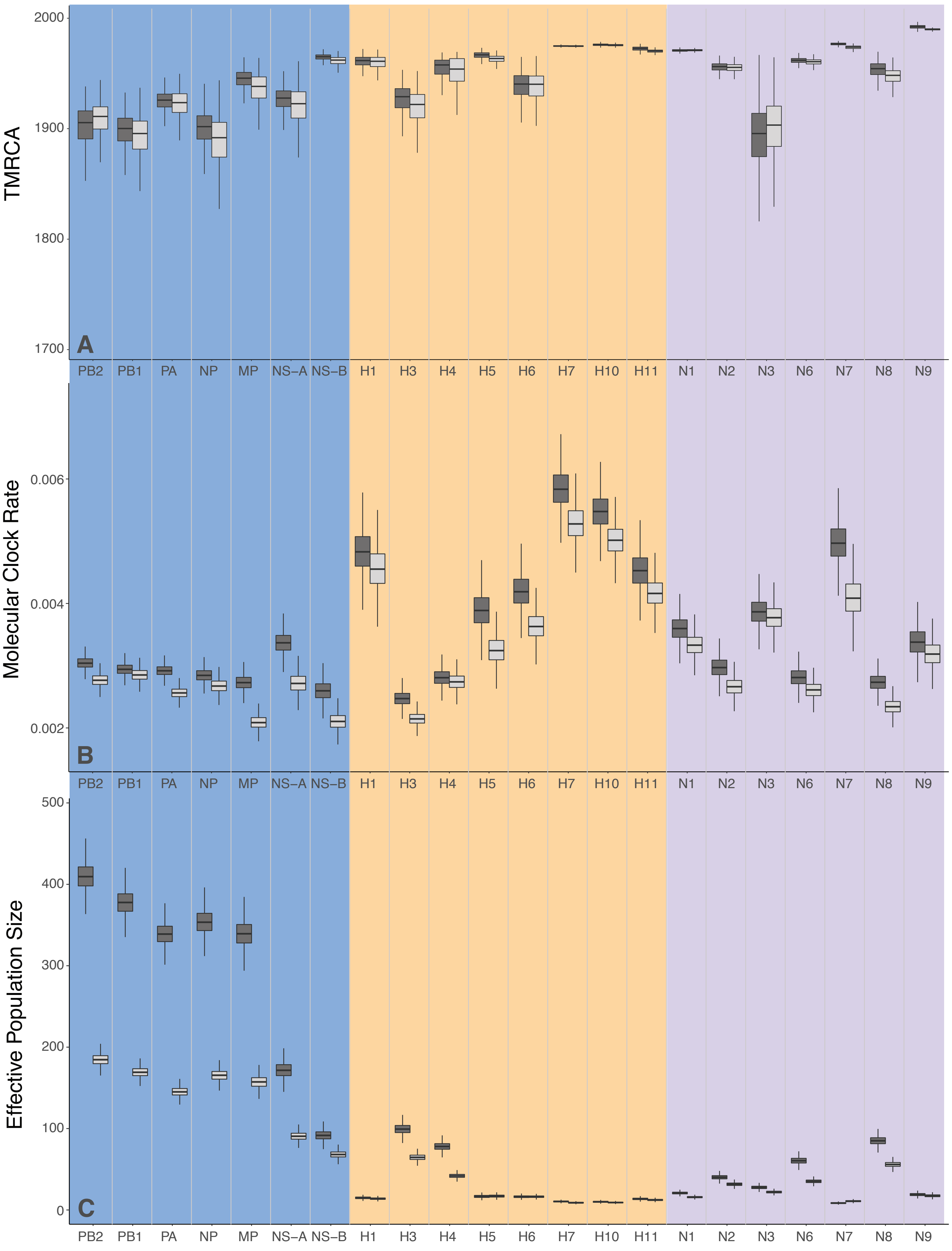
TMRCA
2000
1900
1800
1700
A
PB2
PB1
PA
NP
MP
NS−A
NS−B
H1
H3
H4
H5
H6
H7
H10
H11
N1
N2
N3
N6
N7
N8
N9
Molecular Clock Rate
0.006
0.004
0.002
B
Effective Population Size
500
400
300
200
100
0
C

Fig B. Host species temporal distribution of sampled North American avian influenza virus PB2 gene segment sequences, 2005 – 2016. Proportions of represented host species are compared between the original, unsampled data set (All), the phylogenetic diversity-based sample (PDA) and the stratified random sample (stratified).

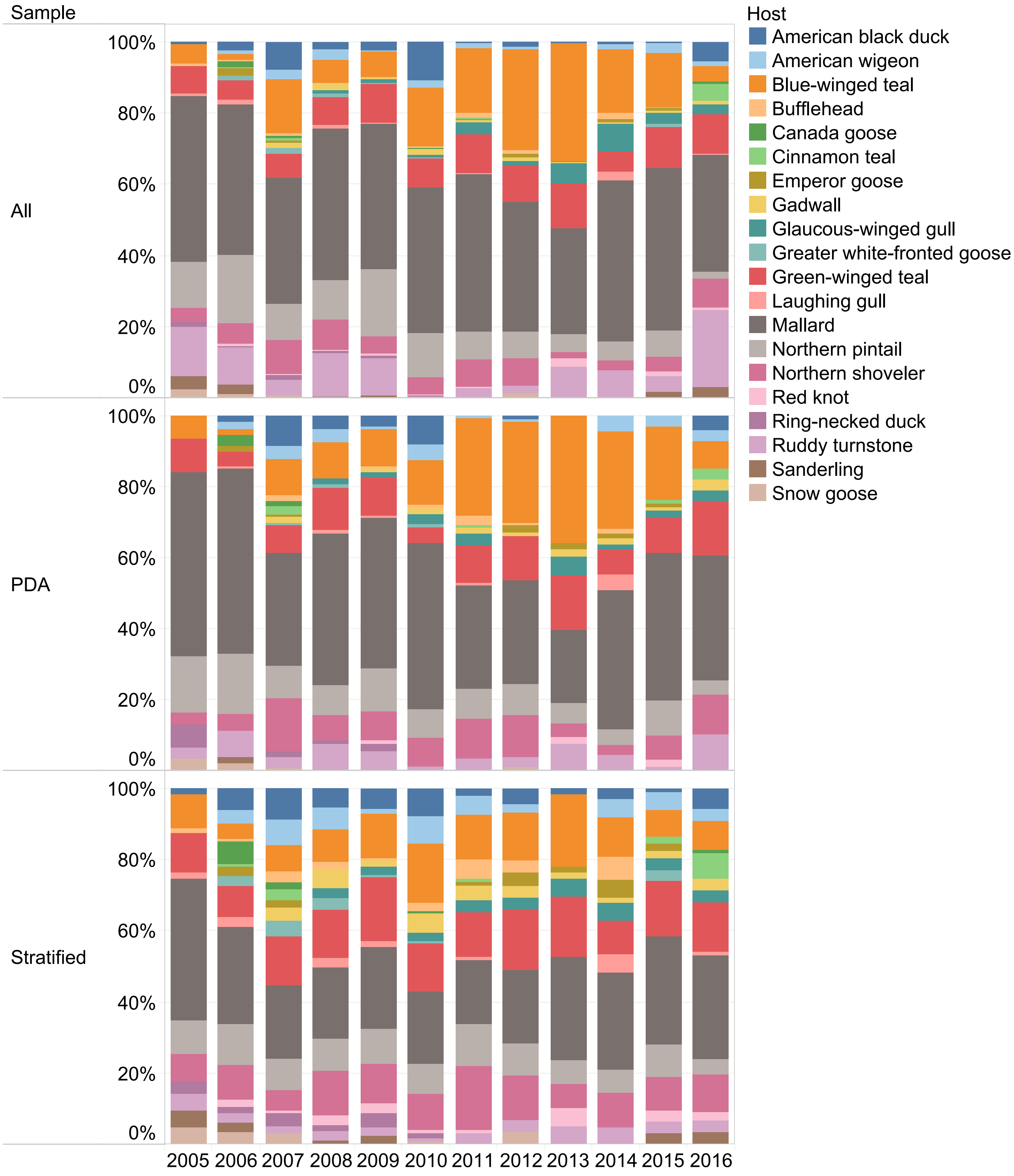

Sample
Host
American black duck
American wigeon
Blue-winged teal
Bufflehead
Canada goose
Cinnamon teal
Emperor goose
Gadwall
Glaucous-winged gull
Greater white-fronted goose
Green-winged teal
Laughing gull
Mallard
Northern pintail
Northern shoveler
Red knot
Ring-necked duck
Ruddy turnstone
Sanderling
Snow goose
All
PDA
Stratified
100%
80%
60%
40%
20%
0%
2005 2006 2007 2008 2009 2010 2011 2012 2013 2014 2015 2016

Fig C. Geographic region temporal distribution of sampled North American avian influenza virus PB2 gene segment sequences, 2005 – 2016. Proportions of represented host species are compared between the original, unsampled data set (All), the phylogenetic diversity-based sample (PDA) and the stratified random sample (stratified).

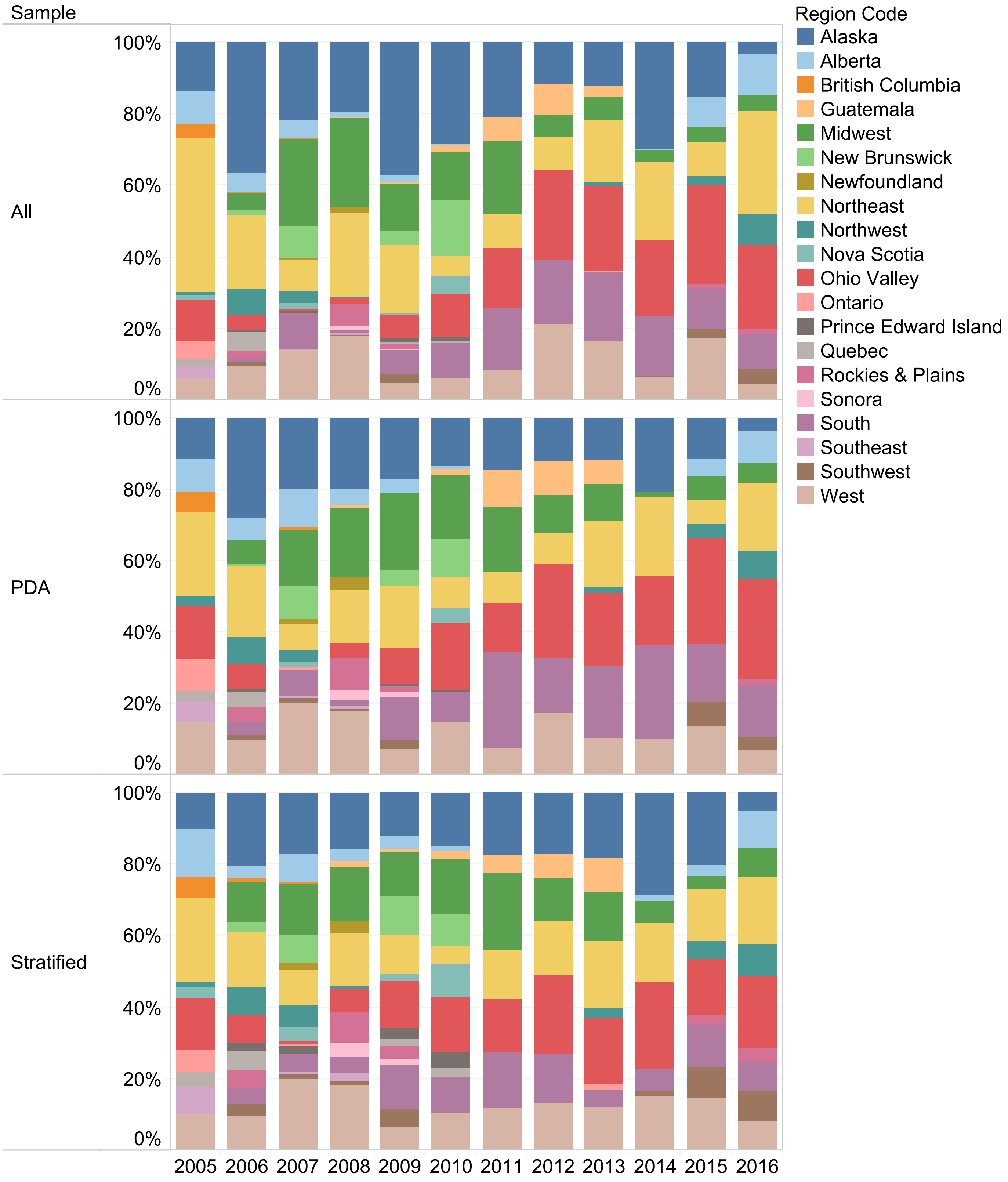
Sample
All
PDA
Stratified
100%
80%
60%
40%
20%
0%
2005 2006 2007 2008 2009 2010 2011 2012 2013 2014 2015 2016
Region Code
Alaska
Alberta
British Columbia
Guatemala
Midwest
New Brunswick
Newfoundland
Northeast
Northwest
Nova Scotia
Ohio Valley
Ontario
Prince Edward Island
Quebec
Rockies & Plains
Sonora
South
Southeast
Southwest
West

Fig D. Host species and geographic region temporal distribution of sampled North American avian influenza virus PB2 gene segment sequences, 2005 – 2016. Proportions of represented host species are compared between the original, unsampled data set (All), the phylogenetic diversity-based sample (PDA) and the stratified random sample (stratified).

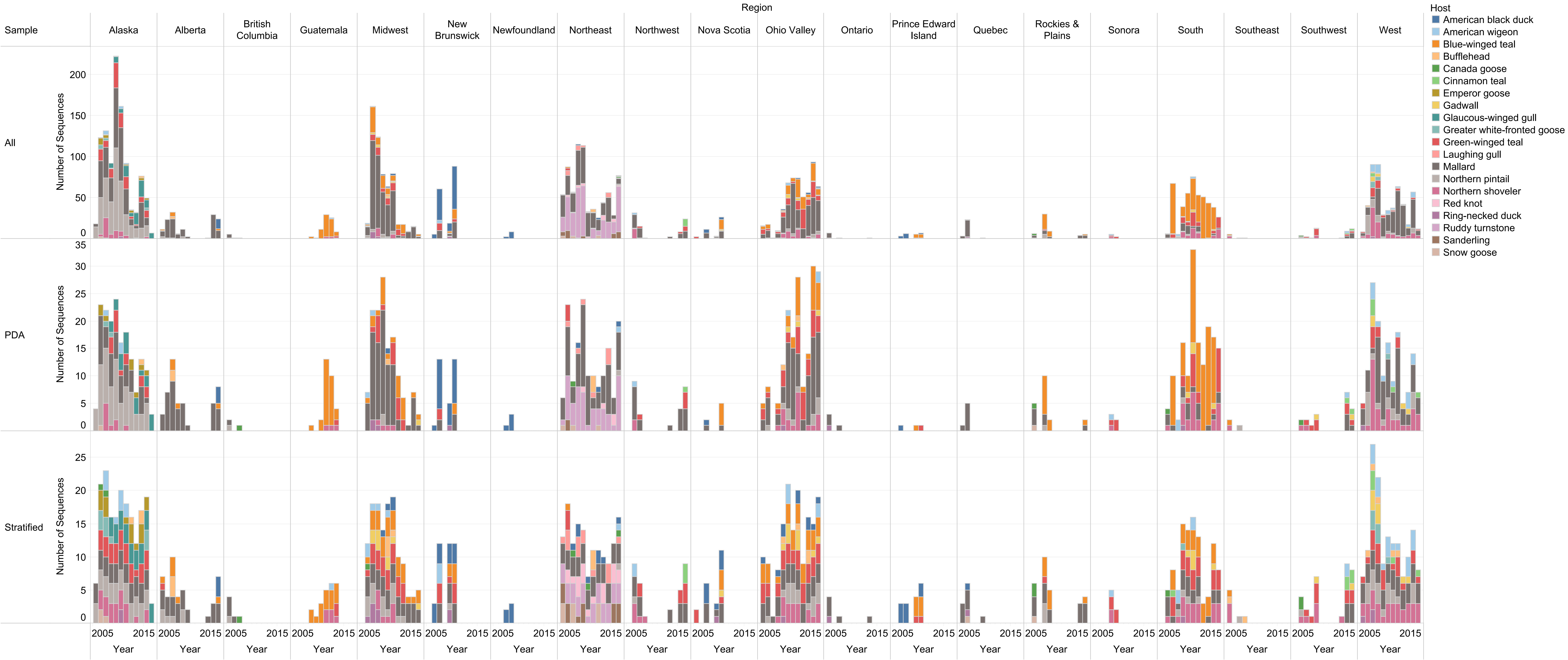

Region
Sample
Alaska
Alberta
British Columbia
Guatemala
Midwest
New Brunswick
Newfoundland
Northeast
Northwest
Nova Scotia
Ohio Valley
Ontario
Prince Edward Island
Quebec
Rockies & Plains
Sonora
South
Southeast
Southwest
West
All
PDA
Stratified
Number of Sequences
Year
2005
2015
Host
American black duck
American wigeon
Blue-winged teal
Bufflehead
Canada goose
Cinnamon teal
Emperor goose
Gadwall
Glaucous-winged gull
Greater white-fronted goose
Green-winged teal
Laughing gull
Mallard
Northern pintail
Northern shoveler
Red knot
Ring-necked duck
Ruddy turnstone
Sanderling
Snow goose

Fig E. Heat map of supported viral transition rates among host species across avian influenza virus gene segments and subtypes. Colored cells represent the magnitude of the transition rate from the species in the first column (source) to the species in the second column (sink). White cells were transition rates that were not supported (Bayes factor < 100). Results from both subsampling strategies (phylogenetic diversity-based sample (PDA) and stratified random sample (stratified)) are presented for comparison.

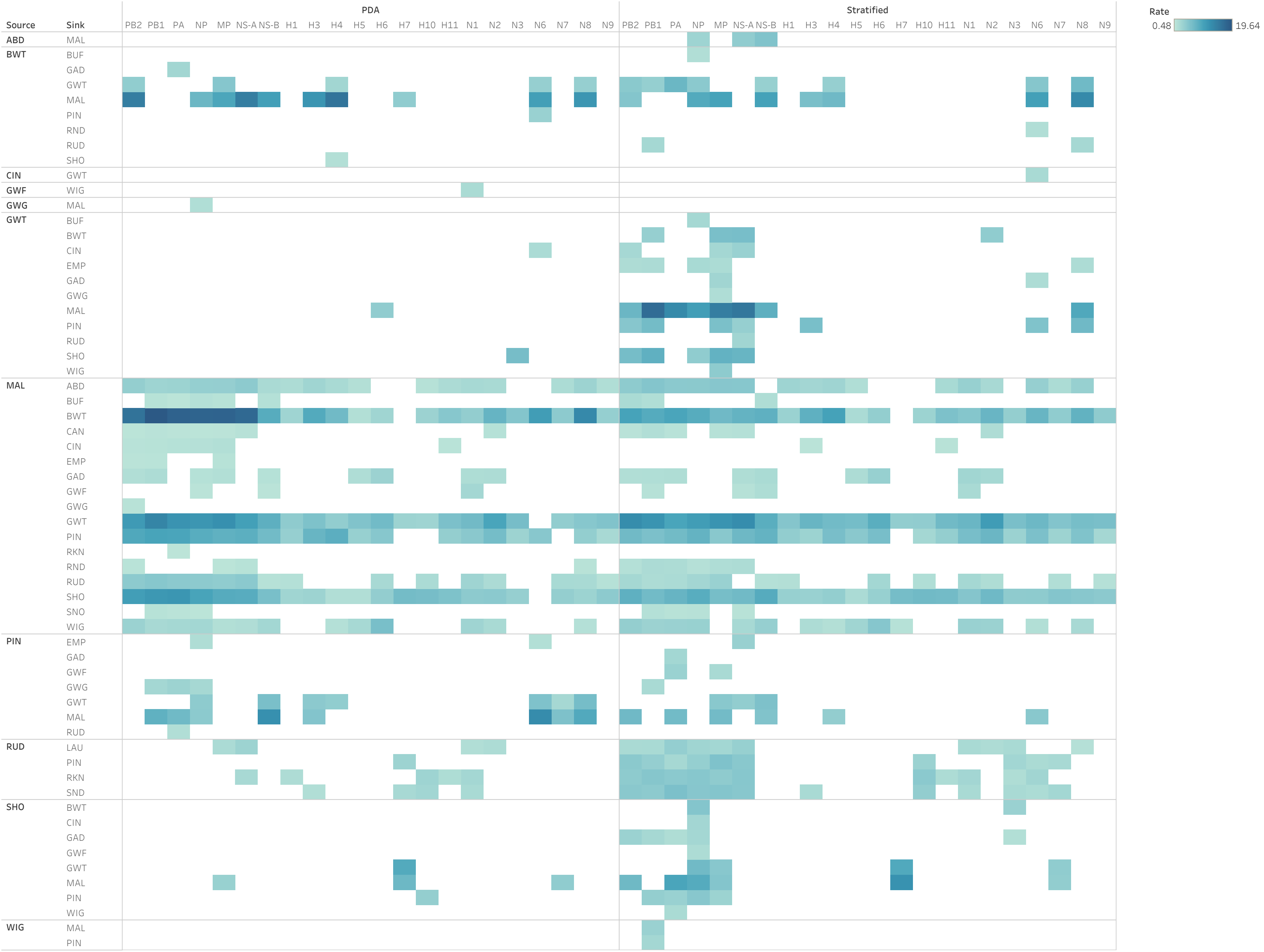
PDA
Stratified
Source
Sink
PB2 PB1 PA NP MP NS-A NS-B H1 H3 H4 H5 H6 H7 H10 H11 N1 N2 N3 N6 N7 N8 N9
PB2 PB1 PA NP MP NS-A NS-B H1 H3 H4 H5 H6 H7 H10 H11 N1 N2 N3 N6 N7 N8 N9
ABD
MAL
BWT
BUF GAD GWT MAL PIN RND RUD SHO
CIN
GWT
GWF
WIG
GWG
MAL
GWT
BUF BWT CIN EMP GAD GWG MAL PIN RUD SHO WIG
MAL
ABD BUF BWT CAN CIN EMP GAD GWF GWG GWT PIN RKN RND RUD SHO SNO WIG
PIN
EMP GAD GWF GWG GWT MAL RUD
RUD
LAU PIN RKN SND
SHO
BWT CIN GAD GWF GWT MAL PIN WIG
WIG
MAL PIN
Rate
0.48
19.64

Fig F. Discrete trait diffusion models of North American avian influenza using a stratified random sample of genetic sequences. Host models (left) are presented for combined internal gene segment (A), hemagglutinin gene subtype (B), and neuraminidase gene subtype (C) models. Source host species on the left of the chord diagrams contribute viral diversity to sink host species on the right. The magnitude of the viral transition rate is proportional to the width of the band, and statistically supported rates darkened. Bands are colored by the host order of the source species (Charadriiformes – red; Anseriformes – blue). Similarly, geographic models (right) are summarized for combined internal gene segment (D), hemagglutinin gene subtype (E), and neuraminidase gene subtype (F) models. Arrow width is proportional to the magnitude of the transition rate, and only statistically supported rates are displayed. (AK – Alaska, AB – Alberta, BC – British Columbia, GT – Guatemala, MW – Midwest, NB – New Brunswick, NE – Northeast, NL – Newfoundland and Labrador, NRP – Northern Rockies and Plains, NS – Nova Scotia, NW – Northwest, OV – Ohio Valley, PE – Prince Edward Island, QC – Quebec, S – South, SE – Southeast, SON – Sonora, SW – Southwest, W – West). Public domain maps of United States, Canada, and Mexico states, and provinces were accessed from Wikimedia Commons, author Alex Covarrubias (https://commons.wikimedia.org/wiki/File:North_America_second_level_political_division.svg). Public domain map of Guatemala was accessed from The World Factbook 2021, Central Intelligence Agency (https://www.cia.gov/the-world-factbook/).

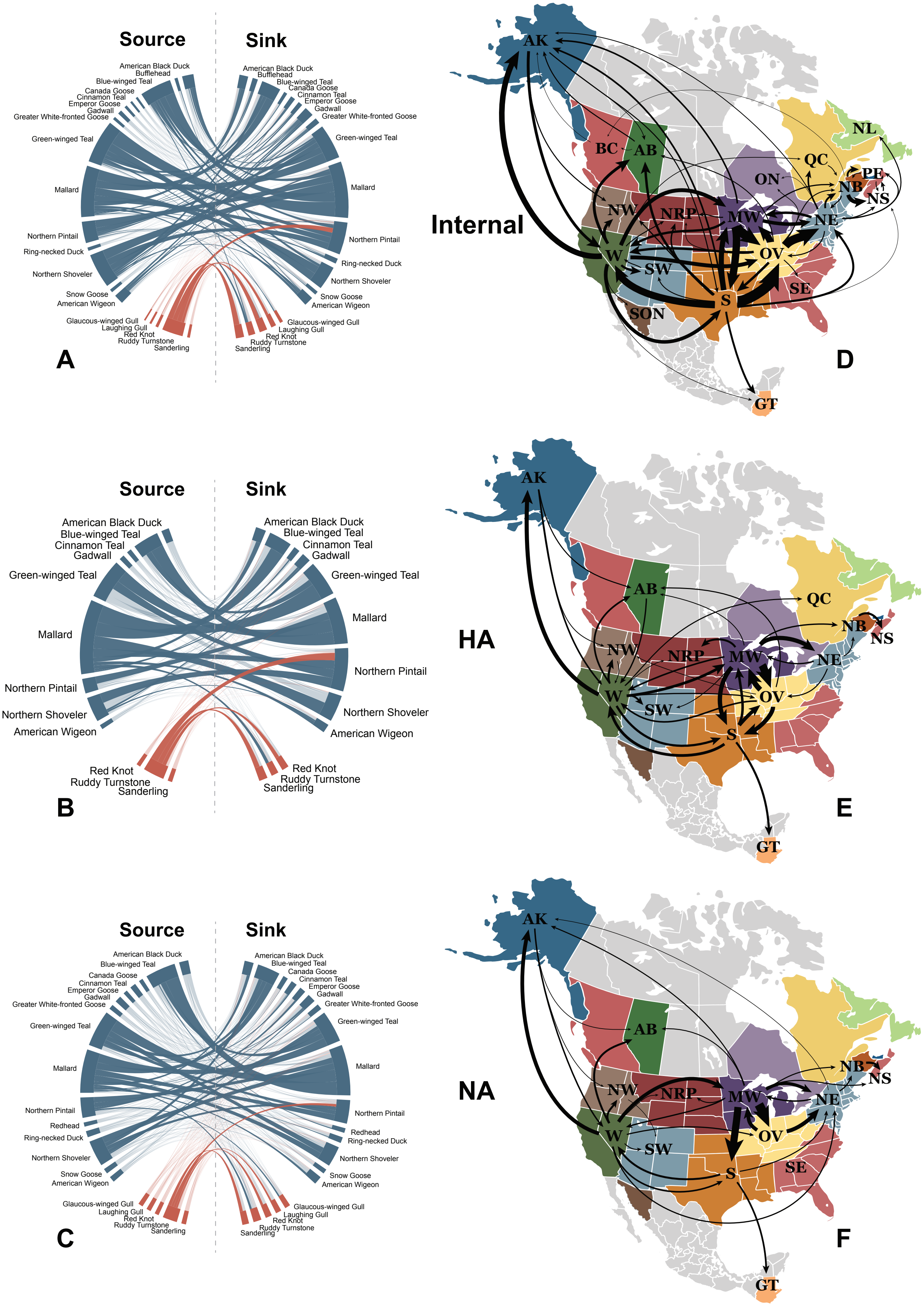

Source
Sink
American Black Duck
Bufflehead
Blue-winged Teal
Canada Goose
Cinnamon Teal
Emperor Goose
Gadwall
Greater White-fronted Goose
Green-winged Teal
Mallard
Northern Pintail
Ring-necked Duck
Northern Shoveler
Snow Goose
American Wigeon
Glaucous-winged Gull
Laughing Gull
Red Knot
Ruddy Turnstone
Sanderling
A
Internal
AK
BC
AB
NL
QC
ON
PE
NB
NS
NW
NRP
MW
NE
W
OV
SW
SE
S
SON
GT
D
Source
Sink
American Black Duck
Blue-winged Teal
Cinnamon Teal
Gadwall
Green-winged Teal
Mallard
Northern Pintail
Northern Shoveler
American Wigeon
Red Knot
Ruddy Turnstone
Sanderling
B
HA
AK
AB
QC
NB
NS
NW
NRP
MW
NE
W
OV
SW
S
GT
E
Source
Sink
American Black Duck
Blue-winged Teal
Canada Goose
Cinnamon Teal
Emperor Goose
Gadwall
Greater White-fronted Goose
Green-winged Teal
Mallard
Northern Pintail
Redhead
Ring-necked Duck
Northern Shoveler
Snow Goose
American Wigeon
Glaucous-winged Gull
Laughing Gull
Red Knot
Ruddy Turnstone
Sanderling
C
NA
AK
AB
NB
NS
NW
NRP
MW
NE
W
OV
SW
SE
S
GT
F

Fig G. Heat map of supported viral transition rates among geographic regions across avian influenza virus gene segments and subtypes. Colored cells represent the magnitude of the transition rate from the region in the first column (source) to the region in the second column (sink). White cells were transition rates that were not supported (Bayes factor < 100). Results from both subsampling strategies (phylogenetic diversity-based sample (PDA) and stratified random sample (stratified)) are presented for comparison.

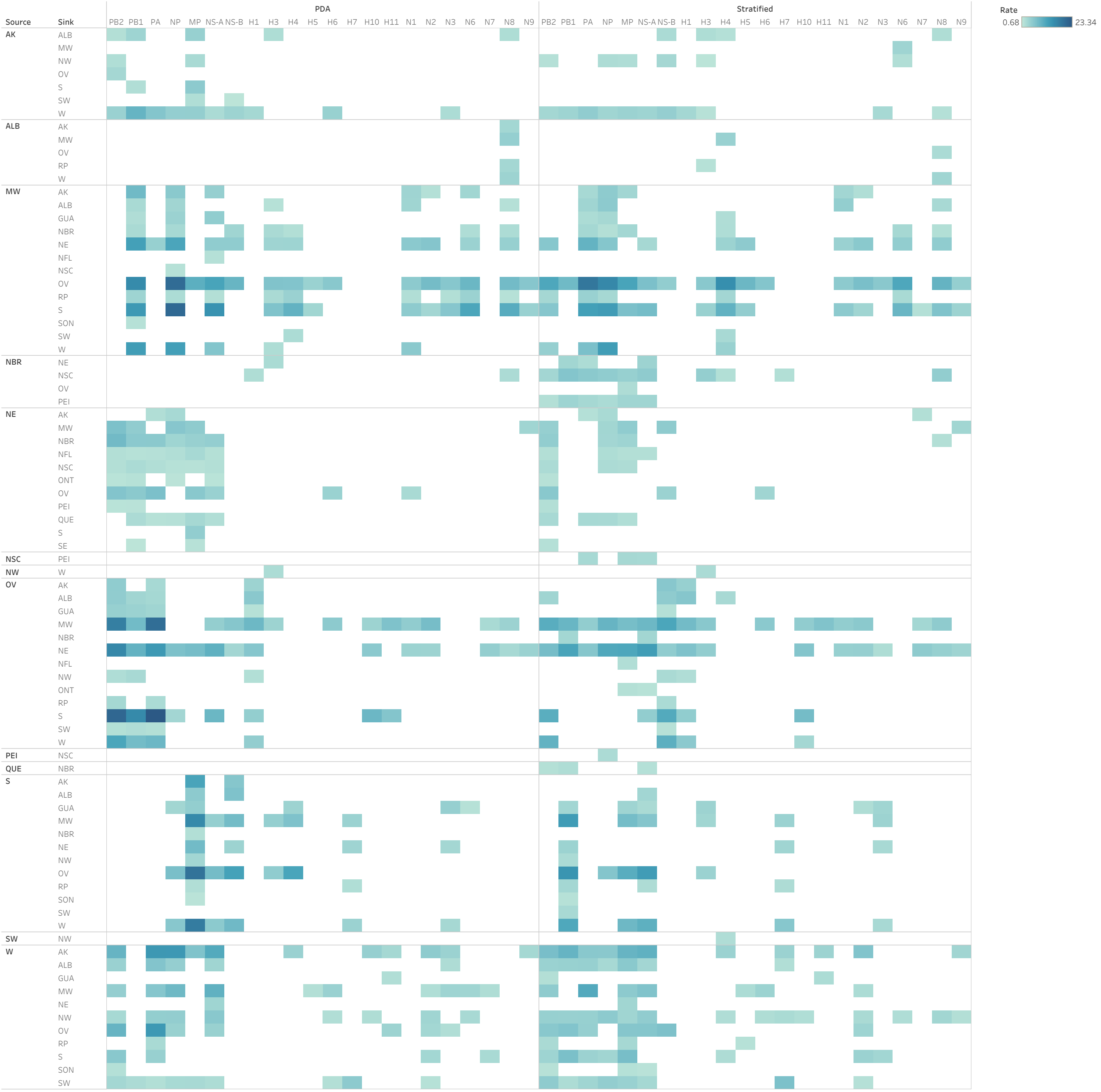
PDA
Stratified
Source
Sink
PB2 PB1 PA NP MP NS-A NS-B H1 H3 H4 H5 H6 H7 H10 H11 N1 N2 N3 N6 N7 N8 N9
PB2 PB1 PA NP MP NS-A NS-B H1 H3 H4 H5 H6 H7 H10 H11 N1 N2 N3 N6 N7 N8 N9
AK: ALB, MW, NW, OV, S, SW, W
ALB: AK, MW, OV, RP, W
MW: AK, ALB, GUA, NBR, NE, NFL, NSC, OV, RP, S, SON, SW, W
NBR: NE, NSC, OV, PEI
NE: AK, MW, NBR, NFL, NSC, ONT, OV, PEI, QUE, S, SE
NSC: PEI
NW: W
OV: AK, ALB, GUA, MW, NBR, NE, NFL, NW, ONT, RP, S, SW, W
PEI: NSC
QUE: NBR
S: AK, ALB, GUA, MW, NBR, NE, NW, OV, RP, SON, SW, W
SW: NW
W: AK, ALB, GUA, MW, NE, NW, OV, RP, S, SON, SW
Rate
0.68
23.34

Table A. Demographic characteristics of 303 wild bird surveillance samples with newly sequenced avian influenza isolates, 2003 – 2016.

**Host Common Name**	n (%)
Ruddy Turnstone (*Arenaria interpres*)	183 (60.4)
Mallard Duck (*Anas platyrhynchos*)	24 (7.9)
Sanderling (*Calidris alba*)	21 (6.9)
Red Knot (*Calidris canutus*)	18 (5.9)
Laughing Gull (*Leucophaeus atricilla*)	11 (3.6)
Semipalmated Sandpiper (*Calidris pusilla*)	11 (3.6)
Gull (*Larus* sp.)	7 (2.3)
American Black Duck (*Anas rubripes*)	6 (2.0)
Spot-billed Duck (*Anas poecilorhyncha*)	4 (1.3)
Duck (*Anas* sp.)	3 (1.0)
Dunlin (*Calidris alpina*)	3 (1.0)
Environment	3 (1.0)
Herring Gull (*Larus smithsonianus*)	3 (1.0)
Shorebird (Charadriiformes)	2 (0.7)
Blue-winged Teal (*Anas discors*)	1 (0.3)
Canada goose (*Branta canadensis*)	1 (0.3)
Greater Black-backed Gull (*Larus marinus*)	1 (0.3)
Kelp Gull (*Larus dominicanus*)	1 (0.3)
**Location**	
Alberta	41 (13.5)
Delaware Bay	262 (86.5)
**Year**	
2003	13 (4.3)
2004	10 (3.3)
2005	17 (5.6)
2006	12 (4.0)
2007	26 (8.6)
2008	15 (5.0)
2009	21 (6.9)
2010	2 (0.7)
2011	10 (3.3)
2012	8 (2.6)
2013	31 (10.2)
2014	31 (10.2)
2015	33 (10.9)
2016	74 (24.4)
**HA[1] Subtype**	
H1	22 (7.3)
H3	28 (9.2)
H4	12 (4.0)
H5	6 (2.0)
H6	9 (3.0)
H7	23 (7.6)
H8	2 (0.7)
H9	13 (4.3)
H10	83 (27.4)
H11	20 (6.6)
H12	57 (18.8)
H13	15 (4.9)
H16	4 (1.3)
APMV Mixed Infection[3]	9 (3.0)
**NA[2] Subtype**	
N1	33 (10.9)
N2	23 (7.6)
N3	33 (10.9)
N4	25 (8.3)
N5	61 (20.1)
N6	28 (9.2)
N7	42 (13.9)
N8	41 (13.5)
N9	8 (2.6)
APMV Mixed Infection[3]	9 (3.0)

[1]Hemagglutinin gene segment

[2]Neuraminidase gene segment

[3]Isolates determined to be co-infections with avian paramyxovirus (excluded from further analysis).

Table B. Evolutionary parameters of avian influenza virus gene segments collected from North American wild birds between 1970 and 2016. Datasets were sampled so as to maintain the total phylogenetic diversity of the original publicly available sequence sample.

Segment	N	TMRCA (95% HPD*)	Effective Population Size (95% HPD*)	Mean Clock Rate (95% HPD*)
**PB2**	1,357	1905.5 (1857.6 – 1930.6)	409.5 (375.9 – 444.3)	3.04E-03 (2.86E-03 – 3.24E-03)
**PB1**	1,401	1900.2 (1867.5 – 1925.3)	377.7 (346.4 – 408.8)	2.94E-03 (2.76E-03 – 3.13E-03)
**PA**	1,363	1925.9 (1907.1 – 1941.7)	338.9 (312.3 – 367.7)	2.92E-03 (2.74E-03 – 3.10E-03)
**NP**	1,328	1901.9 (1863.6 – 1929.5)	353.5 (324.2 – 384.6)	2.84E-03 (2.64E-03 – 3.05E-03)
**MP**	1,308	1945.7 (1927.9 – 1961.4)	339.3 (306.6 – 372.4)	2.73E-03 (2.50E-03 – 2.98E-03)
**NS-A**	1,123	1927.7 (1903.5 – 1945.2)	171.6 (152.0 – 191.1)	3.37E-03 (3.01E-03 – 3.69E-03)
**NS-B**	737	1965.1 (1958.5 – 1969.7)	91.6 (79.6 – 104.6)	2.60E-03 (2.29E-03 – 2.93E-03)
**H1**	280	1961.7 (1949.8 – 1970.1)	14.9 (12.3 – 17.8)	4.83E-03 (4.17E-03 – 5.55E-03)
**H3**	586	1929.0 (1900.0 – 1948.5)	99.3 (87.2 – 112.5)	2.47E-03 (2.23E-03 – 2.71E-03)
**H4**	531	1957.7 (1934.5 – 1967.6)	78.0 (68.3 – 88.2)	2.81E-03 (2.55E-03 – 3.09E-03)
**H5**	224	1966.9 (1960.0 – 1971.9)	16.6 (13.3 – 20.2)	3.89E-03 (3.33E-03 – 4.53E-03)
**H6**	335	1940.4 (1913.0 – 1960.3)	16.3 (13.5 – 19.1)	4.19E-03 (3.64E-03 – 4.76E-03)
**H7**	334	1974.9 (1973.6 – 1975.9)	10.4 (8.8 – 12.0)	5.84E-03 (5.22E-03 – 6.50E-03)
**H10**	280	1976.0 (1974.0 – 1977.8)	10.0 (8.5 – 11.8)	5.48E-03 (4.89E-03 – 6.05E-03)
**H11**	285	1972.7 (1968.3 – 1975.9)	13.6 (11.1 – 16.1)	4.53E-03 (3.98E-03 – 5.16E-03)
**N1**	421	1970.9 (1968.5 – 1972.8)	20.8 (17.9 – 23.9)	3.60E-03 (3.22E-03 – 4.03E-03)
**N2**	472	1956.2 (1946.8 – 1963.2)	40.2 (34.5 – 45.8)	2.97E-03 (2.65E-03 – 3.33E-03)
**N3**	399	1895.6 (1830.8 – 1944.7)	27.7 (23.7 – 31.8)	3.87E-03 (3.45E-03 – 4.33E-03)
**N6**	501	1961.9 (1956.2 – 1966.4)	60.5 (52.8 – 70.0)	2.81E-03 (2.53E-03 – 3.13E-03)
**N7**	262	1976.8 (1974.3 – 1978.7)	8.5 (7.0 – 9.9)	4.97E-03 (4.35E-03 – 5.63E-03)
**N8**	599	1954.4 (1938.4 – 1966.7)	85.0 (74.8 – 96.0)	2.73E-03 (2.47E-03 – 3.02E-03)
**N9**	237	1992.3 (1988.4 – 1995.3)	18.9 (15.5 – 22.6)	3.38E-03 (2.92E-03 – 3.85E-03)

*95% HPD: 95% Highest posterior density

Table C. Host and regional distribution of phylogenetic diversity-based subsample of influenza virus gene segments isolated from North American wild birds.

Region	PB2		PB1		PA		NP		MP		NS-A		NS-B	
Canada	n	%	n	%	n	%	n	%	n	%	n	%	n	%
Alberta	50	3.7	44	3.1	47	3.4	38	2.9	50	3.8	32	2.8	37	5
British Columbia	3	0.2	1	0.1	4	0.3	2	0.2	2	0.2	2	0.2	1	0.1
New Brunswick	33	2.4	35	2.5	27	2	29	2.2	30	2.3	25	2.2	17	2.3
Newfoundland and Labrador	6	0.4	5	0.4	6	0.4	7	0.5	15	1.1	20	1.8	9	1.2
Nova Scotia	7	0.5	8	0.6	6	0.4	10	0.8	8	0.6	8	0.7	7	0.9
Ontario	4	0.3	5	0.4	2	0.1	3	0.2	3	0.2	3	0.3	1	0.1
Prince Edward Island	3	0.2	3	0.2	2	0.1	2	0.2	4	0.3	2	0.2	2	0.3
Quebec	6	0.4	9	0.6	7	0.5	7	0.5	12	0.9	7	0.6	2	0.3
United States														
Alaska	197	14.5	195	13.9	199	14.6	171	12.9	175	13.4	145	12.9	185	25.1
Midwest	156	11.5	161	11.5	154	11.3	155	11.7	150	11.5	131	11.7	86	11.7
Northeast	166	12.2	168	12	160	11.7	156	11.7	156	11.9	165	14.7	71	9.6
Northwest	28	2.1	25	1.8	33	2.4	28	2.1	28	2.1	28	2.5	14	1.9
Ohio Valley	185	13.6	199	14.2	197	14.5	186	14	197	15.1	147	13.1	108	14.7
Northern Rockies and Plains	19	1.4	18	1.3	24	1.8	15	1.1	13	1	10	0.9	5	0.7
South	155	11.4	155	11.1	169	12.4	156	11.7	150	11.5	143	12.7	63	8.5
Southeast	4	0.3	5	0.4	6	0.4	6	0.5	7	0.5	4	0.4	4	0.5
Southwest	19	1.4	14	1	15	1.1	18	1.4	19	1.5	12	1.1	2	0.3
West	152	11.2	156	11.1	157	11.5	153	11.5	147	11.2	112	10	89	12.1
Mexico														
Sonora	5	0.4	6	0.4	4	0.3	2	0.2	5	0.4	4	0.4	2	0.3
Guatemala	30	2.2	26	1.9	28	2.1	30	2.3	27	2.1	29	2.6	4	0.5
Rare	4	0.3	6	0.4	5	0.4	5	0.4	3	0.2	6	0.5	1	0.1
Before 2006	125	9.2	157	11.2	111	8.1	149	11.2	107	8.2	88	7.8	27	3.7
**Host**	**PB2**		**PB1**		**PA**		**NP**		**MP**		**NS-A**		**NS-B**	
American black duck (*Anas rubripes*)	35	2.6	31	2.2	30	2.2	32	2.4	38	2.9	36	3.2	24	3.3
Bufflehead (*Bucephala albeola*)	9	0.7	8	0.6	7	0.5	9	0.7	8	0.6	4	0.4	13	1.8
Blue winged teal (*Spatula discors*)	184	13.6	186	13.3	183	13.4	187	14.1	195	14.9	179	15.9	94	12.8
Canada goose (*Branta canadensis*)	5	0.4	6	0.4	5	0.4	4	0.3	5	0.4	6	0.5	2	0.3
Cinnamon teal (*Spatula cyanoptera*)	8	0.6	8	0.6	10	0.7	10	0.8	11	0.8	9	0.8	0	0
Emperor goose (*Anser canagicus*)	8	0.6	7	0.5	5	0.4	6	0.5	10	0.8	3	0.3	4	0.5
Gadwall (*Mareca strepera*)	14	1	14	1	12	0.9	8	0.6	12	0.9	11	1	7	0.9
Greater white fronted goose (*Anser albifrons*)	3	0.2	3	0.2	7	0.5	6	0.5	3	0.2	4	0.4	6	0.8
Glaucous winged gull (*Larus glaucescens*)	20	1.5	20	1.4	23	1.7	21	1.6	5	0.4	23	2	1	0.1
Green winged teal (*Anas crecca*)	112	8.3	126	9	119	8.7	112	8.4	128	9.8	86	7.7	82	11.1
Laughing gull (*Larus atricilla*)	7	0.5	3	0.2	3	0.2	2	0.2	3	0.2	8	0.7	0	0
Mallard (*Anas platyrhynchos*)	445	32.8	447	31.9	460	33.7	427	32.2	455	34.8	369	32.9	271	36.8
Northern pintail (*Anas acuta*)	108	8	105	7.5	112	8.2	91	6.9	78	6	69	6.1	82	11.1
Red knot (*Calidris canutus*)	4	0.3	5	0.4	5	0.4	7	0.5	6	0.5	8	0.7	0	0
Ring necked duck (*Aythya collaris*)	8	0.6	5	0.4	2	0.1	6	0.5	5	0.4	6	0.5	4	0.5
Ruddy Turnstone (*Arenaria interpres*)	54	4	53	3.8	55	4	46	3.5	50	3.8	63	5.6	12	1.6
Northern shoveler (*Spatula clypeata*)	100	7.4	106	7.6	111	8.1	99	7.5	95	7.3	72	6.4	50	6.8
Sanderling (*Calidris alba*)	2	0.1	3	0.2	2	0.1	1	0.1	4	0.3	2	0.2	1	0.1
Snow goose (*Anser caerulescens*)	5	0.4	9	0.6	7	0.5	6	0.5	5	0.4	4	0.4	4	0.5
American wigeon (*Mareca americana*)	28	2.1	20	1.4	22	1.6	24	1.8	12	0.9	13	1.2	19	2.6
Rare	73	5.4	79	5.6	72	5.3	75	5.6	73	5.6	60	5.3	34	4.6
Before 2006	125	9.2	157	11.2	111	8.1	149	11.2	107	8.2	88	7.8	27	3.7

Region	H1		H3		H4		H5		H6		H7		H10		H11	
Canada	n	%	n	%	n	%	n	%	n	%	n	%	n	%	n	%
Alberta	30	10.7	23	3.9	16	3	1	0.4	2	0.6	4	1.2	6	2.1	1	0.4
New Brunswick	9	3.2	21	3.6	14	2.6	2	0.9	0	0	5	1.5	5	1.8	5	1.8
Nova Scotia	3	1.1	6	1	3	0.6	0	0	4	1.2	1	0.3	1	0.4	1	0.4
Quebec	3	1.1	4	0.7	4	0.8	3	1.3	0	0	0	0	1	0.4	2	0.7
United States																
Alaska	37	13.2	179	30.5	109	20.5	17	7.6	22	6.6	37	11.1	41	14.6	14	4.9
Midwest	37	13.2	101	17.2	82	15.4	24	10.7	58	17.3	22	6.6	22	7.9	39	13.7
Northeast	58	20.7	35	6	28	5.3	31	13.8	53	15.8	53	15.9	87	31.1	57	20
Northwest	6	2.1	15	2.6	8	1.5	9	4	6	1.8	5	1.5	4	1.4	5	1.8
Ohio Valley	34	12.1	58	9.9	76	14.3	38	17	55	16.4	35	10.5	35	12.5	53	18.6
Northern Rockies and Plains	3	1.1	10	1.7	15	2.8	5	2.2	4	1.2	9	2.7	1	0.4	0	0
South	13	4.6	52	8.9	81	15.3	15	6.7	9	2.7	78	23.4	30	10.7	22	7.7
Southwest	0	0	4	0.7	10	1.9	3	1.3	3	0.9	26	7.8	0	0	2	0.7
West	25	8.9	24	4.1	44	8.3	46	20.5	94	28.1	33	9.9	31	11.1	57	20
Guatemala	5	1.8	17	2.9	11	2.1	6	2.7	0	0	4	1.2	0	0	6	2.1
Rare	4	1.4	11	1.9	8	1.5	14	6.3	5	1.5	6	1.8	2	0.7	6	2.1
Before 2006	13	4.6	26	4.4	22	4.1	10	4.5	20	6	16	4.8	14	5	15	5.3
**Host**	**H1**		**H3**		**H4**		**H5**		**H6**		**H7**		**H10**		**H11**	
American black duck (*Anas rubripes*)	9	3.2	21	3.6	13	2.4	4	1.8	2	0.6	4	1.2	5	1.8	7	2.5
Blue winged teal (*Spatula discors*)	12	4.3	122	20.8	160	30.1	7	3.1	13	3.9	40	12	18	6.4	20	7
Cinnamon teal (*Spatula cyanoptera*)	0	0	3	0.5	3	0.6	2	0.9	0	0	6	1.8	1	0.4	2	0.7
Gadwall (*Mareca strepera*)	0	0	4	0.7	1	0.2	5	2.2	15	4.5	2	0.6	1	0.4	3	1.1
Green winged teal (*Anas crecca*)	22	7.9	56	9.6	38	7.2	20	8.9	39	11.6	45	13.5	17	6.1	25	8.8
Mallard (*Anas platyrhynchos*)	140	50	198	33.8	185	34.8	118	52.7	104	31	94	28.1	90	32.1	120	42.1
Northern pintail (*Anas acuta*)	18	6.4	95	16.2	56	10.5	16	7.1	36	10.7	21	6.3	15	5.4	17	6
Red knot (*Calidris canutus*)	3	1.1	0	0	1	0.2	0	0	0	0	1	0.3	5	1.8	4	1.4
Ruddy Turnstone (*Arenaria interpres*)	19	6.8	9	1.5	6	1.1	6	2.7	24	7.2	23	6.9	51	18.2	26	9.1
Northern shoveler (*Spatula clypeata*)	12	4.3	22	3.8	16	3	10	4.5	20	6	59	17.7	32	11.4	20	7
Sanderling (*Calidris alba*)	3	1.1	2	0.3	0	0	0	0	0	0	3	0.9	4	1.4	4	1.4
American wigeon (*Mareca americana*)	0	0	7	1.2	6	1.1	8	3.6	31	9.3	3	0.9	2	0.7	0	0
Rare	29	10.4	21	3.6	24	4.5	18	8	31	9.3	17	5.1	25	8.9	22	7.7
Before 2006	13	4.6	26	4.4	22	4.1	10	4.5	20	6	16	4.8	14	5	15	5.3

Region	N1		N2		N3		N6		N7		N8		N9	
Canada	n	%	n	%	n	%	n	%	n	%	n	%	n	%
Alberta	20	4.8	3	0.6	9	2.3	15	3	8	3.1	24	4	4	1.7
New Brunswick	5	1.2	4	0.8	2	0.5	16	3.2	10	3.8	25	4.2	5	2.1
Nova Scotia	4	1	0	0	1	0.3	3	0.6	0	0	9	1.5	1	0.4
United States														
Alaska	43	10.2	43	9.1	73	18.3	130	25.9	39	14.9	147	24.5	24	10.1
Midwest	67	15.9	96	20.3	38	9.5	71	14.2	13	5	105	17.5	48	20.3
Northeast	81	19.2	63	13.3	44	11	43	8.6	66	25.2	50	8.3	36	15.2
Northwest	9	2.1	12	2.5	3	0.8	10	2	3	1.1	11	1.8	5	2.1
Ohio Valley	63	15	78	16.5	46	11.5	64	12.8	31	11.8	66	11	37	15.6
Northern Rockies and Plains	6	1.4	2	0.4	5	1.3	15	3	5	1.9	13	2.2	1	0.4
South	28	6.7	33	7	59	14.8	60	12	38	14.5	76	12.7	22	9.3
Southeast	1	0.2	2	0.4	1	0.3	1	0.2	0	0	1	0.2	0	0
Southwest	2	0.5	3	0.6	17	4.3	2	0.4	4	1.5	0	0	0	0
West	65	15.4	65	13.8	55	13.8	39	7.8	31	11.8	27	4.5	44	18.6
Guatemala	0	0	18	3.8	24	6	2	0.4	0	0	7	1.2	3	1.3
Rare	5	1.2	25	5.3	15	3.8	9	1.8	3	1.1	10	1.7	3	1.3
Before 2006	22	5.2	25	5.3	7	1.8	21	4.2	11	4.2	28	4.7	4	1.7
**Host**	**N1**		**N2**		**N3**		**N6**		**N7**		**N8**		**N9**	
American black duck (*Anas rubripes*)	12	2.9	8	1.7	4	1	18	3.6	6	2.3	22	3.7	9	3.8
Blue winged teal (*Spatula discors*)	23	5.5	50	10.6	50	12.5	117	23.4	29	11.1	128	21.4	17	7.2
Canada goose (*Branta canadensis*)	2	0.5	6	1.3	1	0.3	1	0.2	0	0	0	0	0	0
Cinnamon teal (*Spatula cyanoptera*)	0	0	0	0	5	1.3	3	0.6	2	0.8	3	0.5	2	0.8
Emperor goose (*Anser canagicus*)	1	0.2	4	0.8	0	0	4	0.8	1	0.4	1	0.2	1	0.4
Gadwall (*Mareca strepera*)	9	2.1	8	1.7	3	0.8	0	0	2	0.8	2	0.3	1	0.4
Greater white fronted goose (*Anser albifrons*)	13	3.1	2	0.4	0	0	0	0	2	0.8	0	0	0	0
Glaucous winged gull (*Larus glaucescens*)	1	0.2	10	2.1	38	9.5	2	0.4	0	0	1	0.2	1	0.4
Green winged teal (*Anas crecca*)	32	7.6	44	9.3	46	11.5	41	8.2	20	7.6	58	9.7	25	10.5
Laughing gull (*Larus atricilla*)	2	0.5	2	0.4	1	0.3	1	0.2	0	0	1	0.2	3	1.3
Mallard (*Anas platyrhynchos*)	186	44.2	180	38.1	136	34.1	167	33.3	83	31.7	197	32.9	112	47.3
Northern pintail (*Anas acuta*)	22	5.2	38	8.1	21	5.3	75	15	17	6.5	79	13.2	10	4.2
Redhead (*Aythya americana*)	1	0.2	2	0.4	0	0	2	0.4	2	0.8	1	0.2	0	0
Red knot (*Calidris canutus*)	3	0.7	1	0.2	1	0.3	1	0.2	3	1.1	0	0	0	0
Ring necked duck (*Aythya collaris*)	2	0.5	0	0	0	0	0	0	1	0.4	4	0.7	1	0.4
Ruddy Turnstone (*Arenaria interpres*)	32	7.6	22	4.7	15	3.8	13	2.6	46	17.6	14	2.3	14	5.9
Northern shoveler (*Spatula clypeata*)	21	5	30	6.4	44	11	18	3.6	26	9.9	22	3.7	19	8
Sanderling (*Calidris alba*)	4	1	1	0.2	0	0	0	0	1	0.4	1	0.2	0	0
Snow goose (*Anser caerulescens*)	4	1	1	0.2	4	1	0	0	0	0	1	0.2	1	0.4
American wigeon (*Mareca americana*)	15	3.6	11	2.3	4	1	3	0.6	2	0.8	8	1.3	0	0
Rare	14	3.3	27	5.7	19	4.8	14	2.8	8	3.1	28	4.7	17	7.2
Before 2006	22	5.2	25	5.3	7	1.8	21	4.2	11	4.2	28	4.7	4	1.7

Table D. Host and regional distribution of stratified subsample of influenza virus gene segments isolated from North American wild birds.

Region	PB2		PB1		PA		NP		MP		NS-A		NS-B	
Canada	n	%	n	%	n	%	n	%	n	%	n	%	n	%
Alberta	50	3.7	53	3.8	53	3.9	53	4	51	3.9	39	3.5	33	4.5
British C	6	0.4	7	0.5	6	0.4	6	0.5	7	0.5	3	0.3	3	0.4
New Bru	42	3.1	41	2.9	41	3	40	3	37	2.8	34	3	22	3
Newfoun	7	0.5	7	0.5	7	0.5	9	0.7	23	1.8	16	1.4	9	1.2
Nova Sco	23	1.7	25	1.8	24	1.8	25	1.9	23	1.8	19	1.7	13	1.8
Ontario	6	0.4	6	0.4	6	0.4	5	0.4	6	0.5	5	0.5	2	0.3
Prince Ed	16	1.2	18	1.3	16	1.2	16	1.2	15	1.2	14	1.3	2	0.3
Quebec	15	1.1	16	1.1	14	1	13	1	11	0.8	11	1	6	0.8
United States														
Alaska	204	15	202	14.4	199	14.6	196	14.8	180	13.8	156	13.9	128	17.4
Midwest	148	10.9	153	10.9	151	11.1	140	10.5	138	10.6	115	10.2	82	11.1
Northeast	173	12.8	173	12.4	174	12.8	171	12.9	160	12.2	153	13.6	64	8.7
Northwes	36	2.7	62	4.4	42	3.1	36	2.7	35	2.7	34	3	21	2.9
Ohio Val	165	12.2	166	11.9	165	12.1	163	12.3	165	12.6	140	12.5	91	12.4
Northern	28	2.1	28	2	27	2	23	1.7	24	1.8	20	1.8	8	1.1
South	105	7.7	105	7.5	106	7.8	102	7.7	102	7.8	94	8.4	50	6.8
Southeast	9	0.7	11	0.8	10	0.7	10	0.8	10	0.8	5	0.5	5	0.7
Southwes	32	2.4	34	2.4	29	2.1	31	2.3	30	2.3	24	2.1	6	0.8
West	158	11.6	159	11.4	159	11.7	156	11.8	158	12.1	114	10.2	93	12.6
Mexico														
Sonora	7	0.5	7	0.5	8	0.6	7	0.5	8	0.6	6	0.5	2	0.3
Guatemala	23	1.7	24	1.7	23	1.7	22	1.7	21	1.6	18	1.6	5	0.7
Rare	11	0.8	11	0.8	10	0.7	11	0.8	11	0.8	10	0.9	1	0.1
Before 2006	93	6.9	93	6.6	93	6.8	93	7	93	7.1	93	8.3	91	12.4
**Host**	**PB2**		**PB1**		**PA**		**NP**		**MP**		**NS-A**		**NS-B**	
American bla	58	4.3	60	4.3	57	4.2	58	4.4	64	4.9	52	4.6	25	3.4
Bufflehead (*I*	24	1.8	23	1.6	23	1.7	22	1.7	22	1.7	8	0.7	15	2
Blue winged	124	9.1	130	9.3	129	9.5	126	9.5	126	9.6	116	10.3	78	10.6
Canada goos	12	0.9	17	1.2	10	0.7	11	0.8	11	0.8	8	0.7	2	0.3
Cinnamon tea	14	1	14	1	14	1	14	1.1	14	1.1	14	1.3	0	0
Emperor goo	16	1.2	16	1.1	15	1.1	15	1.1	13	1	11	1	7	1
Gadwall (*Ma*	34	2.5	35	2.5	33	2.4	32	2.4	32	2.5	23	2.1	11	1.5
Greater white	18	1.3	16	1.1	16	1.2	18	1.4	15	1.2	8	0.7	11	1.5
Glaucous wi	27	2	27	1.9	27	2	26	2	11	0.8	27	2.4	2	0.3
Green winge	160	11.8	163	11.6	164	12	157	11.8	159	12.2	134	11.9	82	11.1
Laughing gul	14	1	13	0.9	14	1	14	1.1	10	0.8	12	1.1	0	0
Mallard (*Ana*	283	20.9	288	20.6	284	20.8	280	21.1	280	21.4	254	22.6	195	26.5
Northern pin	104	7.7	109	7.8	107	7.9	99	7.5	101	7.7	73	6.5	61	8.3
Red knot (*Ca*	19	1.4	20	1.4	20	1.5	20	1.5	18	1.4	17	1.5	0	0
Ring necked	18	1.3	20	1.4	19	1.4	17	1.3	18	1.4	14	1.3	5	0.7
Ruddy Turns	34	2.5	34	2.4	34	2.5	34	2.6	34	2.6	34	3	12	1.6
Northern sho	121	8.9	122	8.7	120	8.8	117	8.8	121	9.3	95	8.5	64	8.7
Sanderling (C	16	1.2	16	1.1	16	1.2	15	1.1	15	1.2	15	1.3	5	0.7
Snow goose	15	1.1	17	1.2	15	1.1	13	1	13	1	7	0.6	6	0.8
American wi	51	3.8	55	3.9	51	3.7	45	3.4	44	3.4	24	2.1	26	3.5
Rare	102	7.5	113	8.1	102	7.5	102	7.7	94	7.2	84	7.5	39	5.3
Before 2006	93	6.9	93	6.6	93	6.8	93	7	93	7.1	93	8.3	91	12.4

Region	H1		H3		H4		H5		H6		H7		H10		H11	
Canada	n	%	n	%	n	%	n	%	n	%	n	%	n	%	n	%
Alberta	27	9.6	25	4.3	21	4	1	0.5	2	0.6	4	1.2	9	3.2	1	0.4
New Bru	10	3.6	32	5.5	22	4.1	2	0.9	0	0	7	2.1	6	2.1	5	1.8
Nova Sco	7	2.5	14	2.4	9	1.7	0	0	5	1.5	2	0.6	1	0.4	1	0.4
Quebec	3	1.1	5	0.9	3	0.6	3	1.3	0	0	0	0	1	0.4	2	0.7
United States																
Alaska	30	10.7	124	21.2	101	19	16	7.1	19	5.7	24	7.2	36	12.9	14	4.9
Midwest	31	11.1	72	12.3	66	12.4	25	11.2	43	12.8	24	7.2	24	8.6	30	10.5
Northeast	42	15	44	7.5	31	5.8	21	9.4	48	14.3	47	14.1	54	19.3	44	15.4
Northwes	6	2.1	21	3.6	15	2.8	8	3.6	4	1.2	7	2.1	4	1.4	5	1.8
Ohio Val	22	7.9	61	10.4	76	14.3	28	12.5	46	13.7	38	11.4	35	12.5	42	14.7
Northern	5	1.8	9	1.5	10	1.9	6	2.7	5	1.5	10	3	2	0.7	0	0
South	12	4.3	36	6.1	34	6.4	16	7.1	9	2.7	63	18.9	30	10.7	22	7.7
Southwes	0	0	4	0.7	11	2.1	4	1.8	3	0.9	24	7.2	0	0	2	0.7
West	28	10	26	4.4	40	7.5	34	15.2	63	18.8	30	9	35	12.5	39	13.7
Guatemala	4	1.4	8	1.4	8	1.5	5	2.2	0	0	4	1.2	0	0	6	2.1
Rare	4	1.4	19	3.2	12	2.3	17	7.6	6	1.8	9	2.7	3	1.1	6	2.1
Before 2006	49	17.5	86	14.7	72	13.6	38	17	82	24.5	41	12.3	40	14.3	66	23.2
**Host**	**H1**		**H3**		**H4**		**H5**		**H6**		**H7**		**H10**		**H11**	
American bla	12	4.3	23	3.9	22	4.1	5	2.2	2	0.6	7	2.1	5	1.8	7	2.5
Blue winged	13	4.6	93	15.9	86	16.2	7	3.1	16	4.8	31	9.3	18	6.4	20	7
Cinnamon tea	0	0	4	0.7	7	1.3	2	0.9	0	0	6	1.8	1	0.4	2	0.7
Gadwall (*Ma*	0	0	9	1.5	4	0.8	7	3.1	14	4.2	2	0.6	2	0.7	3	1.1
Green winge	23	8.2	71	12.1	57	10.7	21	9.4	35	10.5	44	13.2	21	7.5	24	8.4
Mallard (*Ana*	100	35.7	160	27.3	158	29.8	75	33.5	71	21.2	88	26.4	85	30.4	81	28.4
Northern pin	15	5.4	49	8.4	44	8.3	20	8.9	34	10.2	13	3.9	13	4.6	14	4.9
Red knot (*Ca*	3	1.1	0	0	2	0.4	0	0	0	0	2	0.6	8	2.9	4	1.4
Ruddy Turns	10	3.6	9	1.5	3	0.6	6	2.7	20	6	15	4.5	13	4.6	17	6
Northern sho	13	4.6	24	4.1	28	5.3	11	4.9	15	4.5	51	15.3	31	11.1	20	7
Sanderling (C	4	1.4	4	0.7	0	0	0	0	0	0	5	1.5	6	2.1	4	1.4
American wi	0	0	10	1.7	8	1.5	9	4	19	5.7	4	1.2	2	0.7	0	0
Rare	38	13.6	44	7.5	40	7.5	23	10.3	27	8.1	25	7.5	35	12.5	23	8.1
Before 2006	49	17.5	86	14.7	72	13.6	38	17	82	24.5	41	12.3	40	14.3	66	23.2

Region	N1		N2		N3		N6		N7		N8		N9	
Canada	n	%	n	%	n	%	n	%	n	%	n	%	n	%
Alberta	21	5	3	0.6	9	2.3	20	4	8	3.1	27	4.5	4	1.7
New Bru	10	2.4	5	1.1	3	0.8	23	4.6	12	4.6	28	4.7	6	2.5
Nova Sco	8	1.9	0	0	1	0.3	9	1.8	2	0.8	16	2.7	2	0.8
United States	0	0	0	0	0	0	0	0	0	0	0	0	0	0
Alaska	43	10.2	48	10.2	61	15.3	106	21.2	30	11.5	126	21	24	10.1
Midwest	47	11.2	70	14.8	31	7.8	55	11	17	6.5	78	13	34	14.4
Northeast	66	15.7	51	10.8	52	13	44	8.8	41	15.7	58	9.7	33	13.9
Northwes	10	2.4	11	2.3	4	1	13	2.6	3	1.2	17	2.8	5	2.1
Ohio Val	40	9.5	70	14.8	42	10.5	64	12.8	32	12.2	67	11.2	36	15.2
Northern	8	1.9	4	0.9	6	1.5	10	2	5	1.9	9	1.5	1	0.4
South	24	5.7	37	7.8	60	15	32	6.4	34	13	33	5.5	23	9.7
Southeast	1	0.2	2	0.4	1	0.3	1	0.2	0	0	1	0.2	0	0
Southwes	2	0.5	4	0.9	21	5.3	3	0.6	4	1.5	1	0.2	0	0
West	64	15.2	56	11.9	46	11.5	33	6.6	27	10.3	31	5.2	34	14.4
Guatemala	0	0	10	2.1	16	4	2	0.4	0	0	5	0.8	3	1.3
Rare	7	1.7	21	4.5	20	5	13	2.6	3	1.2	15	2.5	5	2.1
Before 2006	70	16.6	80	17	26	6.5	73	14.6	44	16.8	87	14.5	27	11.4
**Host**	**N1**		**N2**		**N3**		**N6**		**N7**		**N8**		**N9**	
American bla	15	3.6	8	1.7	5	1.3	21	4.2	6	2.3	23	3.8	9	3.8
Blue winged	22	5.2	46	9.8	42	10.5	71	14.2	22	8.4	86	14.4	21	8.9
Canada goos	2	0.5	8	1.7	1	0.3	1	0.2	0	0	0	0	0	0
Cinnamon tea	0	0	0	0	6	1.5	5	1	2	0.8	4	0.7	3	1.3
Emperor goo	2	0.5	6	1.3	0	0	5	1	1	0.4	4	0.7	1	0.4
Gadwall (*Ma*	11	2.6	12	2.5	3	0.8	4	0.8	2	0.8	8	1.3	1	0.4
Greater white	9	2.1	5	1.1	0	0	0	0	3	1.2	3	0.5	0	0
Glaucous wi	1	0.2	7	1.5	25	6.3	5	1	0	0	2	0.3	1	0.4
Green winge	35	8.3	48	10.2	52	13	55	11	25	9.5	67	11.2	28	11.8
Laughing gul	3	0.7	2	0.4	4	1	3	0.6	0	0	1	0.2	3	1.3
Mallard (*Ana*	126	29.9	111	23.5	114	28.6	138	27.5	82	31.3	157	26.2	83	35
Northern pin	28	6.7	38	8.1	21	5.3	45	9	12	4.6	46	7.7	12	5.1
Redhead (*Ay*	3	0.7	2	0.4	1	0.3	4	0.8	3	1.2	3	0.5	0	0
Red knot (*Ca*	3	0.7	1	0.2	2	0.5	4	0.8	4	1.5	0	0	0	0
Ring necked	5	1.2	0	0	0	0	4	0.8	3	1.2	10	1.7	1	0.4
Ruddy Turns	18	4.3	17	3.6	14	3.5	8	1.6	13	5	19	3.2	11	4.6
Northern sho	24	5.7	35	7.4	50	12.5	28	5.6	25	9.5	25	4.2	19	8
Sanderling (C	6	1.4	2	0.4	3	0.8	2	0.4	3	1.2	4	0.7	0	0
Snow goose	5	1.2	2	0.4	4	1	0	0	0	0	3	0.5	1	0.4
American wi	14	3.3	15	3.2	4	1	5	1	2	0.8	10	1.7	0	0
Rare	19	4.5	27	5.7	22	5.5	20	4	10	3.8	37	6.2	16	6.8
Before 2006	70	16.6	80	17	26	6.5	73	14.6	44	16.8	87	14.5	27	11.4

Table E. Cross-tabulation of host and regional distribution of influenza virus gene segments isolated from North American wild birds. Counts and proportions of represented host species by geographic location are compared between the original, unsampled data set (all), the phylogenetic diversity-based sample (PDA) and the stratified random sample (stratified).

Sample	Region	American black duck	American wigeon	Blue-winged teal	Bufflehead	Canada goose	Cinnamon teal	Emperor goose	Gadwall	Glaucous-winged gull	Greater white-fronted goose	Green-winged teal	Laughing gull	Mallard	Northern pintail	Northern shoveler	Red knot	Ring-necked duck	Ruddy turnstone	Sanderling	Snow goose	Grand Total
		Host																				
All	Alaska		10<br>0.2%		3<br>0.1%	2<br>0.0%		22<br>0.5%		87<br>1.9%	12<br>0.3%	117<br>2.6%		336<br>7.3%	388<br>8.5%	56<br>1.2%		2<br>0.0%			3<br>0.1%	1,038<br>22.7%
	Alberta	12<br>0.3%		9<br>0.2%	3<br>0.1%							1<br>0.0%		108<br>2.4%	6<br>0.1%							139<br>3.0%
	British Columbia					1<br>0.0%								5<br>0.1%	1<br>0.0%							7<br>0.2%
	Guatemala		1<br>0.0%	68<br>1.5%								2<br>0.0%				5<br>0.1%						76<br>1.7%
	Midwest	4<br>0.1%	5<br>0.1%	92<br>2.0%	4<br>0.1%	1<br>0.0%			9<br>0.2%			51<br>1.1%		375<br>8.2%	17<br>0.4%	18<br>0.4%		11<br>0.2%				587<br>12.8%
	New Brunswick	105<br>2.3%	3<br>0.1%	14<br>0.3%								14<br>0.3%		33<br>0.7%	1<br>0.0%			2<br>0.0%				172<br>3.8%
	Newfoundland	10<br>0.2%																				10<br>0.2%
	Northeast	8<br>0.2%	1<br>0.0%	1<br>0.0%	4<br>0.1%	3<br>0.1%						3<br>0.1%	23<br>0.5%	276<br>6.0%	1<br>0.0%		23<br>0.5%		339<br>7.4%	33<br>0.7%	10<br>0.2%	725<br>15.8%
	Northwest		2<br>0.0%				9<br>0.2%					10<br>0.2%		45<br>1.0%	1<br>0.0%	14<br>0.3%						81<br>1.8%
	Nova Scotia	8<br>0.2%		12<br>0.3%					1<br>0.0%			3<br>0.1%		17<br>0.4%				1<br>0.0%				42<br>0.9%
	Ohio Valley	10<br>0.2%	5<br>0.1%	96<br>2.1%	4<br>0.1%				11<br>0.2%			94<br>2.1%		282<br>6.2%	19<br>0.4%	37<br>0.8%		1<br>0.0%				559<br>12.2%
	Ontario													8<br>0.2%				1<br>0.0%				9<br>0.2%
	Prince Edward Island	11<br>0.2%		8<br>0.2%								2<br>0.0%										21<br>0.5%
	Quebec	1<br>0.0%												24<br>0.5%				1<br>0.0%			1<br>0.0%	27<br>0.6%
	Rockies & Plains			27<br>0.6%		2<br>0.0%						1<br>0.0%		18<br>0.4%	5<br>0.1%						1<br>0.0%	54<br>1.2%
	Sonora		1<br>0.0%									4<br>0.1%				2<br>0.0%						7<br>0.2%
	South		4<br>0.1%	298<br>6.5%		1<br>0.0%	1<br>0.0%		5<br>0.1%		1<br>0.0%	54<br>1.2%		41<br>0.9%	4<br>0.1%	48<br>1.0%		1<br>0.0%				458<br>10.0%
	Southeast			1<br>0.0%	2<br>0.0%										1<br>0.0%	3<br>0.1%						7<br>0.2%
	Southwest		2<br>0.0%			2<br>0.0%	4<br>0.1%		2<br>0.0%			14<br>0.3%		10<br>0.2%	1<br>0.0%	7<br>0.2%						42<br>0.9%
	West		36<br>0.8%		5<br>0.1%		7<br>0.2%		12<br>0.3%		12<br>0.3%	34<br>0.7%		261<br>5.7%	54<br>1.2%	95<br>2.1%		3<br>0.1%				519<br>11.3%
	Total	169<br>3.7%	70<br>1.5%	626<br>13.7%	25<br>0.5%	12<br>0.3%	21<br>0.5%	22<br>0.5%	40<br>0.9%	87<br>1.9%	25<br>0.5%	404<br>8.8%	23<br>0.5%	1,839<br>40.2%	499<br>10.9%	285<br>6.2%	23<br>0.5%	23<br>0.5%	339<br>7.4%	33<br>0.7%	15<br>0.3%	4,580<br>100.0%
PDA	Alaska		3<br>0.3%		1<br>0.1%			8<br>0.7%		20<br>1.7%	2<br>0.2%	14<br>1.2%		43<br>3.7%	73<br>6.3%	9<br>0.8%					1<br>0.1%	174<br>15.0%
	Alberta	3<br>0.3%		4<br>0.3%	2<br>0.2%									38<br>3.3%								47<br>4.1%
	British Columbia					1<br>0.1%								1<br>0.1%	1<br>0.1%							3<br>0.3%
	Guatemala			26<br>2.2%								1<br>0.1%				3<br>0.3%						30<br>2.6%
	Midwest	1<br>0.1%	2<br>0.2%	21<br>1.8%	1<br>0.1%				1<br>0.1%			17<br>1.5%		86<br>7.4%	2<br>0.2%	4<br>0.3%		3<br>0.3%				138<br>11.9%
	New Brunswick	22<br>1.9%		2<br>0.2%								2<br>0.2%		5<br>0.4%				1<br>0.1%				32<br>2.8%
	Newfoundland	4<br>0.3%																				4<br>0.3%
	Northeast	3<br>0.3%	1<br>0.1%		3<br>0.3%	1<br>0.1%						3<br>0.3%	7<br>0.6%	75<br>6.5%	1<br>0.1%		4<br>0.3%		54<br>4.7%	2<br>0.2%	3<br>0.3%	157<br>13.6%
	Northwest		1<br>0.1%				1<br>0.1%					4<br>0.3%		17<br>1.5%		2<br>0.2%						25<br>2.2%
	Nova Scotia	1<br>0.1%		4<br>0.3%										2<br>0.2%								7<br>0.6%
	Ohio Valley		3<br>0.3%	29<br>2.5%	2<br>0.2%				3<br>0.3%			30<br>2.6%		80<br>6.9%	11<br>1.0%	19<br>1.6%		1<br>0.1%				178<br>15.4%
	Ontario													3<br>0.3%				1<br>0.1%				4<br>0.3%
	Prince Edward Island	1<br>0.1%		1<br>0.1%								1<br>0.1%										3<br>0.3%
	Quebec													6<br>0.5%								6<br>0.5%
	Rockies & Plains			10<br>0.9%		1<br>0.1%								6<br>0.5%	1<br>0.1%						1<br>0.1%	19<br>1.6%
	Sonora		1<br>0.1%									3<br>0.3%				1<br>0.1%						5<br>0.4%
	South		2<br>0.2%	86<br>7.4%		1<br>0.1%			4<br>0.3%			20<br>1.7%		16<br>1.4%	1<br>0.1%	23<br>2.0%		1<br>0.1%				154<br>13.3%
	Southeast			1<br>0.1%											1<br>0.1%	1<br>0.1%						3<br>0.3%
	Southwest		1<br>0.1%			1<br>0.1%	2<br>0.2%		2<br>0.2%			7<br>0.6%		4<br>0.3%		2<br>0.2%						19<br>1.6%
	West		14<br>1.2%				5<br>0.4%		4<br>0.3%		1<br>0.1%	9<br>0.8%		62<br>5.4%	17<br>1.5%	36<br>3.1%		1<br>0.1%				149<br>12.9%
	Total	35<br>3.0%	28<br>2.4%	184<br>15.9%	9<br>0.8%	5<br>0.4%	8<br>0.7%	8<br>0.7%	14<br>1.2%	20<br>1.7%	3<br>0.3%	111<br>9.6%	7<br>0.6%	444<br>38.4%	108<br>9.3%	100<br>8.6%	4<br>0.3%	8<br>0.7%	54<br>4.7%	2<br>0.2%	5<br>0.4%	1,157<br>100.0%
Stratified	Alaska		9<br>0.8%		3<br>0.3%	2<br>0.2%		16<br>1.4%		27<br>2.3%	10<br>0.9%	28<br>2.4%		33<br>2.9%	33<br>2.9%	21<br>1.8%		2<br>0.2%			3<br>0.3%	187<br>16.2%
	Alberta	3<br>0.3%		6<br>0.5%	3<br>0.3%							1<br>0.1%		24<br>2.1%	6<br>0.5%							43<br>3.7%
	British Columbia					1<br>0.1%								4<br>0.3%	1<br>0.1%							6<br>0.5%
	Guatemala		1<br>0.1%	15<br>1.3%								2<br>0.2%				5<br>0.4%						23<br>2.0%
	Midwest	4<br>0.3%	5<br>0.4%	26<br>2.3%	4<br>0.3%	1<br>0.1%			9<br>0.8%			22<br>1.9%		31<br>2.7%	15<br>1.3%	8<br>0.7%		6<br>0.5%				131<br>11.4%
	New Brunswick	12<br>1.0%	3<br>0.3%	5<br>0.4%								7<br>0.6%		9<br>0.8%	1<br>0.1%			2<br>0.2%				39<br>3.4%
	Newfoundland	5<br>0.4%																				5<br>0.4%
	Northeast	8<br>0.7%	1<br>0.1%	1<br>0.1%	3<br>0.3%	3<br>0.3%						3<br>0.3%	14<br>1.2%	36<br>3.1%	1<br>0.1%		19<br>1.6%		34<br>2.9%	16<br>1.4%	9<br>0.8%	148<br>12.8%
	Northwest		2<br>0.2%				3<br>0.3%					7<br>0.6%		14<br>1.2%	1<br>0.1%	5<br>0.4%						32<br>2.8%
	Nova Scotia	7<br>0.6%		3<br>0.3%					1<br>0.1%			3<br>0.3%		7<br>0.6%				1<br>0.1%				22<br>1.9%
	Ohio Valley	10<br>0.9%	5<br>0.4%	28<br>2.4%	4<br>0.3%				9<br>0.8%			30<br>2.6%		34<br>2.9%	17<br>1.5%	19<br>1.6%		1<br>0.1%				157<br>13.6%
	Ontario													5<br>0.4%				1<br>0.1%				6<br>0.5%
	Prince Edward Island	8<br>0.7%		6<br>0.5%								2<br>0.2%										16<br>1.4%
	Quebec	1<br>0.1%												7<br>0.6%				1<br>0.1%			1<br>0.1%	10<br>0.9%
	Rockies & Plains			7<br>0.6%		2<br>0.2%						1<br>0.1%		14<br>1.2%	3<br>0.3%						1<br>0.1%	28<br>2.4%
	Sonora		1<br>0.1%									4<br>0.3%				2<br>0.2%						7<br>0.6%
	South		4<br>0.3%	24<br>2.1%		1<br>0.1%	1<br>0.1%		5<br>0.4%		1<br>0.1%	21<br>1.8%		21<br>1.8%	4<br>0.3%	20<br>1.7%		1<br>0.1%				103<br>8.9%
	Southeast			1<br>0.1%	2<br>0.2%										1<br>0.1%	3<br>0.3%						7<br>0.6%
	Southwest		2<br>0.2%			2<br>0.2%	4<br>0.3%		2<br>0.2%			9<br>0.8%		6<br>0.5%	1<br>0.1%	6<br>0.5%						32<br>2.8%
	West		18<br>1.6%		5<br>0.4%		5<br>0.4%		8<br>0.7%		7<br>0.6%	19<br>1.6%		36<br>3.1%	19<br>1.6%	32<br>2.8%		3<br>0.3%				152<br>13.2%
	Total	58<br>5.0%	51<br>4.4%	122<br>10.6%	24<br>2.1%	12<br>1.0%	13<br>1.1%	16<br>1.4%	34<br>2.9%	27<br>2.3%	18<br>1.6%	159<br>13.8%	14<br>1.2%	281<br>24.4%	103<br>8.9%	121<br>10.5%	19<br>1.6%	18<br>1.6%	34<br>2.9%	16<br>1.4%	14<br>1.2%	1,154<br>100.0%

Table F. Hemagglutinin and neuraminidase subtype by host order. Counts across all segments and subtypes are compared between the original, unsampled data set (all), the phylogenetic diversity-based sample (PDA) and the stratified random sample (stratified).

HA	NA	All		PDA		Stratified	
		Anseriformes	Charadriiformes	Anseriformes	Charadriiformes	Anseriformes	Charadriiformes
H1	N1	1,269	116	559	35	519	44
	N2	90	0	39	0	38	0
	N3	137	9	70	3	61	7
	N5	13	15	7	4	4	4
	N6	14	2	7	0	4	0
	N7	7	25	3	12	7	2
	N8	50	20	20	10	27	8
	N9	44	29	27	10	22	7
	Mixed or missing	23	2	18	2	16	2
H2	N1	28	0	10	0	18	0
	N2	54	0	22	0	28	0
	N3	435	0	173	0	114	0
	N4	11	0	3	0	6	0
	N5	29	0	12	0	14	0
	N6	18	0	6	0	6	0
	N7	33	0	9	0	4	0
	N8	2	0	1	0	1	0
	N9	93	0	40	0	38	0
H3	N1	121	0	53	0	38	0
	N2	714	37	280	7	232	3
	N3	49	0	23	0	15	0
	N4	1	0	0	0	0	0
	N5	19	3	5	0	3	1
	N6	561	57	217	13	177	21
	N7	48	12	13	1	14	1
	N8	4,283	102	1,399	25	1,418	35
	N9	35	0	13	0	17	0
	Mixed or missing	153	0	63	0	58	0
H4	N1	41	0	18	0	9	0
	N2	273	0	136	0	114	0
	N3	43	0	25	0	22	0
	N4	24	0	9	0	7	0
	N5	45	0	14	0	9	0
	N6	3,798	173	1,257	31	1,110	26
	N7	26	1	11	0	10	0
	N8	924	0	387	0	330	0
	N9	55	0	15	0	21	0
	Mixed or missing	135	1	70	1	62	0
H5	N1	141	37	60	14	73	7
	N2	1,022	24	455	12	546	14
	N3	141	6	82	3	102	6
	N4	21	0	10	0	5	0
	N5	102	0	21	0	12	0
	N6	10	0	1	0	1	0
	N7	7	0	4	0	5	0
	N8	27	0	6	0	12	0
	N9	69	7	38	3	38	3
	Mixed or missing	18	0	13	0	9	0
H6	N1	931	63	421	35	465	23
	N2	483	21	247	13	246	9
	N3	7	0	4	0	0	0
	N4	17	0	9	0	6	0
	N5	113	6	56	2	53	1
	N6	16	7	11	1	11	0
	N7	14	7	3	2	5	2
	N8	237	46	127	21	113	23
	N9	12	0	9	0	5	0
	Mixed or missing	21	3	18	3	15	2
H7	N1	108	0	46	0	32	0
	N2	57	12	35	6	34	3
	N3	1,441	363	712	60	684	108
	N4	60	7	30	4	31	0
	N5	6	0	6	0	2	0
	N6	56	0	26	0	23	0
	N7	390	18	150	8	165	7
	N8	41	0	20	0	26	0
	N9	86	0	57	0	62	0
	Mixed or missing	28	4	19	1	20	1
H8	N1	6	0	6	0	5	0
	N2	6	0	5	0	5	0
	N3	5	0	5	0	2	0
	N4	372	5	81	4	76	1
	N8	6	0	4	0	0	0
	Mixed or missing	4	0	0	0	2	0
H9	N1	11	6	6	6	3	2
	N2	151	28	87	10	92	12
	N3	6	0	5	0	0	0
	N4	5	0	4	0	4	0
H10	N1	46	51	27	19	13	16
	N2	34	28	20	12	18	4
	N3	257	0	117	0	104	0
	N4	29	31	7	8	5	11
	N5	27	173	10	29	6	38
	N6	51	16	30	10	19	14
	N7	1,123	322	422	89	391	65
	N8	45	23	16	5	14	5
	N9	33	20	18	11	13	4
	Mixed or missing	20	10	14	5	14	2
H11	N1	7	49	4	10	6	33
	N2	234	53	127	23	123	17
	N3	112	0	70	0	62	0
	N5	6	21	3	6	3	0
	N6	11	10	9	2	3	4
	N7	0	22	0	10	0	13
	N8	0	52	0	19	0	24
	N9	961	59	516	37	411	15
	Mixed or missing	47	1	29	1	29	1
H12	N1	0	22	0	7	0	4
	N2	11	0	8	0	8	0
	N3	0	10	0	4	0	6
	N4	19	40	10	12	9	5
	N5	325	105	113	8	71	26
	N6	12	0	5	0	4	0
	N8	23	0	9	0	10	0
	N9	8	0	2	0	1	0
	Mixed or missing	8	0	1	0	2	0
H13	N2	0	42	0	16	0	9
	N6	0	90	0	30	0	41
	N8	0	5	0	0	0	2
	N9	5	22	3	7	5	16
H14	N2	12	0	8	0	8	0
	N3	51	0	16	0	17	0
	N4	6	0	1	0	4	0
	N5	5	0	3	0	0	0
	N6	17	0	6	0	5	0
	N7	12	0	9	0	6	0
H16	N3	18	307	8	97	11	113
Mixed or missing	N1	49	3	35	2	36	1
	N2	57	3	41	3	32	2
	N3	44	3	29	1	28	2
	N4	12	0	0	0	4	0
	N5	5	0	2	0	0	0
	N6	162	4	70	0	66	1
	N7	26	4	18	2	21	0
	N8	153	1	77	0	67	1
	N9	30	0	26	0	22	0
	Mixed or missing	1,947	116	682	36	526	10

Table G. Host species transition rate matrix from combined internal gene model. Median rates and 95% highest posterior density intervals are displayed for both subsampling strategies. Rates colored in blue are statistically supported (Bayes factor > 100). (ABD – American black duck, BUF – bufflehead, BWT – blue-winged teal, CAN – Canada goose, CIN – cinnamon teal, EMP – emperor goose, GAD – gadwall, GWF – greater white-fronted goose, GWG – glaucous-winged gull, GWT – green-winged teal, LAU – laughing gull, MAL – mallard, PIN – northern pintail, RED – redhead, RKN – red knot, RND – ring-necked duck, RUD – ruddy turnstone, SHO – northern shoveler, SND – sanderling, SNO – snow goose, WIG – American wigeon).

		Sink																			
Source	Sample	ABD	BUF	BWT	CAN	CIN	EMP	GAD	GWF	GWG	GWT	LAU	MAL	PIN	RKN	RND	RUD	SHO	SND	SNO	WIG
ABD	PDA		0.3 [0.0, 1.1]	0.4 [0.0, 1.6]	0.2 [0.0, 0.7]	0.3 [0.0, 0.8]	0.2 [0.0, 0.7]	0.2 [0.0, 1.2]	0.1 [0.0, 0.8]	0.3 [0.0, 1.3]	0.3 [0.0, 1.1]	0.3 [0.0, 1.1]	**2.6 [0.6, 5.1]**	0.5 [0.0, 1.4]	0.4 [0.0, 1.0]	0.2 [0.0, 0.8]	1.6 [0.0, 3.6]	0.2 [0.0, 0.8]	0.2 [0.0, 1.0]	0.5 [0.0, 1.4]	0.2 [0.0, 0.7]
	Stratified		0.5 [0.0, 1.3]	1.8 [0.3, 3.6]	0.2 [0.0, 0.9]	0.1 [0.0, 0.5]	0.2 [0.0, 0.6]	**1.5 [0.5, 2.7]**	0.2 [0.0, 0.7]	0.2 [0.0, 0.6]	**3.7 [1.6, 6.3]**	0.3 [0.0, 0.9]	**8.2 [4.5, 12.2]**	0.7 [0.0, 1.8]	0.4 [0.0, 1.1]	1.0 [0.2, 2.1]	1.0 [0.1, 2.3]	0.3 [0.0, 0.9]	0.3 [0.0, 0.9]	0.9 [0.1, 2.0]	0.6 [0.0, 1.6]
BUF	PDA	0.1 [0.0, 1.0]		0.3 [0.0, 1.0]	0.1 [0.0, 0.8]	0.2 [0.0, 1.0]	0.2 [0.0, 0.7]	0.2 [0.0, 0.5]	0.1 [0.0, 0.6]	0.2 [0.0, 0.8]	0.2 [0.0, 0.8]	0.2 [0.0, 0.7]	0.2 [0.0, 1.2]	0.2 [0.0, 1.1]	0.2 [0.0, 0.9]	0.2 [0.0, 0.6]	0.3 [0.0, 0.9]	0.2 [0.0, 1.3]	0.2 [0.0, 0.7]	0.2 [0.0, 0.9]	0.2 [0.0, 0.7]
	Stratified	0.2 [0.0, 0.9]		0.6 [0.0, 1.8]	0.1 [0.0, 0.6]	0.2 [0.0, 0.7]	0.3 [0.0, 0.8]	0.2 [0.0, 0.6]	0.1 [0.0, 0.5]	0.1 [0.0, 0.9]	0.6 [0.0, 1.6]	0.2 [0.0, 0.5]	0.7 [0.0, 2.0]	0.2 [0.0, 0.9]	0.1 [0.0, 0.5]	0.1 [0.0, 0.5]	0.3 [0.0, 1.0]	0.3 [0.0, 1.3]	0.1 [0.0, 0.4]	0.3 [0.0, 0.9]	**1.4 [0.5, 2.6]**
BWT	PDA	0.7 [0.0, 1.8]	1.0 [0.1, 2.0]		0.1 [0.0, 0.4]	0.2 [0.0, 0.8]	0.3 [0.0, 0.9]	**1.6 [0.5, 2.9]**	0.4 [0.0, 1.0]	0.4 [0.0, 1.4]	**9.3 [6.1, 12.8]**	0.3 [0.0, 0.8]	**40.7 [32.5, 49.2]**	1.2 [0.0, 2.8]	0.5 [0.0, 1.2]	0.3 [0.0, 0.9]	1.3 [0.0, 2.7]	**9.1 [5.7, 12.9]**	0.3 [0.0, 1.0]	0.2 [0.0, 0.8]	1.2 [0.2, 2.5]
	Stratified	**2.9 [1.3, 4.8]**	**2.3 [1.1, 3.7]**		0.7 [0.1, 1.5]	0.4 [0.0, 1.0]	0.1 [0.0, 0.7]	**1.8 [0.8, 3.1]**	0.1 [0.0, 0.5]	0.2 [0.0, 0.8]	**13.5 [9.6, 18.0]**	0.1 [0.0, 0.2]	**21.2 [15.7, 27.4]**	**5.8 [3.5, 8.6]**	0.4 [0.0, 1.0]	**1.2 [0.3, 2.0]**	**2.2 [0.9, 3.8]**	**5.5 [2.6, 8.9]**	0.2 [0.0, 0.5]	0.9 [0.2, 1.8]	0.7 [0.0, 1.6]
CAN	PDA	0.1 [0.0, 0.8]	0.2 [0.0, 1.1]	0.2 [0.0, 1.1]		0.2 [0.0, 0.8]	0.3 [0.0, 0.7]	0.2 [0.0, 0.7]	0.2 [0.0, 0.9]	0.3 [0.0, 1.1]	0.2 [0.0, 0.8]	0.2 [0.0, 0.8]	0.2 [0.0, 1.3]	0.3 [0.0, 0.7]	0.2 [0.0, 1.0]	0.1 [0.0, 0.7]	0.3 [0.0, 1.0]	0.2 [0.0, 0.8]	0.2 [0.0, 0.7]	0.2 [0.0, 0.7]	0.1 [0.0, 0.9]
	Stratified	0.2 [0.0, 0.9]	0.1 [0.0, 0.7]	0.2 [0.0, 0.9]		0.2 [0.0, 0.8]	0.2 [0.0, 0.5]	0.1 [0.0, 0.8]	0.4 [0.0, 1.3]	0.2 [0.0, 0.9]	0.4 [0.0, 1.4]	0.2 [0.0, 0.7]	0.3 [0.0, 1.0]	0.3 [0.0, 1.2]	0.2 [0.0, 0.7]	0.3 [0.0, 1.0]	0.1 [0.0, 0.6]	0.2 [0.0, 0.7]	0.2 [0.0, 0.6]	0.3 [0.0, 1.0]	0.1 [0.0, 0.9]
CIN	PDA	0.1 [0.0, 0.8]	0.3 [0.0, 1.0]	0.4 [0.0, 0.9]	0.2 [0.0, 0.9]		0.2 [0.0, 0.9]	0.3 [0.0, 1.0]	0.2 [0.0, 1.0]	0.2 [0.0, 0.9]	0.2 [0.0, 0.9]	0.2 [0.0, 0.6]	0.3 [0.0, 0.8]	0.3 [0.0, 1.0]	0.2 [0.0, 0.7]	0.2 [0.0, 0.8]	0.2 [0.0, 0.7]	0.3 [0.0, 1.1]	0.3 [0.0, 1.0]	0.1 [0.0, 0.5]	0.2 [0.0, 1.1]
	Stratified	0.1 [0.0, 0.4]	0.1 [0.0, 0.6]	0.3 [0.0, 0.9]	0.1 [0.0, 0.5]		0.2 [0.0, 0.5]	0.3 [0.0, 0.8]	0.2 [0.0, 0.6]	0.1 [0.0, 0.7]	0.7 [0.0, 1.6]	0.1 [0.0, 0.6]	0.4 [0.0, 1.4]	0.6 [0.0, 1.6]	0.1 [0.0, 0.6]	0.1 [0.0, 0.6]	0.2 [0.0, 0.7]	0.2 [0.0, 0.9]	0.1 [0.0, 0.4]	0.1 [0.0, 0.4]	0.2 [0.0, 0.7]
EMP	PDA	0.3 [0.0, 0.9]	0.2 [0.0, 0.8]	0.3 [0.0, 1.4]	0.2 [0.0, 0.7]	0.2 [0.0, 0.7]		0.2 [0.0, 0.9]	0.2 [0.0, 0.9]	0.2 [0.0, 1.0]	0.2 [0.0, 1.0]	0.3 [0.0, 1.0]	0.3 [0.0, 1.3]	0.2 [0.0, 1.0]	0.2 [0.0, 0.8]	0.2 [0.0, 0.7]	0.1 [0.0, 0.6]	0.2 [0.0, 0.7]	0.2 [0.0, 0.8]	0.2 [0.0, 0.8]	0.1 [0.0, 0.8]
	Stratified	0.2 [0.0, 0.6]	0.3 [0.0, 1.0]	0.2 [0.0, 0.6]	0.2 [0.0, 0.5]	0.1 [0.0, 0.5]		0.1 [0.0, 0.6]	0.3 [0.0, 1.0]	0.4 [0.0, 1.0]	0.8 [0.0, 2.0]	0.2 [0.0, 0.7]	0.4 [0.0, 1.4]	0.6 [0.0, 1.9]	0.2 [0.0, 0.6]	0.2 [0.0, 0.8]	0.1 [0.0, 0.6]	0.3 [0.0, 0.9]	0.2 [0.0, 0.6]	0.3 [0.0, 1.0]	0.2 [0.0, 0.8]
GAD	PDA	0.2 [0.0, 0.8]	0.2 [0.0, 0.8]	0.2 [0.0, 1.3]	0.1 [0.0, 0.8]	0.3 [0.0, 1.0]	0.3 [0.0, 0.8]		0.2 [0.0, 1.1]	0.2 [0.0, 0.8]	0.3 [0.0, 1.3]	0.3 [0.0, 1.2]	0.2 [0.0, 1.0]	0.3 [0.0, 1.1]	0.2 [0.0, 0.9]	0.2 [0.0, 1.0]	0.3 [0.0, 1.1]	0.3 [0.0, 1.3]	0.2 [0.0, 0.8]	0.2 [0.0, 0.8]	0.3 [0.0, 1.2]
	Stratified	0.6 [0.0, 1.9]	0.1 [0.0, 0.6]	0.6 [0.0, 1.8]	0.1 [0.0, 0.9]	0.2 [0.0, 0.8]	0.2 [0.0, 0.4]		0.5 [0.0, 1.7]	0.3 [0.0, 1.0]	0.9 [0.0, 2.3]	0.1 [0.0, 0.6]	0.9 [0.0, 2.5]	0.8 [0.0, 2.3]	0.1 [0.0, 0.6]	0.4 [0.0, 1.6]	0.3 [0.0, 1.1]	0.8 [0.0, 2.4]	0.1 [0.0, 0.6]	0.2 [0.0, 0.7]	0.7 [0.0, 3.1]
GWF	PDA	0.1 [0.0, 0.8]	0.2 [0.0, 0.9]	0.2 [0.0, 0.9]	0.2 [0.0, 1.1]	0.2 [0.0, 0.6]	0.2 [0.0, 1.0]	0.2 [0.0, 0.7]		0.2 [0.0, 1.2]	0.2 [0.0, 1.0]	0.2 [0.0, 0.7]	0.3 [0.0, 1.1]	0.2 [0.0, 0.9]	0.2 [0.0, 0.8]	0.2 [0.0, 0.8]	0.1 [0.0, 0.7]	0.3 [0.0, 0.9]	0.2 [0.0, 0.7]	0.3 [0.0, 1.2]	0.1 [0.0, 0.7]
	Stratified	0.2 [0.0, 0.5]	0.1 [0.0, 0.7]	0.1 [0.0, 0.9]	0.5 [0.0, 1.3]	0.2 [0.0, 0.9]	0.2 [0.0, 0.7]	0.4 [0.0, 1.4]		0.3 [0.0, 0.9]	0.3 [0.0, 1.0]	0.2 [0.0, 0.7]	0.4 [0.0, 1.5]	0.6 [0.0, 1.7]	0.2 [0.0, 0.7]	0.3 [0.0, 1.2]	0.1 [0.0, 0.7]	0.7 [0.0, 1.9]	0.2 [0.0, 0.6]	0.3 [0.0, 1.0]	0.4 [0.0, 1.3]
GWG	PDA	0.2 [0.0, 0.8]	0.2 [0.0, 0.6]	0.3 [0.0, 1.3]	0.1 [0.0, 0.6]	0.2 [0.0, 0.8]	0.2 [0.0, 0.7]	0.1 [0.0, 0.5]	0.2 [0.0, 0.7]		0.2 [0.0, 0.7]	0.2 [0.0, 0.6]	0.8 [0.1, 1.9]	0.8 [0.0, 2.0]	0.5 [0.0, 1.3]	0.1 [0.0, 0.6]	0.3 [0.0, 1.2]	0.2 [0.0, 0.7]	0.2 [0.0, 0.5]	0.2 [0.0, 0.6]	0.2 [0.0, 0.7]
	Stratified	0.1 [0.0, 0.6]	0.1 [0.0, 0.5]	0.2 [0.0, 0.7]	0.1 [0.0, 0.4]	0.2 [0.0, 0.4]	0.1 [0.0, 0.5]	0.1 [0.0, 0.5]	0.1 [0.0, 0.5]		0.1 [0.0, 0.6]	0.1 [0.0, 0.5]	0.1 [0.0, 0.6]	**0.7 [0.1, 1.4]**	0.1 [0.0, 0.5]	0.1 [0.0, 0.5]	0.1 [0.0, 0.7]	0.1 [0.0, 0.5]	0.1 [0.0, 0.4]	0.1 [0.0, 0.4]	0.2 [0.0, 0.5]
GWT	PDA	0.7 [0.0, 1.9]	0.2 [0.0, 0.9]	1.3 [0.0, 3.6]	0.2 [0.0, 0.8]	1.1 [0.1, 2.4]	0.4 [0.1, 1.1]	0.6 [0.0, 1.8]	0.3 [0.0, 1.1]	0.9 [0.0, 2.7]		0.3 [0.0, 1.1]	4.3 [0.4, 8.8]	2.2 [0.2, 5.0]	0.2 [0.0, 0.5]	0.2 [0.0, 1.1]	0.5 [0.0, 1.4]	2.5 [0.3, 5.1]	0.6 [0.0, 1.6]	0.1 [0.0, 0.7]	0.6 [0.0, 1.5]
	Stratified	0.8 [0.0, 2.1]	**1.7 [0.7, 2.9]**	**7.6 [4.1, 11.1]**	0.3 [0.0, 0.9]	**3.4 [2.1, 4.8]**	**2.3 [1.2, 3.6]**	1.3 [0.3, 2.3]	0.2 [0.0, 0.8]	**1.2 [0.5, 2.0]**		0.0 [0.0, 0.2]	**31.6 [24.6, 38.4]**	**10.0 [6.6, 13.7]**	0.6 [0.1, 1.3]	0.5 [0.0, 1.1]	0.8 [0.0, 1.8]	**16.8 [11.9, 21.6]**	0.2 [0.0, 0.6]	0.5 [0.0, 1.0]	**3.7 [1.8, 5.8]**
LAU	PDA	0.3 [0.0, 1.3]	0.2 [0.0, 0.8]	0.1 [0.0, 1.0]	0.2 [0.0, 0.7]	0.3 [0.0, 0.8]	0.2 [0.0, 0.7]	0.4 [0.0, 1.0]	0.2 [0.0, 0.8]	0.2 [0.0, 1.0]	0.2 [0.0, 0.8]		0.3 [0.0, 1.3]	0.2 [0.0, 0.9]	0.2 [0.0, 0.8]	0.2 [0.0, 1.1]	0.3 [0.0, 1.0]	0.2 [0.0, 0.9]	0.2 [0.0, 0.9]	0.2 [0.0, 0.8]	0.2 [0.0, 0.8]
	Stratified	0.2 [0.0, 0.7]	0.2 [0.0, 0.4]	0.2 [0.0, 0.7]	0.2 [0.0, 0.5]	0.2 [0.0, 0.6]	0.1 [0.0, 0.5]	0.2 [0.0, 0.7]	0.3 [0.0, 0.7]	0.2 [0.0, 1.1]	0.3 [0.0, 0.7]		0.2 [0.0, 1.0]	0.2 [0.0, 0.6]	0.1 [0.0, 0.6]	0.1 [0.0, 0.5]	0.3 [0.0, 1.1]	0.2 [0.0, 0.7]	0.5 [0.0, 1.5]	0.2 [0.0, 0.6]	0.2 [0.0, 0.9]
MAL	PDA	**6.5 [5.4, 7.6]**	**1.5 [0.9, 2.1]**	**33.3 [30.0, 37.0]**	**1.1 [0.7, 1.6]**	**1.6 [1.0, 2.2]**	**0.8 [0.4, 1.2]**	**2.2 [1.5, 2.9]**	**1.0 [0.6, 1.4]**	**0.7 [0.3, 1.1]**	**21.1 [18.6, 23.7]**	0.2 [0.0, 0.4]		**16.5 [14.4, 18.8]**	**0.8 [0.4, 1.2]**	**1.1 [0.7, 1.5]**	**8.1 [6.6, 9.4]**	**22.6 [19.7, 25.7]**	0.3 [0.1, 0.5]	**1.1 [0.7, 1.6]**	**4.2 [3.2, 5.1]**
	Stratified	**8.7 [7.2, 10.3]**	**1.6 [0.9, 2.4]**	**16.7 [13.8, 19.5]**	**1.8 [1.2, 2.5]**	0.5 [0.1, 0.9]	0.4 [0.0, 0.8]	**2.0 [1.1, 3.1]**	**1.1 [0.6, 1.8]**	0.2 [0.0, 0.6]	**21.7 [18.3, 25.1]**	0.0 [0.0, 0.1]		**12.9 [10.6, 15.5]**	0.6 [0.2, 1.1]	**2.1 [1.4, 3.0]**	**4.4 [3.3, 5.6]**	**16.5 [13.5, 19.8]**	0.1 [0.0, 0.4]	**1.5 [0.8, 2.2]**	**5.5 [4.1, 6.9]**
PIN	PDA	0.1 [0.0, 0.5]	0.2 [0.0, 0.7]	0.4 [0.0, 1.4]	0.2 [0.0, 0.8]	0.1 [0.0, 0.6]	**1.9 [0.8, 3.2]**	0.2 [0.0, 1.0]	0.4 [0.0, 1.1]	**2.4 [1.1, 4.0]**	**4.9 [2.5, 7.5]**	0.1 [0.0, 0.6]	**25.8 [17.8, 33.7]**		0.5 [0.0, 1.3]	0.3 [0.0, 0.8]	**1.6 [0.5, 2.9]**	0.2 [0.0, 0.7]	0.1 [0.0, 0.4]	0.7 [0.0, 1.8]	0.4 [0.0, 1.2]
	Stratified	0.1 [0.0, 0.8]	1.1 [0.3, 2.2]	1.0 [0.0, 2.8]	0.6 [0.0, 1.4]	0.4 [0.0, 1.3]	**3.0 [1.4, 4.7]**	**1.8 [0.7, 3.2]**	**2.2 [1.1, 3.5]**	**1.2 [0.3, 2.3]**	**9.1 [5.3, 13.6]**	0.1 [0.0, 0.3]	**16.3 [10.3, 22.2]**		**0.8 [0.2, 1.6]**	0.4 [0.0, 1.0]	1.0 [0.1, 2.1]	0.5 [0.0, 1.6]	0.6 [0.0, 1.3]	**0.7 [0.1, 1.4]**	0.3 [0.0, 0.9]
RKN	PDA	0.2 [0.0, 0.6]	0.2 [0.0, 0.9]	0.2 [0.0, 1.0]	0.2 [0.0, 0.8]	0.2 [0.0, 0.9]	0.1 [0.0, 1.0]	0.2 [0.0, 0.9]	0.2 [0.0, 1.1]	0.2 [0.0, 0.8]	0.2 [0.0, 1.0]	0.2 [0.0, 0.8]	0.2 [0.0, 1.2]	0.5 [0.0, 1.4]		0.2 [0.0, 0.8]	0.2 [0.0, 1.2]	0.3 [0.0, 1.0]	0.3 [0.0, 1.2]	0.2 [0.0, 0.8]	0.2 [0.0, 0.8]
	Stratified	0.2 [0.0, 0.5]	0.1 [0.0, 0.6]	0.2 [0.0, 0.7]	0.2 [0.0, 1.1]	0.3 [0.0, 0.9]	0.2 [0.0, 0.9]	0.1 [0.0, 0.5]	0.2 [0.0, 0.6]	0.2 [0.0, 1.0]	0.2 [0.0, 0.8]	0.5 [0.0, 1.3]	0.2 [0.0, 1.1]	0.6 [0.0, 1.9]		0.2 [0.0, 0.6]	0.8 [0.0, 2.0]	0.2 [0.0, 0.8]	**0.9 [0.1, 1.9]**	0.2 [0.0, 0.6]	0.2 [0.0, 1.0]
RND	PDA	0.2 [0.0, 0.8]	0.2 [0.0, 0.7]	0.2 [0.0, 1.0]	0.2 [0.0, 0.8]	0.2 [0.0, 0.9]	0.1 [0.0, 0.6]	0.1 [0.0, 0.6]	0.2 [0.0, 0.6]	0.2 [0.0, 1.1]	0.1 [0.0, 0.7]	0.1 [0.0, 0.8]	0.3 [0.0, 1.0]	0.3 [0.0, 0.9]	0.2 [0.0, 0.8]		0.3 [0.0, 1.0]	0.2 [0.0, 0.7]	0.2 [0.0, 0.8]	0.1 [0.0, 0.8]	0.2 [0.0, 1.0]
	Stratified	0.4 [0.0, 1.4]	0.3 [0.0, 1.0]	0.3 [0.0, 1.2]	0.2 [0.0, 1.0]	0.2 [0.0, 0.7]	0.1 [0.0, 0.9]	0.4 [0.0, 1.2]	0.4 [0.0, 1.2]	0.2 [0.0, 0.6]	0.6 [0.0, 1.8]	0.2 [0.0, 0.7]	0.7 [0.0, 1.9]	0.8 [0.0, 2.2]	0.2 [0.0, 0.7]		0.2 [0.0, 1.0]	0.5 [0.0, 1.4]	0.2 [0.0, 0.7]	0.2 [0.0, 0.7]	0.3 [0.0, 1.1]
RUD	PDA	1.0 [0.1, 2.4]	0.2 [0.0, 0.6]	0.8 [0.0, 2.1]	0.2 [0.0, 0.7]	0.3 [0.0, 1.0]	0.2 [0.0, 0.9]	0.3 [0.0, 1.0]	0.2 [0.0, 0.9]	0.6 [0.0, 1.6]	0.2 [0.0, 0.9]	**3.1 [1.5, 4.9]**	1.6 [0.2, 3.6]	**2.8 [0.8, 5.3]**	**1.6 [0.5, 2.9]**	0.1 [0.0, 0.7]		0.2 [0.0, 0.9]	**1.2 [0.4, 2.4]**	0.3 [0.0, 1.0]	0.4 [0.0, 1.0]
	Stratified	0.2 [0.0, 0.8]	0.1 [0.0, 0.6]	0.1 [0.0, 0.5]	0.3 [0.0, 0.8]	0.3 [0.0, 0.7]	0.2 [0.0, 1.0]	0.2 [0.0, 0.7]	0.2 [0.0, 0.7]	0.2 [0.0, 0.8]	0.2 [0.0, 0.7]	**5.9 [3.9, 8.0]**	0.8 [0.0, 2.2]	**9.8 [6.8, 13.0]**	**9.7 [7.2, 12.6]**	0.2 [0.0, 0.5]		0.3 [0.0, 0.8]	**11.4 [8.8, 14.4]**	0.2 [0.0, 0.6]	0.1 [0.0, 0.4]
SHO	PDA	0.2 [0.0, 1.0]	0.2 [0.0, 1.0]	1.2 [0.0, 3.5]	0.2 [0.0, 0.8]	1.3 [0.2, 2.7]	0.4 [0.0, 1.2]	0.4 [0.0, 1.5]	0.3 [0.0, 1.2]	0.2 [0.0, 1.0]	1.5 [0.0, 3.9]	0.3 [0.0, 1.1]	**7.6 [2.7, 13.3]**	**32.5 [24.9, 39.6]**	0.3 [0.0, 1.0]	0.4 [0.0, 1.2]	0.3 [0.0, 1.4]		0.4 [0.0, 1.4]	1.1 [0.0, 2.5]	0.9 [0.0, 2.3]
	Stratified	0.5 [0.0, 1.5]	0.7 [0.1, 1.7]	**6.3 [3.2, 9.7]**	0.4 [0.0, 1.0]	**1.7 [0.7, 2.8]**	0.3 [0.0, 0.9]	**3.3 [2.0, 4.9]**	**1.1 [0.3, 2.1]**	0.3 [0.0, 0.9]	**11.6 [6.9, 16.6]**	0.1 [0.0, 0.4]	**20.9 [14.9, 27.5]**	**14.1 [10.6, 17.7]**	0.2 [0.0, 0.7]	**1.5 [0.6, 2.6]**	0.4 [0.0, 1.3]		0.1 [0.0, 0.4]	0.2 [0.0, 0.7]	**2.9 [1.4, 4.6]**
SND	PDA	0.2 [0.0, 0.8]	0.2 [0.0, 0.5]	0.3 [0.0, 0.9]	0.2 [0.0, 0.8]	0.2 [0.0, 1.0]	0.1 [0.0, 0.8]	0.3 [0.0, 0.8]	0.2 [0.0, 0.7]	0.2 [0.0, 1.0]	0.2 [0.0, 1.0]	0.2 [0.0, 0.8]	0.2 [0.0, 0.8]	0.2 [0.0, 0.9]	0.2 [0.0, 1.0]	0.2 [0.0, 1.0]	0.2 [0.0, 1.0]	0.3 [0.0, 1.2]		0.1 [0.0, 0.7]	0.2 [0.0, 0.7]
	Stratified	0.3 [0.0, 0.9]	0.1 [0.0, 0.5]	0.2 [0.0, 0.7]	0.1 [0.0, 0.5]	0.2 [0.0, 0.7]	0.1 [0.0, 1.0]	0.1 [0.0, 0.6]	0.2 [0.0, 0.8]	0.2 [0.0, 1.0]	0.2 [0.0, 0.7]	1.0 [0.1, 2.2]	0.3 [0.0, 1.0]	0.9 [0.0, 2.5]	0.6 [0.0, 1.6]	0.1 [0.0, 0.4]	0.3 [0.0, 1.2]	0.1 [0.0, 0.9]		0.2 [0.0, 0.6]	0.2 [0.0, 0.5]
SNO	PDA	0.3 [0.0, 0.9]	0.2 [0.0, 0.9]	0.3 [0.0, 0.9]	0.2 [0.0, 0.8]	0.2 [0.0, 0.8]	0.2 [0.0, 0.9]	0.2 [0.0, 1.2]	0.3 [0.0, 1.0]	0.2 [0.0, 1.0]	0.3 [0.0, 1.0]	0.2 [0.0, 0.8]	0.3 [0.0, 1.3]	0.7 [0.0, 2.3]	0.2 [0.0, 0.7]	0.2 [0.0, 0.8]	0.3 [0.0, 0.9]	0.3 [0.0, 1.0]	0.2 [0.0, 1.0]		0.2 [0.0, 0.7]
	Stratified	0.4 [0.0, 1.4]	0.2 [0.0, 0.6]	0.6 [0.0, 1.7]	0.2 [0.0, 0.9]	0.1 [0.0, 0.5]	0.3 [0.0, 0.9]	0.3 [0.0, 0.8]	0.5 [0.0, 1.3]	0.2 [0.0, 1.0]	0.3 [0.0, 1.7]	0.3 [0.0, 0.9]	0.8 [0.0, 2.0]	0.4 [0.0, 1.7]	0.2 [0.0, 0.4]	0.2 [0.0, 1.0]	0.2 [0.0, 1.0]	0.7 [0.0, 1.6]	0.2 [0.0, 0.5]		0.2 [0.0, 0.6]
WIG	PDA	0.1 [0.0, 0.7]	0.2 [0.0, 1.2]	0.3 [0.0, 1.0]	0.1 [0.0, 0.6]	0.2 [0.0, 0.9]	0.3 [0.0, 0.9]	0.4 [0.0, 1.5]	0.4 [0.0, 1.3]	0.3 [0.0, 1.3]	0.2 [0.0, 0.8]	0.1 [0.0, 0.8]	0.3 [0.0, 1.4]	0.5 [0.0, 2.2]	0.1 [0.0, 1.1]	0.2 [0.0, 1.1]	0.3 [0.0, 0.8]	0.3 [0.0, 1.4]	0.1 [0.0, 0.9]	0.2 [0.0, 0.8]	
	Stratified	0.4 [0.0, 1.3]	0.2 [0.0, 0.8]	0.3 [0.0, 1.1]	0.4 [0.0, 1.1]	0.2 [0.0, 0.7]	0.2 [0.0, 0.9]	**4.1 [1.8, 6.4]**	**2.3 [0.8, 4.0]**	0.2 [0.0, 0.7]	1.4 [0.2, 3.0]	0.1 [0.0, 0.4]	**3.7 [1.3, 6.6]**	**2.1 [0.7, 4.0]**	0.1 [0.0, 0.5]	1.0 [0.2, 2.0]	0.2 [0.0, 0.6]	1.3 [0.2, 2.9]	0.2 [0.0, 0.6]	0.2 [0.0, 0.7]	

Table H. Host species transition rate matrix from combined hemagglutinin subtype model. Median rates and 95% highest posterior density intervals are displayed for both subsampling strategies. Rates colored in blue are statistically supported (Bayes factor > 100). (ABD – American black duck, BUF – bufflehead, BWT – blue-winged teal, CAN – Canada goose, CIN – cinnamon teal, EMP – emperor goose, GAD – gadwall, GWF – greater white-fronted goose, GWG – glaucous-winged gull, GWT – green-winged teal, LAU – laughing gull, MAL – mallard, PIN – northern pintail, RED – redhead, RKN – red knot, RND – ring-necked duck, RUD – ruddy turnstone, SHO – northern shoveler, SND – sanderling, SNO – snow goose, WIG – American wigeon).

		Sink											
Source	Sample	ABD	BWT	CIN	GAD	GWT	MAL	PIN	RKN	RUD	SHO	SND	WIG
ABD	PDA		0.6 [0.0, 1.9]	0.2 [0.0, 1.0]	0.3 [0.0, 1.0]	0.9 [0.0, 2.2]	1.4 [0.0, 3.3]	0.3 [0.0, 1.2]	0.2 [0.0, 0.8]	0.5 [0.0, 1.6]	0.3 [0.0, 1.1]	0.2 [0.0, 1.0]	0.3 [0.0, 0.8]
	Stratified		0.7 [0.0, 2.3]	0.2 [0.0, 0.9]	0.6 [0.0, 1.7]	0.8 [0.0, 2.2]	2.1 [0.1, 5.1]	0.3 [0.0, 1.1]	0.2 [0.0, 1.0]	0.3 [0.0, 1.2]	0.3 [0.0, 1.3]	0.2 [0.0, 0.8]	0.3 [0.0, 1.0]
BWT	PDA	0.7 [0.0, 1.7]		0.4 [0.0, 1.1]	0.3 [0.0, 0.9]	**3.7 [1.9, 5.7]**	**22.0 [16.4, 27.4]**	0.7 [0.0, 1.8]	0.1 [0.0, 0.4]	0.4 [0.0, 1.1]	**2.2 [0.5, 4.1]**	0.1 [0.0, 0.5]	0.4 [0.0, 1.0]
	Stratified	0.9 [0.0, 2.1]		0.2 [0.0, 0.8]	0.5 [0.0, 1.4]	**5.0 [2.3, 8.1]**	**10.9 [6.3, 16.3]**	0.6 [0.0, 1.8]	0.1 [0.0, 0.6]	0.2 [0.0, 0.9]	1.1 [0.1, 2.6]	0.1 [0.0, 0.5]	0.2 [0.0, 0.7]
CIN	PDA	0.2 [0.0, 1.0]	0.3 [0.0, 1.1]		0.2 [0.0, 1.0]	0.5 [0.0, 1.4]	0.2 [0.0, 1.1]	0.3 [0.0, 1.1]	0.2 [0.0, 0.9]	0.2 [0.0, 0.9]	0.3 [0.0, 1.2]	0.2 [0.0, 1.0]	0.3 [0.0, 1.0]
	Stratified	0.2 [0.0, 0.9]	0.3 [0.0, 1.0]		0.4 [0.0, 1.2]	0.8 [0.0, 2.2]	0.5 [0.0, 1.7]	0.2 [0.0, 1.1]	0.2 [0.0, 0.9]	0.2 [0.0, 0.9]	0.3 [0.0, 1.3]	0.2 [0.0, 0.9]	0.2 [0.0, 0.9]
GAD	PDA	0.3 [0.0, 1.1]	0.3 [0.0, 1.3]	0.3 [0.0, 1.1]		0.2 [0.0, 0.9]	0.4 [0.0, 1.6]	0.5 [0.0, 1.7]	0.3 [0.0, 1.0]	0.2 [0.0, 1.1]	0.7 [0.0, 2.0]	0.2 [0.0, 0.9]	0.5 [0.0, 1.6]
	Stratified	0.5 [0.0, 1.7]	0.4 [0.0, 1.5]	0.3 [0.0, 1.0]		0.4 [0.0, 1.7]	0.3 [0.0, 1.4]	0.5 [0.0, 1.7]	0.2 [0.0, 0.9]	0.2 [0.0, 1.0]	0.6 [0.0, 1.7]	0.2 [0.0, 0.9]	0.3 [0.0, 1.4]
GWT	PDA	0.4 [0.0, 1.6]	1.2 [0.0, 3.5]	0.6 [0.0, 1.9]	0.2 [0.0, 0.9]		**6.4 [2.5, 10.7]**	1.1 [0.0, 3.0]	0.2 [0.0, 0.7]	0.4 [0.0, 1.4]	1.2 [0.0, 4.4]	0.2 [0.0, 0.8]	0.4 [0.0, 1.4]
	Stratified	0.8 [0.0, 2.1]	2.5 [0.3, 5.2]	1.2 [0.0, 2.6]	0.8 [0.0, 1.9]		**10.6 [5.2, 16.1]**	**7.5 [3.8, 11.4]**	0.1 [0.0, 0.6]	0.3 [0.0, 1.4]	6.1 [0.7, 10.3]	0.1 [0.0, 0.6]	0.5 [0.0, 1.5]
MAL	PDA	**3.2 [2.2, 4.4]**	**10.0 [7.7, 12.6]**	**0.7 [0.2, 1.2]**	**1.3 [0.6, 2.1]**	**9.7 [7.6, 12.1]**		**10.4 [8.2, 12.9]**	0.1 [0.0, 0.3]	**2.6 [1.6, 3.6]**	**8.9 [6.7, 11.4]**	0.1 [0.0, 0.3]	**2.8 [1.8, 3.9]**
	Stratified	**4.1 [2.7, 5.5]**	**11.8 [9.3, 14.5]**	**1.1 [0.4, 1.8]**	**1.7 [0.9, 2.7]**	**14.5 [11.5, 17.5]**		**7.5 [5.4, 9.7]**	0.1 [0.0, 0.2]	**2.8 [1.8, 3.8]**	**10.7 [7.7, 13.9]**	0.1 [0.0, 0.3]	**2.9 [1.9, 4.0]**
PIN	PDA	0.2 [0.0, 0.9]	0.3 [0.0, 1.2]	0.2 [0.0, 0.8]	0.2 [0.0, 0.8]	**5.2 [2.8, 7.9]**	**8.4 [4.5, 12.6]**		0.2 [0.0, 0.6]	0.6 [0.0, 1.9]	0.4 [0.0, 1.2]	0.5 [0.0, 1.4]	0.5 [0.0, 1.4]
	Stratified	0.3 [0.0, 1.1]	0.5 [0.0, 1.7]	0.2 [0.0, 1.0]	0.5 [0.0, 1.4]	1.8 [0.0, 4.4]	**6.2 [3.0, 10.6]**		0.3 [0.0, 1.1]	0.4 [0.0, 1.4]	0.3 [0.0, 0.9]	0.5 [0.0, 1.5]	0.3 [0.0, 1.0]
RKN	PDA	0.2 [0.0, 0.9]	0.2 [0.0, 1.0]	0.2 [0.0, 0.9]	0.2 [0.0, 1.0]	0.2 [0.0, 1.0]	0.2 [0.0, 1.0]	0.3 [0.0, 1.2]		0.3 [0.0, 1.0]	0.3 [0.0, 1.0]	0.3 [0.0, 1.0]	0.2 [0.0, 1.0]
	Stratified	0.2 [0.0, 0.9]	0.2 [0.0, 1.0]	0.2 [0.0, 0.9]	0.2 [0.0, 1.1]	0.3 [0.0, 1.0]	0.3 [0.0, 1.2]	0.5 [0.0, 1.9]		0.4 [0.0, 1.4]	0.2 [0.0, 1.1]	0.4 [0.0, 1.3]	0.2 [0.0, 0.9]
RUD	PDA	0.2 [0.0, 0.8]	0.3 [0.0, 1.2]	0.2 [0.0, 0.8]	0.2 [0.0, 0.8]	0.4 [0.0, 1.3]	0.9 [0.0, 2.2]	**4.0 [2.0, 6.6]**	**2.5 [1.2, 4.2]**		0.2 [0.0, 1.0]	**3.2 [1.5, 5.1]**	0.2 [0.0, 0.8]
	Stratified	0.2 [0.0, 0.8]	0.3 [0.0, 0.9]	0.2 [0.0, 0.7]	0.2 [0.0, 0.8]	0.5 [0.0, 1.4]	0.5 [0.0, 1.6]	**6.3 [3.6, 9.4]**	**3.4 [1.7, 5.4]**		0.2 [0.0, 1.1]	**4.4 [2.3, 6.6]**	0.2 [0.0, 0.8]
SHO	PDA	0.2 [0.0, 0.7]	2.6 [0.3, 5.5]	**1.2 [0.3, 2.5]**	**1.9 [0.5, 3.7]**	**6.8 [3.8, 10.2]**	**5.8 [2.0, 10.2]**	**5.5 [2.4, 8.9]**	0.2 [0.0, 0.8]	0.5 [0.0, 1.4]		0.2 [0.0, 0.8]	1.3 [0.1, 2.9]
	Stratified	0.3 [0.0, 1.0]	0.5 [0.0, 2.1]	0.6 [0.0, 1.7]	**1.5 [0.3, 3.1]**	2.4 [0.0, 7.6]	3.6 [0.2, 8.5]	**8.3 [4.1, 12.8]**	0.2 [0.0, 0.7]	0.3 [0.0, 1.1]		0.2 [0.0, 0.8]	**1.3 [0.2, 2.6]**
SND	PDA	0.2 [0.0, 1.0]	0.2 [0.0, 1.0]	0.2 [0.0, 0.9]	0.2 [0.0, 1.0]	0.2 [0.0, 1.0]	0.2 [0.0, 1.1]	0.8 [0.0, 2.0]	0.4 [0.0, 1.3]	0.4 [0.0, 1.4]	0.3 [0.0, 1.1]		0.2 [0.0, 1.1]
	Stratified	0.2 [0.0, 1.0]	0.2 [0.0, 0.9]	0.2 [0.0, 1.0]	0.2 [0.0, 0.8]	0.3 [0.0, 1.0]	0.3 [0.0, 1.1]	0.9 [0.0, 2.3]	0.4 [0.0, 1.4]	0.3 [0.0, 1.4]	0.3 [0.0, 1.1]		0.2 [0.0, 1.0]
WIG	PDA	0.2 [0.0, 1.0]	0.4 [0.0, 1.5]	0.4 [0.0, 1.3]	0.6 [0.0, 1.9]	0.3 [0.0, 1.3]	0.4 [0.0, 1.6]	0.7 [0.0, 2.4]	0.2 [0.0, 0.9]	0.2 [0.0, 1.0]	0.6 [0.0, 2.0]	0.2 [0.0, 0.9]	
	Stratified	0.2 [0.0, 0.9]	0.2 [0.0, 1.0]	0.2 [0.0, 1.0]	0.4 [0.0, 1.4]	0.4 [0.0, 1.2]	0.6 [0.0, 2.3]	0.5 [0.0, 1.8]	0.3 [0.0, 1.0]	0.3 [0.0, 0.9]	0.4 [0.0, 1.3]	0.2 [0.0, 0.9]	

Table I. Host species transition rate matrix from combined neuraminidase subtype model. Median rates and 95% highest posterior density intervals are displayed for both subsampling strategies. Rates colored in blue are statistically supported (Bayes factor > 100). (ABD – American black duck, BUF – bufflehead, BWT – blue-winged teal, CAN – Canada goose, CIN – cinnamon teal, EMP – emperor goose, GAD – gadwall, GWF – greater white-fronted goose, GWG – glaucous-winged gull, GWT – green-winged teal, LAU – laughing gull, MAL – mallard, PIN – northern pintail, RED – redhead, RKN – red knot, RND – ring-necked duck, RUD – ruddy turnstone, SHO – northern shoveler, SND – sanderling, SNO – snow goose, WIG – American wigeon).

		Sink																			
Source	Sample	ABD	BWT	CAN	CIN	EMP	GAD	GWF	GWG	GWT	LAU	MAL	PIN	RED	RKN	RND	RUD	SHO	SND	SNO	WIG
ABD	PDA		1.1 [0.0, 3.2]	0.2 [0.0, 1.3]	0.3 [0.0, 1.2]	0.4 [0.0, 1.3]	0.2 [0.0, 1.1]	0.3 [0.0, 1.5]	0.2 [0.0, 1.0]	**2.9 [0.9, 5.6]**	0.3 [0.0, 1.0]	**3.0 [0.5, 6.6]**	0.5 [0.0, 1.7]	0.3 [0.0, 1.1]	0.3 [0.0, 1.1]	0.3 [0.0, 1.3]	1.0 [0.0, 2.7]	0.6 [0.0, 1.8]	0.3 [0.0, 1.2]	0.4 [0.0, 1.5]	0.3 [0.0, 1.0]
	Stratified		0.6 [0.0, 2.2]	0.3 [0.0, 1.2]	0.2 [0.0, 1.7]	0.4 [0.0, 1.2]	0.5 [0.0, 1.6]	0.2 [0.0, 1.2]	0.2 [0.0, 1.2]	2.8 [0.4, 5.8]	0.3 [0.0, 1.1]	4.2 [0.1, 8.7]	0.7 [0.0, 2.2]	0.4 [0.0, 1.6]	0.3 [0.0, 1.4]	0.4 [0.0, 1.5]	1.0 [0.0, 2.7]	0.5 [0.0, 1.8]	0.3 [0.0, 1.2]	0.3 [0.0, 1.2]	0.3 [0.0, 1.4]
BWT	PDA	1.8 [0.4, 3.5]		0.2 [0.0, 0.8]	0.7 [0.0, 1.6]	0.3 [0.0, 1.0]	0.1 [0.0, 0.7]	0.2 [0.0, 0.7]	0.2 [0.0, 0.7]	**7.6 [4.5, 11.3]**	0.2 [0.0, 0.8]	**21.2 [15.0, 28.0]**	**2.2 [0.6, 4.3]**	**1.1 [0.3, 2.2]**	0.1 [0.0, 0.4]	0.2 [0.0, 0.9]	0.8 [0.0, 1.9]	**3.8 [1.1, 6.6]**	0.2 [0.0, 0.7]	0.7 [0.0, 1.5]	0.4 [0.0, 1.2]
	Stratified	1.4 [0.2, 3.0]		0.3 [0.0, 0.9]	0.6 [0.0, 1.5]	0.2 [0.0, 0.7]	1.3 [0.1, 2.6]	0.1 [0.0, 0.6]	0.1 [0.0, 1.2]	**9.0 [5.0, 13.7]**	0.1 [0.0, 0.3]	**22.6 [14.2, 31.7]**	1.3 [0.0, 3.2]	**1.6 [0.5, 2.9]**	0.2 [0.0, 0.7]	1.2 [0.3, 2.5]	1.1 [0.0, 2.6]	2.5 [0.4, 5.3]	0.2 [0.0, 0.6]	0.8 [0.0, 1.9]	0.7 [0.0, 1.6]
CAN	PDA	0.3 [0.0, 1.0]	0.3 [0.0, 1.8]		0.3 [0.0, 1.9]	0.3 [0.0, 1.3]	0.3 [0.0, 1.3]	0.3 [0.0, 1.2]	0.4 [0.0, 1.3]	0.2 [0.0, 1.2]	0.3 [0.0, 1.3]	0.4 [0.0, 1.4]	0.4 [0.0, 1.8]	0.4 [0.0, 1.4]	0.3 [0.0, 1.3]	0.3 [0.0, 1.5]	0.3 [0.0, 1.4]	0.3 [0.0, 1.3]	0.2 [0.0, 1.4]	0.4 [0.0, 2.1]	0.3 [0.0, 1.3]
	Stratified	0.3 [0.0, 1.5]	0.6 [0.0, 1.7]		0.3 [0.0, 1.3]	0.3 [0.0, 1.3]	0.2 [0.0, 1.1]	0.4 [0.0, 1.6]	0.2 [0.0, 1.3]	0.3 [0.0, 1.4]	0.3 [0.0, 1.4]	0.5 [0.0, 1.7]	0.3 [0.0, 1.0]	0.4 [0.0, 1.5]	0.3 [0.0, 1.3]	0.3 [0.0, 1.4]	0.3 [0.0, 1.2]	0.4 [0.0, 1.3]	0.3 [0.0, 1.6]	0.3 [0.0, 1.5]	0.3 [0.0, 1.0]
CIN	PDA	0.2 [0.0, 1.1]	0.4 [0.0, 1.7]	0.4 [0.0, 1.6]		0.3 [0.0, 1.3]	0.3 [0.0, 1.2]	0.4 [0.0, 1.7]	0.3 [0.0, 1.5]	0.5 [0.0, 2.0]	0.2 [0.0, 1.3]	0.8 [0.0, 2.2]	0.4 [0.0, 1.3]	0.3 [0.0, 1.3]	0.3 [0.0, 1.1]	0.3 [0.0, 1.2]	0.4 [0.0, 1.4]	0.4 [0.0, 1.4]	0.5 [0.0, 1.2]	0.7 [0.0, 2.1]	0.5 [0.0, 1.7]
	Stratified	0.3 [0.0, 1.3]	0.4 [0.0, 1.8]	0.6 [0.0, 1.7]		0.3 [0.0, 1.5]	0.6 [0.0, 2.0]	0.3 [0.0, 1.2]	0.3 [0.0, 1.4]	1.5 [0.0, 3.9]	0.4 [0.0, 1.2]	0.5 [0.0, 2.2]	0.3 [0.0, 1.2]	0.3 [0.0, 1.6]	0.4 [0.0, 1.5]	0.4 [0.0, 1.2]	0.3 [0.0, 1.1]	0.6 [0.0, 2.0]	0.3 [0.0, 1.2]	0.6 [0.0, 2.0]	0.4 [0.0, 1.6]
EMP	PDA	0.3 [0.0, 1.3]	0.3 [0.0, 1.5]	0.3 [0.0, 1.2]	0.4 [0.0, 2.0]		0.6 [0.0, 1.8]	0.3 [0.0, 1.2]	0.5 [0.0, 1.6]	0.3 [0.0, 1.1]	0.3 [0.0, 1.3]	0.5 [0.0, 1.8]	0.5 [0.0, 1.6]	0.3 [0.0, 1.2]	0.4 [0.0, 1.6]	0.4 [0.0, 1.2]	0.3 [0.0, 0.9]	0.4 [0.0, 1.7]	0.4 [0.0, 1.5]	0.3 [0.0, 1.3]	0.4 [0.0, 1.6]
	Stratified	0.2 [0.0, 1.0]	0.4 [0.0, 1.4]	0.2 [0.0, 0.8]	0.3 [0.0, 1.1]		0.2 [0.0, 0.9]	0.4 [0.0, 1.5]	0.5 [0.0, 1.8]	0.6 [0.0, 2.0]	0.3 [0.0, 1.0]	0.5 [0.0, 1.7]	0.5 [0.0, 2.1]	0.2 [0.0, 0.9]	0.3 [0.0, 1.3]	0.2 [0.0, 1.1]	0.3 [0.0, 1.4]	0.3 [0.0, 1.3]	0.2 [0.0, 1.0]	0.2 [0.0, 1.3]	0.4 [0.0, 1.3]
GAD	PDA	0.4 [0.0, 1.5]	0.5 [0.0, 1.8]	0.3 [0.0, 1.3]	0.3 [0.0, 1.2]	0.2 [0.0, 1.3]		0.6 [0.0, 2.8]	0.4 [0.0, 1.5]	0.4 [0.0, 2.1]	0.4 [0.0, 1.6]	0.8 [0.0, 2.3]	0.6 [0.0, 1.7]	0.2 [0.0, 1.1]	0.2 [0.0, 1.2]	0.3 [0.0, 1.3]	0.3 [0.0, 0.9]	0.4 [0.0, 1.7]	0.3 [0.0, 1.2]	0.4 [0.0, 1.3]	0.4 [0.0, 1.9]
	Stratified	0.6 [0.0, 2.1]	0.7 [0.0, 2.3]	0.3 [0.0, 1.3]	0.4 [0.0, 1.1]	0.3 [0.0, 1.5]		0.5 [0.0, 1.8]	0.5 [0.0, 1.4]	0.5 [0.0, 2.0]	0.4 [0.0, 1.3]	1.1 [0.0, 3.4]	0.7 [0.0, 2.1]	0.4 [0.0, 1.4]	0.3 [0.0, 1.6]	0.7 [0.0, 2.3]	0.4 [0.0, 1.4]	0.6 [0.0, 2.1]	0.3 [0.0, 1.1]	0.5 [0.0, 2.0]	0.7 [0.0, 2.2]
GWF	PDA	0.3 [0.0, 1.3]	0.3 [0.0, 1.4]	0.3 [0.0, 1.3]	0.4 [0.0, 1.3]	0.3 [0.0, 1.0]	0.8 [0.0, 2.2]		0.5 [0.0, 1.8]	0.6 [0.0, 2.1]	0.2 [0.0, 1.4]	0.5 [0.0, 2.0]	0.6 [0.0, 2.1]	0.3 [0.0, 1.3]	0.3 [0.0, 1.3]	0.7 [0.0, 2.2]	0.5 [0.0, 1.3]	0.7 [0.0, 2.6]	0.3 [0.0, 1.2]	0.3 [0.0, 1.2]	0.9 [0.0, 2.7]
	Stratified	0.3 [0.0, 1.0]	0.4 [0.0, 1.5]	0.5 [0.0, 1.6]	0.3 [0.0, 0.9]	0.3 [0.0, 1.2]	0.3 [0.0, 1.6]		0.4 [0.0, 1.7]	0.3 [0.0, 1.6]	0.3 [0.0, 0.9]	0.6 [0.0, 2.3]	0.4 [0.0, 1.8]	0.2 [0.0, 1.0]	0.3 [0.0, 1.6]	0.6 [0.0, 2.3]	0.3 [0.0, 1.3]	0.7 [0.0, 2.3]	0.2 [0.0, 1.5]	0.2 [0.0, 1.0]	0.5 [0.0, 1.8]
GWG	PDA	0.3 [0.0, 1.1]	0.3 [0.0, 1.3]	0.2 [0.0, 0.9]	0.2 [0.0, 0.9]	0.3 [0.0, 1.6]	0.3 [0.0, 1.0]	0.2 [0.0, 0.9]		0.4 [0.0, 1.3]	0.2 [0.0, 1.1]	0.6 [0.0, 2.3]	0.8 [0.0, 2.2]	0.3 [0.0, 1.5]	0.3 [0.0, 1.3]	0.4 [0.0, 1.5]	0.4 [0.0, 1.3]	0.4 [0.0, 1.2]	0.3 [0.0, 1.3]	0.2 [0.0, 1.9]	0.3 [0.0, 1.7]
	Stratified	0.4 [0.0, 1.6]	0.5 [0.0, 1.7]	0.2 [0.0, 1.1]	0.4 [0.0, 1.2]	0.5 [0.0, 1.6]	0.3 [0.0, 1.2]	0.2 [0.0, 1.1]		0.6 [0.0, 1.8]	0.3 [0.0, 1.3]	0.8 [0.0, 2.2]	0.8 [0.0, 2.1]	0.2 [0.0, 1.0]	0.3 [0.0, 1.4]	0.2 [0.0, 1.0]	0.2 [0.0, 0.9]	0.6 [0.0, 2.0]	0.3 [0.0, 1.0]	0.3 [0.0, 1.2]	0.2 [0.0, 0.9]
GWT	PDA	1.1 [0.0, 3.5]	2.7 [0.0, 7.2]	0.7 [0.0, 2.1]	**1.9 [0.3, 4.2]**	0.5 [0.0, 1.4]	0.6 [0.0, 1.8]	0.4 [0.0, 1.7]	0.3 [0.0, 1.2]		0.3 [0.0, 1.2]	**9.3 [3.3, 17.0]**	1.7 [0.0, 4.6]	0.6 [0.0, 2.2]	0.2 [0.0, 1.1]	0.2 [0.0, 1.7]	0.6 [0.0, 2.6]	**6.8 [2.8, 11.3]**	0.2 [0.0, 0.9]	0.8 [0.0, 2.4]	0.6 [0.0, 2.1]
	Stratified	1.3 [0.0, 3.4]	**7.4 [2.5, 12.9]**	0.6 [0.0, 1.5]	**2.4 [0.5, 4.4]**	0.7 [0.0, 2.0]	**1.7 [0.4, 3.3]**	0.3 [0.0, 1.1]	0.3 [0.0, 1.2]		0.3 [0.0, 1.0]	**19.7 [11.3, 28.1]**	**6.8 [3.1, 11.0]**	0.7 [0.0, 1.7]	0.3 [0.0, 0.8]	0.2 [0.0, 0.7]	1.0 [0.0, 2.5]	**9.7 [4.5, 15.4]**	0.1 [0.0, 0.6]	0.5 [0.0, 1.7]	0.8 [0.0, 2.2]
LAU	PDA	0.4 [0.0, 1.3]	0.3 [0.0, 1.4]	0.3 [0.0, 1.5]	0.2 [0.0, 0.9]	0.3 [0.0, 1.2]	0.3 [0.0, 1.2]	0.3 [0.0, 1.4]	0.2 [0.0, 1.2]	0.4 [0.0, 1.2]		0.3 [0.0, 1.4]	0.3 [0.0, 1.3]	0.2 [0.0, 1.1]	0.5 [0.0, 1.6]	0.3 [0.0, 1.2]	0.4 [0.0, 1.7]	0.3 [0.0, 1.3]	0.3 [0.0, 1.3]	0.4 [0.0, 1.3]	0.3 [0.0, 1.3]
	Stratified	0.3 [0.0, 1.5]	0.5 [0.0, 1.8]	0.3 [0.0, 1.4]	0.2 [0.0, 1.1]	0.3 [0.0, 1.4]	0.3 [0.0, 1.1]	0.3 [0.0, 1.3]	0.4 [0.0, 1.4]	0.4 [0.0, 1.5]		0.4 [0.0, 1.5]	0.4 [0.0, 1.7]	0.2 [0.0, 1.1]	0.4 [0.0, 1.8]	0.3 [0.0, 1.1]	0.4 [0.0, 1.3]	0.3 [0.0, 1.2]	0.7 [0.0, 2.1]	0.3 [0.0, 1.0]	0.4 [0.0, 1.6]
MAL	PDA	**3.4 [2.2, 4.7]**	**16.3 [13.1, 19.7]**	**0.5 [0.1, 0.9]**	0.6 [0.1, 1.1]	0.2 [0.0, 0.6]	**1.5 [0.8, 2.2]**	**0.7 [0.3, 1.4]**	0.1 [0.0, 0.3]	**11.8 [9.2, 14.5]**	0.1 [0.0, 0.4]		**8.7 [6.4, 11.0]**	0.3 [0.0, 0.8]	0.1 [0.0, 0.2]	**0.5 [0.1, 0.9]**	**3.8 [2.6, 5.1]**	**8.0 [5.7, 10.3]**	0.0 [0.0, 0.3]	0.4 [0.1, 0.8]	**2.5 [1.6, 3.5]**
	Stratified	**5.1 [3.5, 6.9]**	**11.5 [8.0, 15.0]**	**0.7 [0.2, 1.3]**	**0.6 [0.1, 1.3]**	0.3 [0.0, 0.7]	**1.5 [0.6, 2.6]**	**1.0 [0.4, 1.8]**	0.1 [0.0, 0.4]	**14.4 [11.1, 18.0]**	0.1 [0.0, 0.3]		**10.3 [7.6, 12.8]**	0.4 [0.0, 0.9]	0.1 [0.0, 0.3]	**1.2 [0.5, 2.1]**	**3.5 [2.1, 4.8]**	**10.2 [7.5, 13.5]**	0.1 [0.0, 0.3]	0.6 [0.1, 1.1]	**3.4 [2.1, 4.8]**
PIN	PDA	0.2 [0.0, 0.8]	0.3 [0.0, 1.3]	0.1 [0.0, 0.7]	0.3 [0.0, 1.1]	**2.0 [0.8, 3.5]**	0.2 [0.0, 0.6]	0.3 [0.0, 1.1]	**1.4 [0.5, 2.6]**	**8.8 [5.5, 12.4]**	0.3 [0.0, 0.9]	**20.3 [14.2, 26.6]**		0.2 [0.0, 0.8]	0.4 [0.0, 1.2]	0.2 [0.0, 0.9]	1.0 [0.0, 2.4]	0.5 [0.0, 1.5]	0.3 [0.0, 0.9]	0.6 [0.0, 1.6]	0.6 [0.0, 1.7]
	Stratified	0.7 [0.0, 2.0]	0.8 [0.0, 2.8]	0.4 [0.0, 1.3]	0.2 [0.0, 0.9]	**3.4 [1.6, 5.6]**	0.5 [0.0, 1.4]	0.4 [0.0, 1.4]	**2.4 [0.9, 4.1]**	**7.9 [4.1, 12.2]**	0.4 [0.0, 1.3]	**9.3 [4.1, 15.0]**		0.2 [0.0, 0.9]	0.5 [0.0, 1.4]	0.3 [0.0, 1.2]	0.8 [0.0, 2.2]	0.8 [0.0, 2.5]	0.3 [0.0, 0.9]	0.6 [0.0, 1.6]	0.6 [0.0, 1.6]
RED	PDA	0.3 [0.0, 1.4]	0.4 [0.0, 1.6]	0.2 [0.0, 0.9]	0.3 [0.0, 1.3]	0.3 [0.0, 1.1]	0.3 [0.0, 1.3]	0.3 [0.0, 1.5]	0.3 [0.0, 1.5]	0.5 [0.0, 1.4]	0.4 [0.0, 1.8]	0.2 [0.0, 1.4]	0.3 [0.0, 1.7]		0.6 [0.0, 1.9]	0.2 [0.0, 0.8]	0.2 [0.0, 1.1]	0.3 [0.0, 1.0]	0.3 [0.0, 1.4]	0.3 [0.0, 1.4]	0.2 [0.0, 1.5]
	Stratified	0.4 [0.0, 1.4]	0.5 [0.0, 1.8]	0.3 [0.0, 1.5]	0.4 [0.1, 1.6]	0.2 [0.0, 1.4]	0.3 [0.0, 1.0]	0.4 [0.0, 1.2]	0.3 [0.0, 1.3]	0.4 [0.0, 1.7]	0.3 [0.0, 1.6]	0.4 [0.0, 1.5]	0.4 [0.0, 1.5]		0.3 [0.0, 1.1]	0.3 [0.0, 1.1]	0.2 [0.0, 1.2]	0.3 [0.0, 1.5]	0.3 [0.0, 1.3]	0.3 [0.0, 1.2]	0.4 [0.0, 1.4]
RKN	PDA	0.3 [0.0, 1.3]	0.4 [0.0, 1.5]	0.4 [0.0, 1.5]	0.3 [0.0, 1.3]	0.3 [0.0, 1.6]	0.4 [0.0, 1.2]	0.4 [0.0, 1.5]	0.3 [0.0, 1.7]	0.3 [0.0, 1.5]	0.3 [0.0, 1.2]	0.4 [0.0, 1.4]	0.4 [0.0, 1.5]	0.3 [0.0, 1.2]		0.3 [0.0, 1.2]	0.4 [0.0, 1.5]	0.4 [0.0, 1.7]	0.4 [0.0, 1.4]	0.3 [0.0, 0.9]	0.3 [0.0, 1.2]
	Stratified	0.4 [0.0, 1.4]	0.3 [0.0, 1.4]	0.4 [0.0, 1.4]	0.3 [0.0, 1.6]	0.2 [0.0, 0.8]	0.4 [0.0, 1.2]	0.3 [0.0, 1.0]	0.3 [0.0, 1.3]	0.3 [0.0, 1.6]	0.5 [0.0, 1.7]	0.5 [0.0, 1.9]	0.4 [0.0, 2.0]	0.2 [0.0, 1.4]		0.3 [0.0, 1.7]	0.4 [0.0, 1.5]	0.4 [0.0, 1.3]	0.3 [0.0, 1.1]	0.3 [0.0, 1.3]	0.2 [0.0, 1.3]
RND	PDA	0.3 [0.0, 1.2]	0.4 [0.0, 1.3]	0.4 [0.0, 1.6]	0.4 [0.0, 1.3]	0.3 [0.0, 1.3]	0.4 [0.0, 1.1]	0.3 [0.0, 1.4]	0.4 [0.0, 1.1]	0.4 [0.0, 1.5]	0.2 [0.0, 1.5]	0.3 [0.0, 1.3]	0.3 [0.0, 1.4]	0.3 [0.0, 1.4]	0.3 [0.0, 1.4]		0.4 [0.0, 1.6]	0.3 [0.0, 1.1]	0.3 [0.0, 1.2]	0.3 [0.0, 1.2]	0.5 [0.0, 1.7]
	Stratified	0.6 [0.0, 2.1]	0.9 [0.0, 2.7]	0.4 [0.0, 1.3]	0.3 [0.0, 1.4]	0.3 [0.0, 1.0]	0.9 [0.0, 2.6]	0.9 [0.0, 2.6]	0.3 [0.0, 1.3]	0.4 [0.0, 1.4]	0.3 [0.0, 1.3]	0.9 [0.0, 2.6]	0.4 [0.0, 1.9]	0.4 [0.0, 1.5]	0.2 [0.0, 1.2]		0.4 [0.0, 1.6]	0.8 [0.0, 2.5]	0.2 [0.0, 1.0]	0.3 [0.0, 1.2]	0.6 [0.0, 1.9]
RUD	PDA	0.6 [0.0, 1.8]	0.5 [0.0, 1.8]	0.6 [0.0, 1.7]	0.2 [0.0, 1.2]	0.3 [0.0, 1.5]	0.3 [0.0, 1.0]	0.4 [0.0, 1.3]	0.2 [0.0, 1.0]	0.9 [0.0, 2.5]	**1.8 [0.4, 3.4]**	1.4 [0.0, 3.6]	**2.6 [0.5, 5.4]**	0.3 [0.0, 0.9]	**2.6 [1.1, 4.7]**	0.3 [0.0, 1.3]		0.6 [0.0, 1.9]	**1.9 [0.5, 3.6]**	0.2 [0.0, 1.1]	0.3 [0.0, 1.3]
	Stratified	0.2 [0.0, 1.3]	0.3 [0.0, 1.4]	0.6 [0.0, 1.7]	0.2 [0.0, 0.9]	0.2 [0.0, 0.8]	0.2 [0.0, 1.3]	0.2 [0.0, 1.3]	0.2 [0.0, 0.8]	0.8 [0.0, 2.4]	**2.9 [1.2, 5.1]**	0.9 [0.0, 2.6]	**4.2 [1.6, 7.3]**	0.2 [0.0, 1.1]	**4.2 [2.0, 6.6]**	0.3 [0.0, 1.1]		0.4 [0.0, 1.4]	**5.3 [2.9, 8.1]**	0.2 [0.0, 0.9]	0.3 [0.0, 1.3]
SHO	PDA	0.2 [0.0, 1.0]	2.0 [0.0, 6.6]	0.6 [0.0, 1.9]	0.6 [0.0, 2.3]	0.7 [0.0, 1.9]	0.9 [0.0, 2.4]	1.2 [0.1, 2.7]	0.5 [0.0, 1.6]	1.6 [0.1, 3.8]	0.2 [0.0, 1.0]	**9.0 [3.5, 15.4]**	**6.6 [2.9, 10.9]**	0.2 [0.0, 0.9]	0.3 [0.0, 1.2]	0.4 [0.0, 1.4]	0.3 [0.0, 1.1]		0.2 [0.0, 0.9]	0.3 [0.0, 0.9]	1.2 [0.0, 3.1]
	Stratified	0.2 [0.0, 1.1]	**6.7 [2.6, 11.5]**	0.5 [0.0, 1.6]	0.7 [0.0, 2.0]	0.4 [0.0, 1.4]	**2.1 [0.5, 3.8]**	1.0 [0.0, 2.4]	0.6 [0.0, 1.7]	**5.5 [1.2, 10.3]**	0.3 [0.0, 0.9]	**10.9 [4.5, 18.1]**	**6.1 [2.7, 10.1]**	0.3 [0.0, 1.1]	0.3 [0.0, 1.1]	0.8 [0.0, 2.3]	0.4 [0.0, 1.5]		0.1 [0.0, 0.8]	0.3 [0.0, 1.2]	1.8 [0.1, 3.8]
SND	PDA	0.3 [0.0, 1.1]	0.3 [0.0, 1.6]	0.4 [0.0, 1.8]	0.3 [0.0, 1.3]	0.2 [0.0, 1.0]	0.4 [0.1, 1.7]	0.4 [0.0, 1.4]	0.3 [0.0, 1.2]	0.3 [0.0, 1.7]	0.4 [0.0, 1.4]	0.4 [0.0, 1.7]	0.3 [0.0, 1.6]	0.3 [0.0, 1.2]	0.3 [0.0, 1.6]	0.3 [0.0, 1.1]	0.3 [0.0, 1.9]	0.3 [0.0, 1.3]		0.3 [0.0, 1.3]	0.3 [0.0, 1.7]
	Stratified	0.3 [0.0, 1.3]	0.2 [0.0, 1.2]	0.5 [0.0, 1.2]	0.2 [0.0, 1.1]	0.3 [0.0, 1.5]	0.3 [0.0, 1.2]	0.3 [0.0, 1.1]	0.3 [0.0, 1.4]	0.5 [0.0, 2.0]	0.7 [0.0, 2.2]	0.4 [0.0, 1.5]	0.5 [0.0, 2.1]	0.3 [0.0, 0.8]	0.3 [0.0, 1.5]	0.3 [0.0, 1.1]	0.5 [0.0, 1.8]	0.3 [0.0, 1.4]		0.4 [0.0, 1.5]	0.3 [0.0, 1.1]
SNO	PDA	0.3 [0.0, 1.4]	0.5 [0.0, 2.1]	0.2 [0.0, 1.1]	0.7 [0.0, 2.1]	0.3 [0.0, 1.6]	0.3 [0.0, 1.5]	0.4 [0.0, 1.2]	0.2 [0.0, 1.0]	0.4 [0.0, 1.3]	0.2 [0.0, 0.9]	0.7 [0.0, 2.5]	0.4 [0.0, 1.4]	0.3 [0.0, 1.2]	0.4 [0.0, 1.4]	0.3 [0.0, 1.1]	0.2 [0.0, 1.3]	0.3 [0.0, 1.3]	0.5 [0.0, 1.7]		0.3 [0.0, 1.3]
	Stratified	0.4 [0.0, 1.6]	0.9 [0.0, 2.7]	0.4 [0.1, 1.3]	0.6 [0.0, 1.9]	0.3 [0.0, 1.2]	0.4 [0.0, 1.5]	0.3 [0.0, 1.2]	0.2 [0.0, 1.0]	0.4 [0.0, 1.9]	0.2 [0.0, 1.5]	1.0 [0.0, 3.0]	0.4 [0.0, 1.2]	0.2 [0.0, 1.1]	0.3 [0.0, 1.4]	0.3 [0.0, 1.3]	0.4 [0.0, 1.7]	0.4 [0.0, 2.0]	0.3 [0.0, 1.0]		0.3 [0.0, 1.2]
WIG	PDA	0.4 [0.0, 1.8]	0.4 [0.0, 1.3]	0.5 [0.0, 1.4]	0.5 [0.0, 2.0]	0.4 [0.0, 1.4]	0.7 [0.0, 2.3]	0.8 [0.0, 2.5]	0.4 [0.0, 1.7]	0.5 [0.0, 1.7]	0.2 [0.0, 1.1]	0.6 [0.0, 2.4]	0.5 [0.0, 1.6]	0.3 [0.0, 1.4]	0.3 [0.0, 1.1]	0.4 [0.0, 1.6]	0.4 [0.0, 1.2]	1.2 [0.0, 3.2]	0.3 [0.0, 1.4]	0.4 [0.0, 1.3]	
	Stratified	0.4 [0.0, 1.4]	0.5 [0.0, 1.8]	0.2 [0.0, 1.2]	0.5 [0.0, 1.6]	0.3 [0.0, 1.8]	0.6 [0.0, 1.9]	0.7 [0.0, 2.2]	0.4 [0.0, 1.8]	0.9 [0.0, 2.6]	0.3 [0.0, 1.2]	0.7 [0.0, 2.9]	0.4 [0.0, 1.5]	0.3 [0.0, 1.1]	0.3 [0.0, 1.1]	0.4 [0.0, 1.8]	0.4 [0.0, 1.4]	0.8 [0.0, 2.7]	0.3 [0.0, 1.6]	0.2 [0.0, 1.1]	

Table J. Geographic region transition rate matrix from combined internal gene model. Median rates and 95% highest posterior density intervals are displayed for both subsampling strategies. Rates colored in blue are statistically supported (Bayes factor > 100). (AK – Alaska, ALB – Alberta, BCO – British Columbia, GUA – Guatemala, MW – Midwest, NBR – New Brunswick, NE – Northeast, NFL – Newfoundland and Labrador, RP – Northern Rockies and Plains, NSC – Nova Scotia, NW – Northwest, OV – Ohio Valley, PEI – Prince Edward Island, QUE – Quebec, S – South, SE – Southeast, SON – Sonora, SW – Southwest, W – West)

Source	Sample	Sink																			
		AK	ALB	BCO	GUA	MW	NBR	NE	NFL	NSC	NW	ONT	OV	PEI	QUE	RP	S	SE	SON	SW	W
AK	PDA		**2.6 [1.3, 4.2]**	0.3 [0.0, 0.8]	0.2 [0.0, 0.7]	1.2 [0.1, 2.5]	0.1 [0.0, 0.4]	1.2 [0.1, 2.4]	0.2 [0.0, 0.6]	0.2 [0.0, 0.5]	**2.5 [1.3, 3.8]**	0.1 [0.0, 0.5]	0.7 [0.0, 1.7]	0.0 [0.0, 0.2]	0.2 [0.0, 0.6]	0.4 [0.0, 1.0]	**5.5 [3.1, 8.0]**	0.2 [0.0, 0.7]	0.1 [0.0, 0.4]	0.6 [0.1, 1.3]	**10.5 [7.5, 13.8]**
	Stratified		**2.1 [1.0, 3.5]**	0.5 [0.0, 1.1]	0.4 [0.0, 0.9]	0.7 [0.0, 1.9]	0.1 [0.0, 0.4]	0.9 [0.2, 2.0]	0.2 [0.0, 0.6]	0.1 [0.0, 0.2]	**3.3 [2.1, 4.8]**	0.1 [0.0, 0.4]	0.9 [0.0, 2.2]	0.1 [0.0, 0.4]	0.2 [0.0, 0.6]	0.4 [0.0, 0.9]	**3.3 [1.6, 5.2]**	0.3 [0.0, 0.7]	0.1 [0.0, 0.4]	0.6 [0.1, 1.4]	**8.8 [6.1, 11.7]**
ALB	PDA	1.3 [0.0, 3.4]		0.2 [0.0, 1.0]	0.1 [0.0, 0.7]	0.9 [0.0, 2.7]	0.3 [0.0, 1.1]	0.3 [0.0, 1.6]	0.3 [0.0, 0.9]	0.2 [0.0, 0.9]	0.4 [0.0, 1.2]	0.3 [0.0, 1.1]	0.3 [0.0, 1.5]	0.2 [0.0, 0.7]	0.2 [0.0, 0.8]	0.4 [0.0, 1.3]	0.6 [0.0, 2.3]	0.3 [0.0, 1.2]	0.3 [0.0, 1.2]	0.2 [0.0, 1.0]	0.8 [0.0, 2.5]
	Stratified	**3.3 [0.9, 6.4]**		**1.4 [0.3, 2.8]**	0.2 [0.0, 0.8]	1.8 [0.0, 4.4]	0.2 [0.0, 0.7]	0.5 [0.0, 1.7]	0.2 [0.0, 0.6]	0.2 [0.0, 0.5]	0.6 [0.0, 1.8]	0.2 [0.0, 0.5]	1.2 [0.0, 3.2]	0.1 [0.0, 0.6]	0.2 [0.0, 0.7]	1.0 [0.1, 2.3]	1.4 [0.1, 3.9]	0.5 [0.0, 1.4]	0.5 [0.0, 1.3]	0.3 [0.0, 1.0]	2.2 [0.1, 5.1]
BCO	PDA	0.3 [0.0, 0.9]	0.2 [0.0, 0.8]		0.3 [0.0, 0.9]	0.2 [0.0, 0.6]	0.2 [0.0, 0.9]	0.2 [0.0, 0.7]	0.2 [0.0, 0.8]	0.3 [0.0, 0.8]	0.2 [0.0, 1.0]	0.2 [0.0, 1.0]	0.2 [0.0, 1.1]	0.1 [0.0, 0.6]	0.2 [0.0, 1.1]	0.3 [0.0, 0.9]	0.3 [0.0, 0.9]	0.2 [0.0, 1.1]	0.2 [0.0, 1.0]	0.2 [0.0, 0.8]	0.3 [0.0, 1.1]
	Stratified	**1.4 [0.2, 3.0]**	0.5 [0.0, 1.6]		0.1 [0.0, 0.7]	0.3 [0.0, 0.9]	0.2 [0.0, 1.0]	0.3 [0.0, 0.9]	0.1 [0.0, 0.6]	0.2 [0.0, 0.8]	1.1 [0.1, 2.5]	0.2 [0.0, 1.0]	0.2 [0.0, 0.9]	0.2 [0.0, 0.6]	0.2 [0.0, 0.9]	0.2 [0.0, 0.8]	0.2 [0.0, 1.2]	0.1 [0.0, 0.9]	0.2 [0.0, 0.8]	0.2 [0.0, 0.7]	0.5 [0.0, 1.7]
GUA	PDA	0.5 [0.0, 2.0]	0.3 [0.0, 0.8]	0.2 [0.0, 0.9]		0.4 [0.0, 1.6]	0.3 [0.0, 1.0]	0.3 [0.0, 1.1]	0.3 [0.0, 0.9]	0.4 [0.0, 1.3]	0.2 [0.0, 1.1]	0.2 [0.0, 0.9]	0.4 [0.0, 1.5]	0.3 [0.0, 0.9]	0.1 [0.0, 0.8]	0.1 [0.0, 0.9]	0.5 [0.0, 1.8]	0.2 [0.0, 0.9]	0.3 [0.0, 1.0]	0.1 [0.0, 1.1]	0.8 [0.0, 2.4]
	Stratified	0.5 [0.0, 1.6]	0.2 [0.0, 1.3]	0.2 [0.0, 0.7]		0.4 [0.0, 1.9]	0.2 [0.0, 0.9]	0.3 [0.0, 1.4]	0.2 [0.0, 0.8]	0.4 [0.0, 1.3]	0.2 [0.0, 1.1]	0.2 [0.0, 0.8]	1.8 [0.2, 4.0]	0.1 [0.0, 0.6]	0.2 [0.0, 0.8]	0.1 [0.0, 0.7]	0.5 [0.0, 1.8]	0.2 [0.0, 0.9]	0.2 [0.0, 0.9]	0.3 [0.0, 1.1]	1.5 [0.1, 3.3]
MW	PDA	3.0 [0.9, 5.6]	2.1 [0.4, 3.9]	0.1 [0.0, 0.6]	**1.9 [0.6, 3.3]**		**5.7 [3.5, 8.2]**	**20.5 [14.9, 26.4]**	0.9 [0.0, 1.8]	**1.9 [0.8, 3.1]**	0.6 [0.0, 1.5]	0.3 [0.0, 0.9]	**24.0 [18.8, 29.7]**	0.2 [0.0, 0.8]	0.2 [0.0, 0.7]	**3.1 [1.6, 4.7]**	**24.1 [16.0, 32.2]**	0.6 [0.0, 1.4]	0.4 [0.0, 1.2]	0.5 [0.0, 1.2]	**6.5 [2.7, 10.9]**
	Stratified	**4.6 [2.6, 7.0]**	2.4 [0.6, 4.6]	0.2 [0.0, 0.7]	1.7 [0.3, 3.2]		**3.6 [1.7, 5.5]**	**10.6 [7.1, 14.6]**	0.6 [0.0, 1.5]	0.8 [0.1, 1.7]	0.9 [0.0, 2.0]	0.3 [0.0, 0.8]	**28.0 [21.9, 34.3]**	0.8 [0.2, 1.7]	0.2 [0.0, 0.4]	**3.1 [1.4, 4.8]**	**22.7 [17.3, 28.4]**	0.8 [0.1, 1.7]	0.8 [0.1, 1.7]	1.2 [0.2, 2.3]	**7.1 [3.5, 11.2]**
NBR	PDA	0.3 [0.0, 1.1]	0.2 [0.0, 0.8]	0.2 [0.0, 0.7]	0.3 [0.0, 1.0]	0.4 [0.0, 1.6]		0.8 [0.0, 2.4]	0.7 [0.0, 1.7]	0.6 [0.0, 1.8]	0.2 [0.0, 0.9]	0.2 [0.0, 0.8]	0.4 [0.0, 1.7]	0.9 [0.1, 2.1]	0.4 [0.0, 1.3]	0.2 [0.0, 0.8]	0.3 [0.0, 1.3]	0.2 [0.0, 1.0]	0.2 [0.0, 1.0]	0.2 [0.0, 0.8]	0.3 [0.0, 1.6]
	Stratified	0.3 [0.0, 1.0]	0.2 [0.0, 0.8]	**0.6 [0.1, 1.5]**	0.2 [0.0, 0.8]	0.6 [0.0, 2.1]		**3.4 [1.0, 6.5]**	2.0 [0.4, 4.0]	**12.4 [8.1, 16.8]**	0.2 [0.0, 0.8]	0.4 [0.0, 1.4]	1.3 [0.2, 2.8]	**5.6 [3.2, 8.5]**	1.0 [0.1, 2.3]	0.2 [0.0, 1.0]	0.5 [0.0, 1.6]	0.5 [0.0, 1.5]	0.2 [0.0, 0.8]	0.2 [0.0, 1.0]	0.2 [0.0, 0.8]
NE	PDA	**3.3 [1.7, 5.2]**	0.6 [0.0, 1.5]	0.1 [0.0, 0.4]	0.2 [0.0, 0.5]	**10.7 [7.2, 14.3]**	**11.3 [8.8, 14.1]**		**4.3 [2.9, 6.0]**	**3.5 [2.2, 5.0]**	0.2 [0.0, 0.7]	**2.1 [1.2, 3.1]**	**10.2 [7.2, 13.5]**	**1.6 [0.8, 2.6]**	**3.8 [2.5, 5.2]**	0.2 [0.0, 0.8]	**2.7 [0.8, 5.0]**	**1.8 [0.9, 2.9]**	0.2 [0.0, 0.6]	0.2 [0.0, 0.6]	**2.2 [0.6, 4.1]**
	Stratified	**2.5 [1.1, 4.2]**	0.4 [0.0, 1.1]	0.2 [0.0, 0.6]	0.1 [0.0, 0.4]	**7.5 [4.2, 10.9]**	**5.8 [2.8, 8.9]**		**3.2 [1.5, 5.0]**	**3.3 [1.4, 5.3]**	0.2 [0.0, 0.7]	**1.2 [0.3, 2.3]**	**3.3 [0.7, 7.0]**	**1.7 [0.5, 3.1]**	**4.6 [2.9, 6.8]**	0.3 [0.0, 0.9]	1.0 [0.0, 2.4]	1.3 [0.2, 2.7]	0.1 [0.0, 0.5]	0.2 [0.0, 0.7]	0.8 [0.0, 1.9]
NFL	PDA	0.3 [0.0, 1.0]	0.2 [0.0, 0.7]	0.2 [0.0, 0.6]	0.2 [0.0, 0.6]	0.6 [0.0, 1.8]	0.6 [0.0, 1.8]	0.3 [0.0, 1.3]		0.4 [0.0, 1.5]	0.2 [0.0, 0.9]	0.2 [0.0, 0.6]	0.3 [0.0, 1.1]	0.2 [0.0, 0.8]	0.2 [0.0, 0.9]	0.2 [0.0, 0.6]	0.3 [0.0, 1.1]	0.2 [0.0, 1.3]	0.3 [0.0, 1.3]	0.2 [0.0, 0.8]	0.5 [0.0, 1.5]
	Stratified	0.5 [0.0, 1.2]	0.2 [0.0, 1.0]	0.2 [0.0, 0.8]	0.4 [0.0, 1.3]	0.6 [0.0, 1.7]	0.8 [0.0, 2.1]	0.4 [0.0, 1.6]		0.5 [0.0, 1.7]	0.2 [0.0, 0.8]	0.2 [0.0, 0.7]	0.4 [0.0, 1.6]	0.5 [0.0, 1.3]	0.3 [0.0, 1.3]	0.1 [0.0, 0.6]	0.5 [0.0, 1.8]	0.2 [0.0, 0.8]	0.2 [0.0, 0.5]	0.2 [0.0, 0.8]	0.5 [0.0, 1.6]
NSC	PDA	0.3 [0.0, 1.0]	0.3 [0.0, 1.0]	0.2 [0.0, 0.8]	0.3 [0.0, 0.9]	0.3 [0.0, 1.5]	0.5 [0.0, 1.6]	0.4 [0.0, 1.2]	0.2 [0.0, 1.0]		0.2 [0.0, 1.2]	0.2 [0.0, 1.0]	0.2 [0.0, 1.2]	0.5 [0.0, 1.2]	0.3 [0.0, 0.8]	0.2 [0.0, 0.8]	0.2 [0.0, 1.0]	0.4 [0.0, 1.8]	0.2 [0.0, 0.6]	0.2 [0.0, 0.7]	0.3 [0.0, 1.3]
	Stratified	0.3 [0.0, 1.1]	0.2 [0.0, 0.8]	0.2 [0.0, 1.3]	0.3 [0.0, 1.1]	0.3 [0.0, 1.1]	1.7 [0.3, 3.7]	0.7 [0.0, 2.1]	0.5 [0.0, 1.6]		0.2 [0.0, 0.8]	0.1 [0.0, 0.8]	0.5 [0.0, 1.5]	**3.7 [1.7, 6.3]**	0.4 [0.0, 1.2]	0.3 [0.0, 1.1]	0.3 [0.0, 1.1]	0.3 [0.0, 0.9]	0.1 [0.0, 0.6]	0.2 [0.0, 0.7]	0.4 [0.0, 1.4]
NW	PDA	0.4 [0.0, 1.6]	0.3 [0.0, 1.3]	0.3 [0.0, 1.0]	0.2 [0.0, 0.8]	0.3 [0.0, 1.3]	0.3 [0.0, 1.2]	0.3 [0.0, 1.2]	0.2 [0.0, 0.9]	0.2 [0.0, 0.8]		0.3 [0.0, 1.1]	0.3 [0.0, 1.0]	0.2 [0.0, 0.8]	0.2 [0.0, 1.0]	0.2 [0.0, 1.1]	0.2 [0.0, 1.2]	0.1 [0.0, 1.3]	0.2 [0.0, 0.8]	0.4 [0.0, 1.4]	0.6 [0.0, 1.7]
	Stratified	2.1 [0.2, 4.5]	0.7 [0.0, 2.0]	0.8 [0.0, 2.2]	0.2 [0.0, 0.8]	1.3 [0.0, 3.0]	0.1 [0.0, 0.6]	0.4 [0.0, 1.5]	0.2 [0.0, 0.9]	0.3 [0.0, 1.1]		0.2 [0.0, 0.8]	1.1 [0.0, 2.7]	0.3 [0.0, 1.0]	0.2 [0.0, 0.9]	0.5 [0.0, 1.6]	1.2 [0.1, 3.2]	0.2 [0.0, 0.8]	0.3 [0.0, 0.9]	1.0 [0.1, 2.4]	2.0 [0.2, 4.7]
ONT	PDA	0.3 [0.0, 1.3]	0.2 [0.0, 0.9]	0.2 [0.0, 0.9]	0.2 [0.0, 0.9]	0.3 [0.0, 1.0]	0.3 [0.0, 0.8]	0.2 [0.0, 1.2]	0.1 [0.0, 1.0]	0.1 [0.0, 0.7]	0.2 [0.0, 0.8]		0.3 [0.0, 1.3]	0.2 [0.0, 0.8]	0.2 [0.0, 0.7]	0.2 [0.0, 0.6]	0.2 [0.0, 0.8]	0.2 [0.0, 0.7]	0.2 [0.0, 0.6]	0.2 [0.0, 0.9]	0.3 [0.0, 1.1]
	Stratified	0.3 [0.0, 0.9]	0.2 [0.0, 1.1]	0.2 [0.0, 0.8]	0.3 [0.0, 1.0]	0.2 [0.0, 0.8]	0.3 [0.0, 1.0]	0.2 [0.0, 1.0]	0.2 [0.0, 0.9]	0.2 [0.0, 0.6]	0.2 [0.0, 0.9]		0.2 [0.0, 0.9]	0.3 [0.0, 1.2]	0.3 [0.0, 1.2]	0.2 [0.0, 0.9]	0.2 [0.0, 1.2]	0.5 [0.0, 1.7]	0.2 [0.0, 0.6]	0.2 [0.0, 0.9]	0.4 [0.0, 1.5]
OV	PDA	1.0 [0.0, 2.8]	**3.0 [1.2, 5.1]**	0.1 [0.0, 0.4]	0.7 [0.0, 1.9]	**9.1 [5.5, 13.1]**	0.7 [0.0, 1.9]	**18.9 [13.9, 23.8]**	0.2 [0.0, 1.0]	0.2 [0.0, 0.6]	**2.4 [0.9, 4.1]**	0.3 [0.0, 1.0]		0.1 [0.0, 0.3]	0.1 [0.0, 0.5]	1.0 [0.1, 2.0]	**10.0 [5.6, 14.8]**	0.5 [0.0, 1.2]	0.1 [0.0, 0.5]	0.8 [0.0, 1.8]	3.4 [0.5, 6.6]
	Stratified	1.0 [0.0, 2.5]	**2.8 [1.2, 4.7]**	0.1 [0.0, 0.5]	1.1 [0.1, 2.5]	**21.0 [15.6, 26.5]**	**3.0 [0.9, 5.3]**	**26.3 [21.1, 32.0]**	1.1 [0.3, 2.0]	0.9 [0.1, 1.9]	1.3 [0.3, 2.6]	**2.0 [1.0, 3.0]**		0.5 [0.0, 1.2]	1.2 [0.3, 2.3]	**2.1 [0.9, 3.6]**	**7.7 [4.4, 11.4]**	**2.6 [1.0, 4.3]**	0.3 [0.0, 0.8]	0.9 [0.1, 1.9]	**7.1 [4.0, 10.6]**
PEI	PDA	0.2 [0.0, 0.9]	0.3 [0.0, 1.0]	0.2 [0.0, 0.9]	0.2 [0.0, 0.6]	0.2 [0.0, 1.0]	0.2 [0.0, 1.1]	0.2 [0.0, 0.8]	0.3 [0.0, 1.1]	0.3 [0.0, 1.0]	0.2 [0.0, 0.8]	0.2 [0.0, 0.9]	0.2 [0.0, 0.8]		0.3 [0.0, 1.0]	0.2 [0.0, 0.9]	0.2 [0.0, 1.2]	0.3 [0.0, 1.5]	0.3 [0.0, 0.9]	0.2 [0.0, 0.7]	0.3 [0.0, 1.4]
	Stratified	0.3 [0.0, 1.2]	0.2 [0.0, 1.0]	0.2 [0.0, 1.0]	0.2 [0.0, 0.9]	0.3 [0.0, 1.2]	**2.4 [0.9, 4.3]**	0.5 [0.0, 1.6]	0.5 [0.0, 1.4]	**2.6 [0.8, 4.7]**	0.1 [0.0, 0.7]	0.3 [0.0, 0.9]	0.3 [0.0, 1.1]		0.2 [0.0, 0.8]	0.1 [0.0, 0.8]	0.3 [0.0, 1.1]	0.3 [0.0, 1.0]	0.2 [0.0, 0.6]	0.2 [0.0, 0.9]	0.2 [0.0, 1.2]
QUE	PDA	0.4 [0.0, 1.3]	0.2 [0.0, 0.8]	0.3 [0.0, 1.0]	0.2 [0.0, 0.8]	0.3 [0.0, 1.2]	0.5 [0.0, 1.6]	0.4 [0.0, 1.3]	0.3 [0.0, 0.9]	0.2 [0.0, 0.8]	0.2 [0.0, 0.9]	0.2 [0.0, 0.7]	0.3 [0.0, 0.9]	0.2 [0.0, 0.8]		0.2 [0.0, 0.8]	0.3 [0.0, 1.1]	0.2 [0.0, 0.6]	0.2 [0.0, 0.8]	0.2 [0.0, 0.7]	0.2 [0.0, 0.9]
	Stratified	0.6 [0.0, 2.0]	0.2 [0.0, 0.9]	0.2 [0.0, 0.6]	0.2 [0.0, 0.9]	0.3 [0.0, 1.2]	**2.4 [0.9, 4.4]**	1.3 [0.0, 3.1]	0.4 [0.0, 1.3]	0.4 [0.0, 1.4]	0.2 [0.0, 0.9]	0.5 [0.0, 1.4]	0.6 [0.0, 2.0]	0.5 [0.0, 1.4]		0.2 [0.0, 0.6]	0.3 [0.0, 0.8]	0.2 [0.0, 0.9]	0.2 [0.0, 1.0]	0.2 [0.0, 0.8]	0.4 [0.0, 1.4]
RP	PDA	0.6 [0.0, 1.8]	0.4 [0.0, 1.1]	0.4 [0.0, 1.3]	0.2 [0.0, 0.7]	0.2 [0.0, 0.8]	0.2 [0.0, 1.1]	0.2 [0.0, 0.9]	0.3 [0.0, 0.9]	0.2 [0.0, 1.0]	0.2 [0.0, 0.8]	0.2 [0.0, 0.9]	0.3 [0.0, 1.2]	0.2 [0.0, 0.8]	0.3 [0.0, 1.0]		0.2 [0.0, 1.2]	0.3 [0.0, 1.4]	0.2 [0.0, 0.7]	0.3 [0.0, 1.1]	0.4 [0.0, 1.3]
	Stratified	1.4 [0.1, 3.2]	0.7 [0.0, 2.1]	0.2 [0.0, 0.7]	0.3 [0.0, 0.9]	0.8 [0.0, 2.1]	0.2 [0.0, 0.8]	0.4 [0.0, 1.2]	0.2 [0.0, 1.0]	0.2 [0.0, 0.8]	0.3 [0.0, 1.4]	0.2 [0.0, 0.6]	1.0 [0.1, 2.6]	0.3 [0.0, 1.0]	0.2 [0.0, 1.2]		0.6 [0.0, 2.0]	0.2 [0.0, 1.0]	0.3 [0.0, 1.0]	0.3 [0.0, 1.1]	0.8 [0.0, 2.4]
S	PDA	**8.8 [5.8, 11.8]**	**6.0 [3.6, 8.3]**	0.1 [0.0, 0.4]	**11.7 [9.3, 14.2]**	**28.7 [22.0, 35.2]**	0.9 [0.1, 1.9]	**12.6 [8.4, 16.8]**	0.5 [0.0, 1.0]	0.5 [0.0, 1.0]	**2.6 [1.2, 4.2]**	0.2 [0.0, 0.6]	**48.5 [42.5, 55.0]**	0.1 [0.0, 0.3]	0.1 [0.0, 0.5]	**2.3 [1.0, 3.7]**		0.1 [0.0, 0.4]	**1.2 [0.5, 2.1]**	**3.3 [1.9, 4.8]**	**28.4 [23.1, 33.5]**
	Stratified	**5.2 [2.6, 7.7]**	**7.7 [5.0, 10.6]**	0.2 [0.0, 0.6]	**6.1 [4.0, 8.7]**	**15.9 [10.2, 21.6]**	0.3 [0.0, 1.2]	**6.4 [3.6, 9.5]**	0.5 [0.0, 1.4]	**1.0 [0.3, 1.9]**	**3.5 [1.6, 5.5]**	0.1 [0.0, 0.4]	**33.0 [27.1, 39.5]**	0.2 [0.0, 0.7]	0.3 [0.0, 0.8]	**4.4 [2.6, 6.5]**		0.4 [0.0, 0.9]	1.1 [0.1, 2.2]	**2.8 [1.2, 4.6]**	**21.7 [16.8, 27.2]**
SE	PDA	0.2 [0.0, 1.0]	0.2 [0.0, 0.8]	0.2 [0.0, 0.9]	0.2 [0.0, 0.6]	0.3 [0.0, 1.2]	0.2 [0.0, 0.8]	0.2 [0.0, 0.9]	0.2 [0.0, 0.5]	0.1 [0.0, 0.8]	0.2 [0.0, 0.8]	0.2 [0.0, 0.8]	0.2 [0.0, 0.9]	0.2 [0.0, 0.9]	0.3 [0.0, 1.2]	0.1 [0.0, 0.7]	0.2 [0.0, 0.8]		0.2 [0.0, 1.0]	0.2 [0.0, 0.9]	0.3 [0.0, 1.1]
	Stratified	0.2 [0.0, 1.2]	0.3 [0.0, 1.0]	0.2 [0.0, 0.7]	0.3 [0.0, 1.0]	0.4 [0.0, 1.5]	0.5 [0.0, 1.6]	0.5 [0.0, 1.5]	0.1 [0.0, 0.7]	0.4 [0.0, 1.3]	0.2 [0.0, 0.8]	0.3 [0.0, 1.1]	0.5 [0.0, 1.7]	0.4 [0.0, 1.3]	0.1 [0.0, 0.8]	0.1 [0.0, 0.6]	0.3 [0.0, 1.3]		0.2 [0.0, 0.7]	0.3 [0.0, 1.1]	0.6 [0.0, 1.9]
SON	PDA	0.3 [0.0, 0.8]	0.2 [0.0, 0.9]	0.2 [0.0, 0.8]	0.2 [0.0, 0.9]	0.3 [0.0, 1.0]	0.2 [0.0, 0.9]	0.2 [0.0, 0.8]	0.2 [0.0, 0.8]	0.2 [0.0, 0.7]	0.2 [0.0, 1.1]	0.3 [0.0, 0.8]	0.3 [0.0, 1.0]	0.3 [0.0, 0.7]	0.3 [0.0, 0.6]	0.2 [0.0, 0.8]	0.2 [0.0, 0.8]	0.2 [0.0, 0.7]		0.4 [0.0, 1.3]	0.3 [0.0, 1.1]
	Stratified	0.3 [0.0, 0.9]	0.1 [0.0, 1.0]	0.2 [0.0, 0.6]	0.3 [0.0, 1.3]	0.3 [0.0, 1.1]	0.2 [0.0, 0.9]	0.3 [0.0, 0.9]	0.2 [0.0, 0.8]	0.2 [0.0, 0.8]	0.3 [0.0, 1.2]	0.2 [0.0, 0.8]	0.2 [0.0, 1.0]	0.2 [0.0, 1.0]	0.2 [0.0, 1.1]	0.2 [0.0, 1.0]	0.4 [0.0, 1.3]	0.2 [0.0, 0.9]		0.4 [0.0, 1.1]	0.4 [0.0, 1.4]
SW	PDA	0.3 [0.0, 1.2]	0.2 [0.0, 1.1]	0.2 [0.0, 0.9]	0.2 [0.0, 1.0]	0.2 [0.0, 1.0]	0.2 [0.0, 0.9]	0.3 [0.0, 0.9]	0.2 [0.0, 1.1]	0.3 [0.0, 1.1]	0.3 [0.0, 1.2]	0.2 [0.0, 0.8]	0.3 [0.0, 1.1]	0.2 [0.0, 0.7]	0.3 [0.0, 1.1]	0.3 [0.0, 0.9]	0.3 [0.0, 1.3]	0.2 [0.0, 0.7]	0.2 [0.0, 1.0]		0.3 [0.0, 1.4]
	Stratified	0.3 [0.0, 1.4]	0.3 [0.0, 1.0]	0.3 [0.0, 0.9]	0.3 [0.0, 0.9]	0.5 [0.0, 1.8]	0.2 [0.0, 0.7]	0.3 [0.0, 1.1]	0.2 [0.0, 1.0]	0.3 [0.0, 0.9]	0.6 [0.0, 1.7]	0.2 [0.0, 0.8]	0.4 [0.0, 1.9]	0.2 [0.0, 0.6]	0.2 [0.0, 0.8]	0.6 [0.0, 1.7]	0.5 [0.0, 1.8]	0.2 [0.0, 1.1]	0.7 [0.1, 1.7]		**2.7 [0.8, 5.2]**
W	PDA	**30.7 [26.1, 35.3]**	**11.6 [9.0, 14.4]**	**1.1 [0.5, 1.8]**	1.0 [0.2, 1.9]	**17.7 [13.0, 22.2]**	0.4 [0.0, 1.0]	**7.5 [4.6, 10.5]**	0.3 [0.0, 0.9]	0.1 [0.0, 0.4]	**8.3 [6.2, 10.4]**	0.3 [0.0, 0.7]	**6.8 [4.3, 9.8]**	0.1 [0.0, 0.5]	0.7 [0.1, 1.3]	**3.4 [2.1, 4.8]**	**18.3 [13.8, 23.2]**	0.6 [0.1, 1.2]	**1.1 [0.4, 2.0]**	**4.4 [2.8, 6.0]**	
	Stratified	**17.3 [14.0, 20.7]**	**8.6 [6.3, 11.0]**	0.6 [0.1, 1.2]	**1.4 [0.5, 2.6]**	**12.2 [8.6, 15.9]**	0.2 [0.0, 0.7]	2.2 [0.5, 4.1]	0.3 [0.0, 0.8]	0.1 [0.0, 0.5]	**8.8 [6.7, 11.0]**	0.2 [0.0, 0.4]	**12.1 [8.5, 16.1]**	0.1 [0.0, 0.4]	**1.2 [0.5, 2.1]**	**2.9 [1.6, 4.2]**	**10.6 [7.5, 14.0]**	0.4 [0.0, 1.0]	**1.9 [1.0, 2.9]**	**7.1 [5.1, 9.1]**	

Table K. Geographic region transition rate matrix from combined hemagglutinin subtype model. Median rates and 95% highest posterior density intervals are displayed for both subsampling strategies. Rates colored in blue are statistically supported (Bayes factor > 100). (AK – Alaska, ALB – Alberta, BCO – British Columbia, GUA – Guatemala, MW – Midwest, NBR – New Brunswick, NE – Northeast, NFL – Newfoundland and Labrador, RP – Northern Rockies and Plains, NSC – Nova Scotia, NW – Northwest, OV – Ohio Valley, PEI – Prince Edward Island, QUE – Quebec, S – South, SE – Southeast, SON – Sonora, SW – Southwest, W – West)

Source	Sample	Sink													
		AK	ALB	GUA	MW	NBR	NE	NSC	NW	OV	QUE	RP	S	SW	W
AK	PDA		**2.3 [1.0, 4.0]**	0.1 [0.0, 0.5]	0.8 [0.0, 2.0]	0.2 [0.0, 0.7]	0.2 [0.0, 0.9]	0.1 [0.0, 0.5]	**1.3 [0.3, 2.6]**	0.7 [0.0, 1.8]	0.1 [0.0, 0.6]	0.2 [0.0, 0.8]	0.6 [0.0, 1.8]	0.3 [0.0, 0.9]	**3.4 [1.3, 5.7]**
	Stratified		**2.3 [0.8, 4.0]**	0.2 [0.0, 0.7]	0.8 [0.0, 2.2]	0.2 [0.0, 0.9]	0.5 [0.0, 1.4]	0.2 [0.0, 0.7]	**1.9 [0.6, 3.4]**	0.8 [0.0, 2.2]	0.1 [0.0, 0.6]	0.2 [0.0, 0.7]	0.5 [0.0, 1.5]	0.2 [0.0, 0.9]	**3.6 [1.5, 6.1]**
ALB	PDA	1.8 [0.0, 4.4]		0.2 [0.0, 0.9]	3.0 [0.0, 6.7]	0.2 [0.0, 1.1]	0.8 [0.0, 2.3]	0.3 [0.0, 1.1]	0.5 [0.0, 1.6]	0.8 [0.0, 2.6]	0.2 [0.0, 1.0]	0.8 [0.0, 2.0]	1.1 [0.0, 3.1]	0.7 [0.0, 2.1]	**2.9 [0.3, 6.7]**
	Stratified	1.9 [0.0, 4.7]		0.2 [0.0, 0.9]	1.8 [0.0, 4.9]	0.3 [0.0, 1.3]	1.2 [0.0, 2.9]	0.2 [0.0, 1.1]	0.8 [0.0, 2.3]	0.8 [0.0, 2.5]	0.2 [0.0, 1.0]	1.0 [0.1, 2.3]	0.7 [0.0, 2.2]	0.5 [0.0, 1.5]	**2.9 [0.4, 6.7]**
GUA	PDA	0.4 [0.0, 1.6]	0.3 [0.0, 1.3]		0.7 [0.0, 2.5]	0.3 [0.0, 1.1]	0.3 [0.0, 1.4]	0.3 [0.0, 1.2]	0.3 [0.0, 1.1]	0.4 [0.0, 1.5]	0.3 [0.0, 1.1]	0.3 [0.0, 1.2]	0.9 [0.0, 3.0]	0.3 [0.0, 1.3]	0.7 [0.0, 2.2]
	Stratified	0.3 [0.0, 1.3]	0.3 [0.0, 1.2]		0.3 [0.0, 1.5]	0.3 [0.0, 1.1]	0.3 [0.0, 1.4]	0.3 [0.0, 1.1]	0.3 [0.0, 1.3]	0.3 [0.0, 1.3]	0.3 [0.0, 1.2]	0.3 [0.0, 1.2]	0.4 [0.0, 1.5]	0.2 [0.0, 1.0]	0.8 [0.0, 2.6]
MW	PDA	**3.0 [0.8, 5.5]**	**4.4 [1.3, 7.7]**	0.8 [0.0, 2.0]		**3.7 [1.8, 5.8]**	**9.1 [5.5, 12.8]**	0.8 [0.0, 1.9]	1.2 [0.2, 2.5]	**13.0 [8.6, 17.7]**	0.2 [0.0, 1.1]	**5.1 [3.2, 7.3]**	**13.0 [9.2, 17.2]**	**1.9 [0.7, 3.4]**	**5.2 [2.2, 8.7]**
	Stratified	1.8 [0.2, 4.0]	**1.8 [0.2, 3.6]**	1.0 [0.0, 2.2]		**3.1 [1.2, 5.3]**	**9.5 [6.1, 13.7]**	0.7 [0.0, 2.1]	0.7 [0.0, 2.2]	**17.8 [12.7, 23.4]**	0.5 [0.0, 1.4]	**4.5 [2.5, 6.8]**	**10.0 [6.3, 14.1]**	**1.9 [0.6, 3.4]**	**3.5 [0.7, 7.0]**
NBR	PDA	0.3 [0.0, 1.2]	0.3 [0.0, 1.1]	0.3 [0.0, 1.1]	0.5 [0.0, 1.8]		**2.0 [0.3, 4.4]**	**2.5 [0.8, 4.6]**	0.3 [0.0, 1.1]	0.6 [0.0, 2.1]	0.5 [0.0, 1.6]	0.3 [0.0, 1.0]	0.3 [0.0, 1.3]	0.3 [0.0, 1.1]	0.3 [0.0, 1.2]
	Stratified	0.4 [0.0, 1.5]	0.3 [0.0, 1.1]	0.3 [0.0, 1.2]	0.4 [0.0, 1.7]		1.3 [0.0, 3.4]	**4.8 [1.9, 8.6]**	0.5 [0.0, 1.5]	0.6 [0.0, 2.0]	0.3 [0.0, 1.3]	0.2 [0.0, 1.2]	0.3 [0.0, 1.3]	0.2 [0.0, 1.0]	0.3 [0.0, 1.4]
NE	PDA	0.2 [0.0, 0.9]	0.2 [0.0, 1.0]	0.2 [0.0, 0.7]	2.2 [0.2, 4.5]	1.7 [0.1, 3.5]		0.7 [0.0, 1.8]	0.2 [0.0, 0.8]	**3.3 [1.0, 5.9]**	**1.6 [0.3, 3.1]**	0.2 [0.0, 0.9]	0.3 [0.0, 1.3]	0.2 [0.0, 0.8]	0.7 [0.0, 2.0]
	Stratified	0.3 [0.0, 1.3]	0.4 [0.0, 1.5]	0.2 [0.0, 0.7]	**3.1 [1.1, 5.6]**	**2.1 [0.5, 4.1]**		1.0 [0.1, 2.2]	0.2 [0.0, 1.0]	**2.3 [0.6, 4.6]**	0.9 [0.1, 2.1]	0.2 [0.0, 1.1]	0.2 [0.0, 1.1]	0.2 [0.0, 0.7]	0.9 [0.0, 2.5]
NSC	PDA	0.3 [0.0, 1.1]	0.2 [0.0, 1.3]	0.2 [0.0, 1.0]	0.3 [0.0, 1.4]	0.3 [0.0, 1.3]	0.3 [0.0, 1.5]		0.3 [0.0, 1.1]	0.3 [0.0, 1.3]	0.5 [0.0, 1.7]	0.3 [0.0, 1.3]	0.4 [0.0, 1.5]	0.3 [0.0, 1.2]	0.3 [0.0, 1.3]
	Stratified	0.3 [0.0, 1.2]	0.3 [0.0, 1.1]	0.3 [0.0, 1.1]	0.4 [0.0, 1.4]	2.1 [0.2, 4.6]	0.6 [0.0, 2.0]		0.3 [0.0, 1.2]	0.5 [0.0, 1.6]	0.3 [0.0, 1.2]	0.3 [0.0, 1.0]	0.3 [0.0, 1.3]	0.2 [0.0, 1.0]	0.4 [0.0, 1.2]
NW	PDA	0.5 [0.0, 1.9]	0.3 [0.0, 1.3]	0.3 [0.0, 1.3]	0.3 [0.0, 1.3]	0.3 [0.0, 1.1]	0.2 [0.0, 1.1]	0.3 [0.0, 1.0]		0.2 [0.0, 1.2]	0.2 [0.0, 1.3]	0.6 [0.0, 1.8]	0.4 [0.0, 1.7]	0.6 [0.0, 1.8]	**1.9 [0.3, 4.0]**
	Stratified	0.9 [0.0, 2.6]	0.4 [0.0, 1.5]	0.3 [0.0, 1.1]	0.3 [0.0, 1.6]	0.3 [0.0, 1.3]	0.3 [0.0, 1.3]	0.3 [0.0, 1.1]		0.5 [0.0, 1.8]	0.2 [0.0, 1.2]	0.3 [0.0, 1.1]	0.4 [0.0, 1.7]	0.9 [0.0, 2.7]	**1.5 [0.1, 3.5]**
OV	PDA	1.4 [0.0, 3.0]	1.7 [0.3, 3.5]	0.7 [0.0, 1.7]	**12.8 [8.4, 17.8]**	1.1 [0.0, 2.6]	**7.7 [4.4, 11.5]**	0.9 [0.1, 2.0]	0.9 [0.1, 2.2]		0.7 [0.0, 1.8]	0.5 [0.0, 1.5]	**7.2 [3.2, 11.6]**	0.6 [0.0, 1.4]	2.3 [0.1, 5.0]
	Stratified	1.6 [0.1, 3.3]	**2.4 [0.8, 4.2]**	0.5 [0.0, 1.4]	**11.9 [7.8, 16.8]**	0.9 [0.0, 2.2]	**8.0 [5.0, 11.6]**	1.0 [0.2, 2.1]	**1.5 [0.4, 2.9]**		0.4 [0.0, 1.2]	0.6 [0.0, 1.6]	**9.5 [5.9, 13.3]**	0.5 [0.0, 1.6]	**3.5 [1.2, 6.4]**
QUE	PDA	0.3 [0.0, 1.2]	0.3 [0.0, 1.4]	0.3 [0.0, 1.1]	0.7 [0.0, 2.0]	0.3 [0.0, 1.1]	0.9 [0.0, 2.4]	0.3 [0.0, 1.5]	0.3 [0.0, 1.2]	0.5 [0.0, 1.8]		0.3 [0.0, 1.1]	0.6 [0.0, 1.9]	0.2 [0.0, 1.1]	0.6 [0.0, 2.0]
	Stratified	0.3 [0.0, 1.4]	0.3 [0.0, 1.4]	0.3 [0.0, 1.1]	0.7 [0.0, 2.2]	0.3 [0.0, 1.3]	0.9 [0.0, 2.5]	0.3 [0.0, 1.2]	0.3 [0.0, 1.1]	0.5 [0.0, 1.7]		0.4 [0.0, 1.5]	0.4 [0.0, 1.8]	0.2 [0.0, 1.1]	0.8 [0.0, 2.3]
RP	PDA	0.3 [0.0, 1.3]	0.4 [0.0, 1.4]	0.2 [0.0, 1.1]	0.4 [0.0, 1.6]	0.3 [0.0, 1.3]	0.3 [0.0, 1.3]	0.3 [0.0, 1.2]	0.3 [0.0, 1.2]	0.4 [0.0, 1.6]	0.3 [0.0, 1.4]		0.3 [0.0, 1.4]	0.3 [0.0, 1.2]	0.3 [0.0, 1.3]
	Stratified	0.3 [0.0, 1.5]	0.6 [0.0, 2.0]	0.3 [0.0, 1.2]	0.3 [0.0, 1.2]	0.4 [0.0, 1.4]	0.4 [0.0, 1.5]	0.3 [0.0, 1.2]	0.2 [0.0, 1.3]	0.3 [0.0, 1.3]	0.6 [0.0, 2.0]		0.3 [0.0, 1.2]	0.3 [0.0, 1.3]	0.3 [0.0, 1.3]
S	PDA	1.7 [0.1, 3.4]	0.5 [0.0, 1.8]	**5.7 [3.3, 8.2]**	**8.6 [5.0, 12.4]**	0.5 [0.0, 1.3]	**2.3 [0.6, 4.4]**	0.4 [0.0, 1.2]	0.8 [0.0, 2.0]	**17.5 [11.5, 23.2]**	0.8 [0.0, 1.9]	1.0 [0.1, 2.2]		0.8 [0.0, 2.0]	**6.1 [3.1, 9.8]**
	Stratified	1.5 [0.1, 3.3]	0.5 [0.0, 1.6]	**4.8 [2.6, 7.5]**	**5.6 [2.7, 9.3]**	0.2 [0.0, 0.9]	1.9 [0.2, 4.0]	0.2 [0.0, 1.0]	0.7 [0.0, 1.9]	**8.1 [4.5, 12.4]**	0.7 [0.0, 1.8]	0.8 [0.0, 2.2]		0.5 [0.0, 1.8]	**6.3 [2.8, 10.1]**
SW	PDA	0.3 [0.0, 1.2]	0.3 [0.0, 1.3]	0.3 [0.0, 1.2]	0.4 [0.0, 1.8]	0.3 [0.0, 1.0]	0.3 [0.0, 1.2]	0.3 [0.0, 1.1]	0.3 [0.0, 1.5]	0.3 [0.0, 1.4]	0.3 [0.0, 1.3]	0.6 [0.0, 1.9]	0.5 [0.0, 1.8]		**1.7 [0.2, 3.7]**
	Stratified	0.4 [0.0, 1.6]	0.3 [0.0, 1.1]	0.3 [0.0, 1.4]	0.4 [0.0, 1.7]	0.3 [0.0, 1.2]	0.3 [0.0, 1.3]	0.3 [0.0, 1.2]	1.0 [0.0, 2.6]	0.4 [0.0, 1.5]	0.4 [0.0, 1.4]	0.6 [0.0, 1.8]	0.4 [0.0, 1.7]		1.6 [0.1, 3.7]
W	PDA	**8.2 [4.7, 11.9]**	1.4 [0.0, 4.5]	0.5 [0.0, 1.4]	**4.3 [1.7, 7.8]**	0.2 [0.0, 0.8]	1.0 [0.0, 2.3]	0.1 [0.0, 0.7]	**4.3 [2.3, 6.6]**	**2.5 [0.5, 4.7]**	**1.6 [0.5, 3.0]**	**1.2 [0.2, 2.4]**	**4.0 [1.7, 6.6]**	**3.8 [2.0, 5.7]**	
	Stratified	**9.3 [5.3, 13.1]**	**3.9 [0.5, 6.7]**	0.7 [0.0, 1.7]	**6.3 [2.3, 10.2]**	0.5 [0.0, 1.3]	1.5 [0.0, 3.3]	0.2 [0.0, 0.9]	**4.8 [2.6, 7.2]**	**2.9 [0.8, 5.5]**	**2.3 [1.0, 4.1]**	**1.6 [0.4, 3.0]**	**5.0 [2.7, 7.8]**	**4.3 [2.2, 6.7]**	

Table L. Geographic region transition rate matrix from combined neuraminidase subtype model. Median rates and 95% highest posterior density intervals are displayed for both subsampling strategies. Rates colored in blue are statistically supported (Bayes factor > 100). (AK – Alaska, ALB – Alberta, BCO – British Columbia, GUA – Guatemala, MW – Midwest, NBR – New Brunswick, NE – Northeast, NFL – Newfoundland and Labrador, RP – Northern Rockies and Plains, NSC – Nova Scotia, NW – Northwest, OV – Ohio Valley, PEI – Prince Edward Island, QUE – Quebec, S – South, SE – Southeast, SON – Sonora, SW – Southwest, W – West)

		Sink													
Source	Sample	AK	ALB	GUA	MW	NBR	NE	NSC	NW	OV	RP	S	SE	SW	W
AK	PDA		**1.4 [0.5, 2.5]**	0.2 [0.0, 0.9]	1.3 [0.2, 2.6]	0.1 [0.0, 0.5]	0.3 [0.0, 1.0]	0.1 [0.0, 0.4]	**0.7 [0.1, 1.5]**	0.6 [0.0, 1.6]	0.3 [0.0, 1.0]	0.5 [0.0, 1.3]	0.2 [0.0, 0.7]	0.1 [0.0, 0.5]	**3.6 [1.7, 5.8]**
	Stratified		**1.6 [0.5, 3.0]**	0.2 [0.0, 0.6]	1.3 [0.1, 2.7]	0.1 [0.0, 0.4]	0.2 [0.0, 0.8]	0.1 [0.0, 0.5]	**1.4 [0.5, 2.5]**	0.8 [0.0, 2.0]	0.2 [0.0, 0.8]	0.4 [0.0, 1.1]	0.2 [0.0, 0.5]	0.1 [0.0, 0.4]	**3.3 [1.5, 5.4]**
ALB	PDA	0.7 [0.0, 2.5]		0.3 [0.0, 1.0]	0.8 [0.0, 2.9]	0.2 [0.0, 1.0]	0.4 [0.0, 1.4]	0.2 [0.0, 0.9]	0.9 [0.0, 2.6]	0.7 [0.0, 2.4]	1.1 [0.0, 2.8]	0.6 [0.0, 1.9]	0.3 [0.0, 1.3]	0.5 [0.0, 1.6]	0.9 [0.0, 3.2]
	Stratified	1.3 [0.0, 3.2]		0.2 [0.0, 1.1]	1.3 [0.0, 3.6]	0.2 [0.0, 0.8]	0.4 [0.0, 1.6]	0.2 [0.0, 0.9]	0.6 [0.0, 2.0]	1.6 [0.1, 3.9]	1.0 [0.1, 2.5]	0.6 [0.0, 1.9]	0.2 [0.0, 1.0]	0.4 [0.0, 1.4]	2.7 [0.4, 5.7]
GUA	PDA	0.7 [0.0, 2.0]	0.3 [0.0, 1.3]		0.5 [0.0, 1.8]	0.3 [0.0, 1.0]	0.3 [0.0, 1.4]	0.3 [0.0, 1.0]	0.2 [0.0, 1.1]	1.0 [0.0, 2.7]	0.3 [0.0, 1.1]	0.7 [0.0, 2.4]	0.2 [0.0, 1.1]	0.5 [0.0, 1.7]	0.5 [0.0, 2.0]
	Stratified	0.6 [0.0, 1.9]	0.3 [0.0, 1.3]		0.4 [0.0, 1.4]	0.3 [0.0, 1.1]	0.3 [0.0, 1.4]	0.2 [0.0, 1.0]	0.3 [0.0, 1.0]	0.5 [0.0, 1.8]	0.3 [0.0, 1.2]	0.4 [0.0, 1.6]	0.2 [0.0, 1.1]	0.2 [0.0, 1.1]	0.4 [0.0, 1.8]
MW	PDA	**2.7 [1.3, 4.5]**	**1.6 [0.5, 3.1]**	1.5 [0.3, 2.9]		**2.3 [1.1, 3.6]**	**7.6 [5.0, 10.6]**	**0.9 [0.3, 1.6]**	0.5 [0.0, 1.3]	**21.3 [16.7, 26.2]**	**3.6 [2.1, 5.2]**	**21.0 [16.8, 25.6]**	0.5 [0.0, 1.2]	0.1 [0.0, 0.7]	**3.3 [1.2, 5.5]**
	Stratified	**2.3 [0.9, 4.1]**	**1.9 [0.4, 3.8]**	1.0 [0.1, 2.1]		**2.5 [1.2, 4.0]**	**7.2 [4.4, 10.3]**	**0.9 [0.2, 1.8]**	0.7 [0.0, 1.6]	**20.9 [16.4, 26.0]**	**2.4 [1.1, 3.9]**	**16.6 [12.5, 21.0]**	0.6 [0.1, 1.3]	0.2 [0.0, 0.7]	**2.9 [0.9, 5.2]**
NBR	PDA	0.4 [0.0, 1.5]	0.3 [0.0, 1.2]	0.2 [0.0, 1.0]	0.4 [0.0, 1.8]		**2.3 [0.6, 4.5]**	**2.3 [0.8, 4.3]**	0.1 [0.0, 0.8]	0.4 [0.0, 1.3]	0.5 [0.0, 1.5]	0.4 [0.0, 1.5]	0.5 [0.0, 1.5]	0.2 [0.0, 1.0]	0.3 [0.0, 1.2]
	Stratified	0.2 [0.0, 1.0]	0.3 [0.0, 1.0]	0.3 [0.0, 1.0]	0.4 [0.0, 1.5]		1.2 [0.1, 2.9]	**5.1 [2.5, 8.2]**	0.2 [0.0, 0.8]	0.5 [0.0, 1.7]	0.4 [0.0, 1.3]	0.3 [0.0, 1.2]	0.6 [0.0, 1.6]	0.2 [0.0, 1.0]	0.3 [0.0, 1.3]
NE	PDA	0.9 [0.0, 2.1]	0.3 [0.0, 0.8]	0.2 [0.0, 0.8]	**4.4 [1.9, 7.3]**	**2.2 [0.7, 4.1]**		0.5 [0.0, 1.4]	0.2 [0.0, 0.7]	**2.5 [0.7, 4.7]**	0.2 [0.0, 0.9]	0.3 [0.0, 1.3]	0.4 [0.0, 1.1]	0.2 [0.0, 0.8]	0.6 [0.0, 1.8]
	Stratified	**1.1 [0.1, 2.3]**	0.4 [0.0, 1.2]	0.2 [0.0, 0.8]	**3.4 [1.3, 6.0]**	**2.7 [1.1, 4.6]**		0.5 [0.0, 1.4]	0.2 [0.0, 1.0]	1.6 [0.1, 3.4]	0.2 [0.0, 0.9]	0.4 [0.0, 1.1]	0.4 [0.0, 1.3]	0.2 [0.0, 0.6]	0.3 [0.0, 1.4]
NSC	PDA	0.3 [0.0, 1.1]	0.3 [0.0, 1.1]	0.2 [0.0, 1.0]	0.3 [0.0, 1.0]	0.3 [0.0, 1.2]	0.2 [0.0, 1.2]		0.3 [0.0, 1.1]	0.3 [0.0, 1.0]	0.3 [0.0, 1.4]	0.3 [0.0, 1.1]	0.3 [0.0, 1.5]	0.2 [0.0, 1.0]	0.3 [0.0, 1.0]
	Stratified	0.3 [0.0, 1.3]	0.2 [0.0, 1.1]	0.2 [0.0, 1.0]	0.3 [0.0, 1.2]	1.0 [0.0, 2.6]	0.4 [0.0, 1.6]		0.2 [0.0, 0.9]	0.3 [0.0, 1.1]	0.3 [0.0, 1.3]	0.2 [0.0, 1.2]	0.3 [0.0, 1.2]	0.2 [0.0, 1.0]	0.3 [0.0, 1.4]
NW	PDA	0.5 [0.0, 1.6]	0.6 [0.0, 1.7]	0.4 [0.0, 1.5]	0.3 [0.0, 1.4]	0.3 [0.0, 1.0]	0.3 [0.0, 1.4]	0.3 [0.0, 1.2]		0.3 [0.0, 1.4]	0.3 [0.0, 1.1]	0.4 [0.0, 1.7]	0.3 [0.0, 1.0]	0.5 [0.0, 1.7]	0.6 [0.0, 2.0]
	Stratified	1.0 [0.0, 2.7]	0.4 [0.0, 1.3]	0.4 [0.0, 1.4]	0.3 [0.0, 1.4]	0.2 [0.0, 1.0]	0.3 [0.0, 1.2]	0.2 [0.0, 1.1]		0.3 [0.0, 1.3]	0.3 [0.0, 1.0]	0.7 [0.0, 2.1]	0.3 [0.0, 1.1]	**1.1 [0.1, 2.6]**	0.7 [0.0, 2.2]
OV	PDA	0.7 [0.0, 1.8]	**1.4 [0.3, 2.8]**	0.5 [0.0, 1.3]	**8.7 [5.1, 12.6]**	0.2 [0.0, 0.9]	**7.0 [4.2, 10.0]**	0.2 [0.0, 0.6]	0.9 [0.1, 2.0]		0.3 [0.0, 1.0]	0.2 [0.0, 1.0]	0.3 [0.0, 1.3]	0.3 [0.0, 1.0]	0.3 [0.0, 1.4]
	Stratified	0.6 [0.0, 1.6]	1.3 [0.2, 2.8]	0.5 [0.0, 1.5]	**8.4 [5.1, 12.4]**	0.3 [0.0, 1.1]	**8.9 [5.8, 12.4]**	0.2 [0.0, 0.7]	0.6 [0.0, 1.6]		0.4 [0.0, 1.5]	0.3 [0.0, 1.2]	0.3 [0.0, 1.3]	0.3 [0.0, 1.0]	0.7 [0.0, 2.3]
RP	PDA	0.6 [0.0, 2.0]	0.5 [0.0, 1.8]	0.3 [0.0, 1.1]	0.4 [0.0, 1.4]	0.2 [0.0, 1.0]	0.2 [0.0, 1.1]	0.3 [0.0, 1.4]	0.3 [0.0, 1.1]	0.3 [0.0, 1.1]		0.4 [0.0, 1.6]	0.2 [0.0, 1.0]	0.3 [0.0, 1.0]	0.3 [0.0, 1.4]
	Stratified	0.4 [0.0, 1.9]	0.4 [0.0, 1.5]	0.2 [0.0, 0.8]	0.3 [0.0, 1.4]	0.3 [0.0, 1.2]	0.3 [0.0, 1.2]	0.3 [0.0, 1.1]	0.2 [0.0, 1.2]	0.4 [0.0, 1.5]		0.4 [0.0, 1.5]	0.3 [0.0, 1.1]	0.3 [0.0, 1.1]	0.3 [0.0, 1.5]
S	PDA	0.9 [0.0, 2.4]	0.7 [0.0, 1.8]	**3.7 [1.4, 6.5]**	**3.7 [0.7, 7.4]**	0.6 [0.0, 1.6]	**2.5 [0.6, 4.8]**	0.4 [0.0, 1.2]	0.4 [0.0, 1.4]	0.2 [0.0, 1.0]	0.7 [0.0, 1.9]		0.4 [0.0, 1.1]	0.7 [0.0, 1.7]	**3.5 [0.9, 6.5]**
	Stratified	1.2 [0.0, 2.8]	0.7 [0.0, 2.0]	**4.0 [2.0, 6.3]**	2.5 [0.2, 5.6]	0.5 [0.0, 1.4]	**2.3 [0.5, 4.4]**	0.2 [0.0, 0.8]	0.8 [0.0, 2.2]	0.3 [0.0, 1.3]	0.8 [0.1, 2.2]		0.4 [0.0, 1.2]	0.9 [0.1, 2.2]	**4.6 [1.7, 8.0]**
SE	PDA	0.3 [0.0, 1.2]	0.2 [0.0, 1.0]	0.2 [0.0, 1.1]	0.3 [0.0, 1.0]	0.3 [0.0, 1.2]	0.3 [0.0, 1.1]	0.2 [0.0, 1.0]	0.3 [0.0, 1.2]	0.3 [0.0, 1.2]	0.3 [0.0, 1.1]	0.2 [0.0, 1.0]		0.3 [0.0, 0.9]	0.3 [0.0, 1.2]
	Stratified	0.3 [0.0, 1.2]	0.2 [0.0, 1.1]	0.3 [0.0, 1.1]	0.3 [0.0, 1.0]	0.3 [0.0, 1.2]	0.3 [0.0, 1.1]	0.3 [0.0, 1.2]	0.2 [0.0, 1.2]	0.3 [0.0, 1.3]	0.2 [0.0, 1.1]	0.2 [0.0, 1.2]		0.2 [0.0, 1.1]	0.3 [0.0, 1.5]
SW	PDA	0.5 [0.0, 1.9]	0.2 [0.0, 1.1]	0.3 [0.0, 1.1]	0.4 [0.0, 1.6]	0.3 [0.0, 1.1]	0.2 [0.0, 1.1]	0.3 [0.0, 0.9]	0.5 [0.0, 1.6]	0.2 [0.0, 0.9]	0.3 [0.0, 1.1]	0.5 [0.0, 1.7]	0.3 [0.0, 1.0]		1.0 [0.0, 2.6]
	Stratified	0.5 [0.0, 1.8]	0.3 [0.0, 1.2]	0.2 [0.0, 1.1]	0.5 [0.0, 1.8]	0.2 [0.0, 1.0]	0.2 [0.0, 1.0]	0.3 [0.0, 1.1]	0.5 [0.0, 1.7]	0.3 [0.0, 1.2]	0.3 [0.0, 1.3]	0.6 [0.0, 1.8]	0.2 [0.0, 1.1]		1.8 [0.1, 3.7]
W	PDA	**8.8 [6.2, 11.6]**	**5.1 [3.1, 7.3]**	0.8 [0.1, 1.6]	**9.5 [6.4, 13.0]**	0.1 [0.0, 0.6]	**2.5 [1.0, 4.2]**	0.1 [0.0, 0.4]	**4.6 [2.9, 6.5]**	**2.6 [1.3, 3.9]**	**1.7 [0.7, 3.0]**	**3.2 [1.5, 5.3]**	0.4 [0.0, 1.0]	**2.1 [1.0, 3.4]**	
	Stratified	**8.7 [6.0, 11.7]**	**4.4 [2.3, 6.8]**	0.4 [0.0, 1.2]	**8.1 [5.0, 11.4]**	0.2 [0.0, 0.6]	**2.6 [1.1, 4.4]**	0.1 [0.0, 0.6]	**4.4 [2.7, 6.4]**	**2.7 [1.3, 4.1]**	**1.6 [0.6, 2.8]**	**3.5 [1.7, 5.6]**	0.4 [0.0, 1.0]	**2.0 [0.8, 3.4]**	

Table M. Names and GenBank accession numbers of 303 newly sequenced AIV nucleotide sequences.

Virus St Jude Id	Collection Date	Virus Name	Host Common Name	Host Scientific Name	Sample Location	Latitude	Longitude	Sub Type	Year	PB2	PB1	PA	HA	NP	NA	MP	NS
622999	8/28/16	A/MALLARD/ALBERTA/310/2016	Mallard Duck	Anas platyrhynchos	Hines Creek	56.16094	-118.515139	H1N2	2016								
622996	8/28/16	A/AMERICAN BLACK DUCK/ALBERTA/307/2016	American Black Duck	Anas rubripes	Hines Creek	56.16094	-118.515139	H4N8	2016								
622973	8/28/16	A/AMERICAN BLACK DUCK/ALBERTA/284/2016	American Black Duck	Anas rubripes	Hines Creek	56.16094	-118.515139	H3N8	2016								
622968	8/28/16	A/ENVIRONMENT/ALBERTA/279/2016	Environment	Env	Hines Creek	56.16094	-118.515139	H4N6	2016								
622963	8/28/16	A/AMERICAN BLACK DUCK/ALBERTA/274/2016	American Black Duck	Anas rubripes	Hines Creek	56.16094	-118.515139	H1N2	2016								
622955	8/28/16	A/SPOT-BILLED DUCK/ALBERTA/266/2016	Spot-billed Duck	Anas poecilorhyncha	Hines Creek	56.16094	-118.515139	H1N2	2016								
622953	8/28/16	A/SPOT-BILLED DUCK/ALBERTA/264/2016	Spot-billed Duck	Anas poecilorhyncha	Hines Creek	56.16094	-118.515139	H1N1	2016								
622951	8/28/16	A/ENVIRONMENT/ALBERTA/262/2016	Environment	Env	Hines Creek	56.16094	-118.515139	H4N6	2016								
622949	8/28/16	A/MALLARD/ALBERTA/260/2016	Mallard Duck	Anas platyrhynchos	Hines Creek	56.16094	-118.515139	H1N1	2016								
622927	8/28/16	A/AMERICAN BLACK DUCK/ALBERTA/238/2016	American Black Duck	Anas rubripes	Hines Creek	56.16094	-118.515139	H1N1	2016								
622922	8/27/16	A/BLUE WINGED TEAL/ALBERTA/233/2016	Blue-winged Teal	Anas discors	Ducks Unlimited Lake	56.16094	-118.515139	H12N5	2016								
622913	8/27/16	A/MALLARD/ALBERTA/224/2016	Mallard Duck	Anas platyrhynchos	Little Burnt Lake	56.16094	-118.515139	H4N6	2016								
622884	8/12/16	A/MALLARD/ALBERTA/195/2016	Mallard Duck	Anas platyrhynchos	Cardinal Lake	56.16094	-118.515139	H1N1	2016								
622808	8/11/16	A/DUCK/ALBERTA/119/2016	Duck	Anas sp.	Hines Creek	56.16094	-118.515139	H4N6	2016								
622807	8/11/16	A/AMERICAN BLACK DUCK/ALBERTA/118/2016	American Black Duck	Anas rubripes	Hines Creek	56.16094	-118.515139	H10N7	2016								
622799	8/11/16	A/SPOT-BILLED DUCK/ALBERTA/110/2016	Spot-billed Duck	Anas poecilorhyncha	Hines Creek	56.16094	-118.515139	H4N6	2016								
622785	8/11/16	A/AMERICAN BLACK DUCK/ALBERTA/96/2016	American Black Duck	Anas rubripes	Hines Creek	56.16094	-118.515139	H3N6	2016								
622778	8/11/16	A/ENVIRONMENT/ALBERTA/89/2016	Environment	Env	Hines Creek	56.16094	-118.515139	H4N6	2016								
622757	8/10/16	A/MALLARD/ALBERTA/68/2016	Mallard Duck	Anas platyrhynchos	Hines Creek	56.16094	-118.515139	H4N8	2016								
622755	8/10/16	A/MALLARD/ALBERTA/66/2016	Mallard Duck	Anas platyrhynchos	Hines Creek	56.16094	-118.515139	H3N8	2016								
622746	8/10/16	A/SPOT-BILLED DUCK/ALBERTA/57/2016	Spot-billed Duck	Anas poecilorhyncha	Hines Creek	56.16094	-118.515139	H4N6	2016								
622739	8/10/16	A,P/MALLARD/ALBERTA/50/2016	Mallard Duck	Anas platyrhynchos	Hines Creek	56.16094	-118.515139	H4N8/NDV	2016								
622725	8/10/16	A/DUCK/ALBERTA/36/2016	Duck	Anas sp.	Hines Creek	56.16094	-118.515139	H4N8	2016								
622720	8/10/16	A/DUCK/ALBERTA/31/2016	Duck	Anas sp.	Hines Creek	56.16094	-118.515139	H4N6	2016								
619174	5/25/16	A/CANADA GOOSE/DE/601/2016	Canada goose	Branta canadensis	Villas Beach, Delaware Bay	39.0181	-74.9473	H5N1	2016								
619151	5/25/16	A/LAUGHING GULL/DE/578/2016	Laughing Gull	Leucophaeus atricilla	High's Beach, Delaware Bay	39.07928	-74.9085051	H10N5	2016								
619097	5/25/16	A,P/RUDDY TURNSTONE/DE/524/2016	Ruddy Turnstone	Arenaria interpres	Pierces Point, Cape May County	39.084	-74.905	H3N8/H10N5/NDV	2016								
619093	5/25/16	A/SANDERLING/DE/520/2016	Sanderling	Calidris alba	Pierces Point, Cape May County	39.084	-74.905	H10N5	2016								
619092	5/25/16	A/SANDERLING/DE/519/2016	Sanderling	Calidris alba	Pierces Point, Cape May County	39.084	-74.905	H10N5	2016								
619085	5/25/16	A/SANDERLING/DE/512/2016	Sanderling	Calidris alba	Pierces Point, Cape May County	39.084	-74.905	H10N5	2016								
619080	5/25/16	A/SANDERLING/DE/507/2016	Sanderling	Calidris alba	Pierces Point, Cape May County	39.084	-74.905	H10N5	2016								
619041	5/25/16	A/RUDDY TURNSTONE/DE/468/2016	Ruddy Turnstone	Arenaria interpres	Pierces Point, Cape May County	39.084	-74.905	H10N5	2016								
619032	5/25/16	A/RUDDY TURNSTONE/DE/459/2016	Ruddy Turnstone	Arenaria interpres	Pierces Point, Cape May County	39.084	-74.905	H10N5	2016								
619023	5/25/16	A/RUDDY TURNSTONE/DE/450/2016	Ruddy Turnstone	Arenaria interpres	Pierces Point, Cape May County	39.084	-74.905	H3N8	2016								
618999	5/24/16	A/SANDERLING/DE/426/2016	Sanderling	Calidris alba	Villas Beach, Delaware Bay	39.0181	-74.9473	H3N8	2016								
618998	5/24/16	A/RUDDY TURNSTONE/DE/425/2016	Ruddy Turnstone	Arenaria interpres	Villas Beach, Delaware Bay	39.0181	-74.9473	H3N8	2016								
618982	5/24/16	A/RUDDY TURNSTONE/DE/409/2016	Ruddy Turnstone	Arenaria interpres	Villas Beach, Delaware Bay	39.0181	-74.9473	H10N5	2016								
618978	5/24/16	A/RUDDY TURNSTONE/DE/405/2016	Ruddy Turnstone	Arenaria interpres	Villas Beach, Delaware Bay	39.0181	-74.9473	H10N5	2016								
618941	5/24/16	A,P/RUDDY TURNSTONE/DE/368/2016	Ruddy Turnstone	Arenaria interpres	Villas Beach, Delaware Bay	39.0181	-74.9473	H10N4/NDV	2016								
618932	5/24/16	A/RUDDY TURNSTONE/DE/359/2016	Ruddy Turnstone	Arenaria interpres	Villas Beach, Delaware Bay	39.0181	-74.9473	H10N5	2016								
618930	5/24/16	A/RUDDY TURNSTONE/DE/357/2016	Ruddy Turnstone	Arenaria interpres	Villas Beach, Delaware Bay	39.0181	-74.9473	H10N5	2016								
618929	5/24/16	A/RUDDY TURNSTONE/DE/356/2016	Ruddy Turnstone	Arenaria interpres	Villas Beach, Delaware Bay	39.0181	-74.9473	H10N5	2016								
618925	5/24/16	A/RUDDY TURNSTONE/DE/352/2016	Ruddy Turnstone	Arenaria interpres	Villas Beach, Delaware Bay	39.0181	-74.9473	H10N4	2016								
618915	5/24/16	A/RUDDY TURNSTONE/DE/342/2016	Ruddy Turnstone	Arenaria interpres	Villas Beach, Delaware Bay	39.0181	-74.9473	H10N5	2016								
618913	5/24/16	A/RUDDY TURNSTONE/DE/340/2016	Ruddy Turnstone	Arenaria interpres	Villas Beach, Delaware Bay	39.0181	-74.9473	H10N4	2016								
618910	5/24/16	A/RUDDY TURNSTONE/DE/337/2016	Ruddy Turnstone	Arenaria interpres	Villas Beach, Delaware Bay	39.0181	-74.9473	H10N9	2016								
618909	5/24/16	A/RUDDY TURNSTONE/DE/336/2016	Ruddy Turnstone	Arenaria interpres	Villas Beach, Delaware Bay	39.0181	-74.9473	H10N5	2016								
618908	5/24/16	A,P/RUDDY TURNSTONE/DE/335/2016	Ruddy Turnstone	Arenaria interpres	Villas Beach, Delaware Bay	39.0181	-74.9473	H10N5/NDV	2016								
618904	5/24/16	A/RUDDY TURNSTONE/DE/331/2016	Ruddy Turnstone	Arenaria interpres	Villas Beach, Delaware Bay	39.0181	-74.9473	H3N8	2016								
618900	5/24/16	A/SEMIPALMATED SANDPIPER/DE/327/2016	Semipalmated Sandpiper	Calidris pusilla	Villas Beach, Delaware Bay	39.0181	-74.9473	H10N5	2016								
618898	5/24/16	A/RUDDY TURNSTONE/DE/325/2016	Ruddy Turnstone	Arenaria interpres	Villas Beach, Delaware Bay	39.0181	-74.9473	H10N5	2016								
618890	5/24/16	A/RUDDY TURNSTONE/DE/317/2016	Ruddy Turnstone	Arenaria interpres	Villas Beach, Delaware Bay	39.0181	-74.9473	H5N1	2016								
618888	5/24/16	A/RUDDY TURNSTONE/DE/315/2016	Ruddy Turnstone	Arenaria interpres	Villas Beach, Delaware Bay	39.0181	-74.9473	H10N5	2016								
618883	5/24/16	A/RED KNOT/DE/310/2016	Red Knot	Calidris canutus	Villas Beach, Delaware Bay	39.0181	-74.9473	H10N4	2016								
618882	5/24/16	A/RUDDY TURNSTONE/DE/309/2016	Ruddy Turnstone	Arenaria interpres	Villas Beach, Delaware Bay	39.0181	-74.9473	H10N5	2016								
618876	5/24/16	A/RUDDY TURNSTONE/DE/303/2016	Ruddy Turnstone	Arenaria interpres	Villas Beach, Delaware Bay	39.0181	-74.9473	H11N2	2016								
618872	5/24/16	A/RUDDY TURNSTONE/DE/299/2016	Ruddy Turnstone	Arenaria interpres	Villas Beach, Delaware Bay	39.0181	-74.9473	H10N9	2016								
618865	5/24/16	A/RUDDY TURNSTONE/DE/292/2016	Ruddy Turnstone	Arenaria interpres	Villas Beach, Delaware Bay	39.0181	-74.9473	H11N2	2016								
618837	5/24/16	A/GULL/DE/264/2016	Gull	Larus sp.	Reeds Beach, Delaware Bay	39.0019	-74.014863	H10N5	2016								
618829	5/24/16	A/HERRING GULL/DE/256/2016	Herring Gull	Larus smithsonianus	Reeds Beach, Delaware Bay	39.0019	-74.014863	H10N5	2016								
618827	5/24/16	A/GULL/DE/254/2016	Gull	Larus sp.	Reeds Beach, Delaware Bay	39.0019	-74.014863	H10N5	2016								
618796	5/24/16	A/GULL/DE/223/2016	Gull	Larus sp.	Reeds Beach, Delaware Bay	39.0019	-74.014863	H10N5	2016								
618794	5/24/16	A/GULL/DE/221/2016	Gull	Larus sp.	Reeds Beach, Delaware Bay	39.0019	-74.014863	H10N4	2016								
618791	5/24/16	A/GULL/DE/218/2016	Gull	Larus sp.	Reeds Beach, Delaware Bay	39.0019	-74.014863	H6N5	2016								
618770	5/23/16	A/RED KNOT/DE/197/2016	Red Knot	Calidris canutus	Cooks Beach, Delaware Bay	39.00181	-74.014878	H10N5	2016								
618743	5/23/16	A/RUDDY TURNSTONE/DE/170/2016	Ruddy Turnstone	Arenaria interpres	Cooks Beach, Delaware Bay	39.00181	-74.014878	H10N5	2016								
618740	5/23/16	A,P/RUDDY TURNSTONE/DE/167/2016	Ruddy Turnstone	Arenaria interpres	Cooks Beach, Delaware Bay	39.00181	-74.014878	H10N5/NDV	2016								
618728	5/23/16	A/RED KNOT/DE/155/2016	Red Knot	Calidris canutus	Cooks Beach, Delaware Bay	39.00181	-74.014878	H10N4	2016								
618717	5/23/16	A/RUDDY TURNSTONE/DE/144/2016	Ruddy Turnstone	Arenaria interpres	Cooks Beach, Delaware Bay	39.00181	-74.014878	H3N8	2016								
618714	5/23/16	A/RUDDY TURNSTONE/DE/141/2016	Ruddy Turnstone	Arenaria interpres	Cooks Beach, Delaware Bay	39.00181	-74.014878	H10N4	2016								
618713	5/23/16	A/RUDDY TURNSTONE/DE/140/2016	Ruddy Turnstone	Arenaria interpres	Cooks Beach, Delaware Bay	39.00181	-74.014878	H10N4	2016								
618712	5/23/16	A/RUDDY TURNSTONE/DE/139/2016	Ruddy Turnstone	Arenaria interpres	Cooks Beach, Delaware Bay	39.00181	-74.014878	H6N8	2016								
618703	5/23/16	A/RUDDY TURNSTONE/DE/130/2016	Ruddy Turnstone	Arenaria interpres	Cooks Beach, Delaware Bay	39.00181	-74.014878	H10N5	2016								
618701	5/23/16	A/SEMIPALMATED SANDPIPER/DE/128/2016	Semipalmated Sandpiper	Calidris pusilla	Cooks Beach, Delaware Bay	39.00181	-74.014878	H10N5	2016								
559027	8/12/15	A/MALLARD/ALBERTA/612/2015	Mallard Duck	Anas platyrhynchos	Hay Lake	56.16094	-118.515139	H3N8	2015								
559021	8/12/15	A/MALLARD/ALBERTA/606/2015	Mallard Duck	Anas platyrhynchos	Hay Lake	56.16094	-118.515139	H1N1	2015								
559019	8/12/15	A/MALLARD/ALBERTA/604/2015	Mallard Duck	Anas platyrhynchos	Hay Lake	56.16094	-118.515139	H1N8	2015								
559018	8/12/15	A/MALLARD/ALBERTA/603/2015	Mallard Duck	Anas platyrhynchos	Hay Lake	56.16094	-118.515139	H1N1	2015								
558940	8/11/15	A,P/MALLARD/ALBERTA/525/2015	Mallard Duck	Anas platyrhynchos	Cardinal Lake	56.25871	-117.805228	H3N8/NDV	2015								
558930	8/11/15	A/MALLARD/ALBERTA/515/2015	Mallard Duck	Anas platyrhynchos	Cardinal Lake	56.25871	-117.805228	H3N8	2015								
558892	8/11/15	A/MALLARD/ALBERTA/477/2015	Mallard Duck	Anas platyrhynchos	Hay Lake	56.16094	-118.515139	H10N7	2015								
558879	8/11/15	A/MALLARD/ALBERTA/464/2015	Mallard Duck	Anas platyrhynchos	Hay Lake	56.16094	-118.515139	H10N7	2015								
558870	8/11/15	A/MALLARD/ALBERTA/455/2015	Mallard Duck	Anas platyrhynchos	Hay Lake	56.16094	-118.515139	H4N6	2015								
558862	8/11/15	A,P/MALLARD/ALBERTA/447/2015	Mallard Duck	Anas platyrhynchos	Hay Lake	56.16094	-118.515139	H3N8/NDV	2015								
558794	8/11/15	A,P/MALLARD/ALBERTA/379/2015	Mallard Duck	Anas platyrhynchos	Hay Lake	56.16094	-118.515139	H11N9/NDV	2015								
558780	8/11/15	A/MALLARD/ALBERTA/365/2015	Mallard Duck	Anas platyrhynchos	Hay Lake	56.16094	-118.515139	H3N8	2015								
558648	8/11/15	A/MALLARD/ALBERTA/233/2015	Mallard Duck	Anas platyrhynchos	Hay Lake	56.16094	-118.515139	H1N1	2015								
558618	8/11/15	A/MALLARD/ALBERTA/203/2015	Mallard Duck	Anas platyrhynchos	Hay Lake	56.16094	-118.515139	H8N4	2015								
558546	8/11/15	A/MALLARD/ALBERTA/131/2015	Mallard Duck	Anas platyrhynchos	Hay Lake	56.16094	-118.515139	H3N8	2015								
558482	8/11/15	A/MALLARD/ALBERTA/67/2015	Mallard Duck	Anas platyrhynchos	Hay Lake	56.16094	-118.515139	H3N8	2015								
558430	8/11/15	A/MALLARD/ALBERTA/15/2015	Mallard Duck	Anas platyrhynchos	Hay Lake	56.16094	-118.515139	H3N8	2015								
551521	5/21/15	A/RUDDY TURNSTONE/DE/531/2015	Ruddy Turnstone	Arenaria interpres	High's Beach, Delaware Bay	39.07928	-74.9085051	H7N3	2015	MK637212	MK637214	MK637210	MK637213	MK637216	MK637209	MK637211	MK637215
551513	5/21/15	A/SANDERLING/DE/523/2015	Sanderling	Calidris alba	High's Beach, Delaware Bay	39.07928	-74.9085051	H1N1	2015	MK637162	MK637164	MK637163	MK637168	MK637166	MK637161	MK637167	MK637165
551335	5/19/15	A/RUDDY TURNSTONE/DE/345/2015	Ruddy Turnstone	Arenaria interpres	Pierces Point, Cape May County	39.084	-74.905	H1N1	2015	MK724045	MK637150	MK696136	MK637147	MK637149	MK637152	MK637148	MK637151
551318	5/19/15	A/RUDDY TURNSTONE/DE/328/2015	Ruddy Turnstone	Arenaria interpres	Pierces Point, Cape May County	39.084	-74.905	H1N1	2015	MK637130	MK637127	MK637128	MK637129	MK637125	MK637123	MK637124	MK637126
551313	5/19/15	A/SEMIPALMATED SANDPIPER/DE/323/2015	Semipalmated Sandpiper	Calidris pusilla	Pierces Point, Cape May County	39.084	-74.905	H11N2	2015	MK637234	MK637233	MK637237	MK637236	MK637238	MK637235	MK637239	MK637240
551297	5/19/15	A/SANDERLING/DE/307/2015	Sanderling	Calidris alba	Pierces Point, Cape May County	39.084	-74.905	H1N1	2015	MK637276	MK637273	MK637279	MK637274	MK637278	MK637272	MK637275	MK637277
551272	5/19/15	A/RUDDY TURNSTONE/DE/282/2015	Ruddy Turnstone	Arenaria interpres	Cooks Beach, Delaware Bay	39.00181	-74.014878	H7N1	2015	MK637225	MK637229	MK637232	MK637227	MK637226	MK637231	MK637228	MK637230
551268	5/19/15	A/DUNLIN/DE/278/2015	Dunlin	Calidris alpina	Cooks Beach, Delaware Bay	39.00181	-74.014878	H7N3	2015	MK724050	MK637330	MK637329	MK637333	MK637331	MK637327	MK637328	MK637332
551263	5/19/15	A/SEMIPALMATED SANDPIPER/DE/273/2015	Semipalmated Sandpiper	Calidris pusilla	Cooks Beach, Delaware Bay	39.00181	-74.014878	H7N3	2015	MK724049	MK637111	MK637110	MK637113	MK637109	MK637112	MK637108	MK637114
551262	5/19/15	A/RUDDY TURNSTONE/DE/272/2015	Ruddy Turnstone	Arenaria interpres	Cooks Beach, Delaware Bay	39.00181	-74.014878	H7N3	2015	MK637302	MK637298	MK724061	MK637301	MK637296	MK637299	MK637300	MK637297
551251	5/19/15	A/RUDDY TURNSTONE/DE/261/2015	Ruddy Turnstone	Arenaria interpres	Cooks Beach, Delaware Bay	39.00181	-74.014878	H7N3	2015	MK637286	MK637287	MK637285	MK637283	MK637280	MK637284	MK637281	MK637282
551247	5/19/15	A/RUDDY TURNSTONE/DE/257/2015	Ruddy Turnstone	Arenaria interpres	Cooks Beach, Delaware Bay	39.00181	-74.014878	H7N3	2015	MK637338	MK637335	MK637337	MK637340	MK637336	MK637339	MK637341	MK637334
551243	5/19/15	A/RED KNOT/DE/253/2015	Red Knot	Calidris canutus	Cooks Beach, Delaware Bay	39.00181	-74.014878	H7N3	2015	MK637132	MK637131	MK637136	MK637135	MK637133	MK637138	MK637134	MK637137
551215	5/19/15	A/SANDERLING/DE/225/2015	Sanderling	Calidris alba	Cooks Beach, Delaware Bay	39.00181	-74.014878	H7N3	2015	MK637192	MK637186	MK637185	MK637187	MK637188	MK637191	MK637189	MK637190
551051	5/18/15	A/RUDDY TURNSTONE/DE/61/2015	Ruddy Turnstone	Arenaria interpres	East Point Beach, Delaware Bay	39.21825	-74.96042	H1N1	2015								
551005	5/18/15	A/RUDDY TURNSTONE/DE/15/2015	Ruddy Turnstone	Arenaria interpres	East Point Beach, Delaware Bay	39.21825	-74.96042	H7N3/NDV	2015								
513027	5/21/14	A/RUDDY TURNSTONE/DE/600/2014	Ruddy Turnstone	Arenaria interpres	High's Beach, Delaware Bay	39.003848	-75.002814	H12N4	2014	MK637263	MK637259	MK637258	MK637260	MK637256	MK637261	MK637257	MK637262
513009	5/21/14	A/RUDDY TURNSTONE/DE/582/2014	Ruddy Turnstone	Arenaria interpres	High's Beach, Delaware Bay	39.003848	-75.002814	H12N4	2014	MK637170	MK637175	MK637173	MK637172	MK637176	MK637171	MK637169	MK637174
512978	5/21/14	A/RUDDY TURNSTONE/DE/551/2014	Ruddy Turnstone	Arenaria interpres	High's Beach, Delaware Bay	39.003848	-75.002814	H12N4	2014	MK637346	MK637348	MK637344	MK724042	MK637345	MK637347	MK637343	MK637342
512970	5/21/14	A/RUDDY TURNSTONE/DE/543/2014	Ruddy Turnstone	Arenaria interpres	High's Beach, Delaware Bay	39.003848	-75.002814	H11N2	2014	MK637180	MK637178	MK637179	MK637184	MK637182	MK637181	MK637183	MK637177
512965	5/21/14	A/RUDDY TURNSTONE/DE/538/2014	Ruddy Turnstone	Arenaria interpres	High's Beach, Delaware Bay	39.003848	-75.002814	H11N2	2014	MK637270	MK637264	MK637265	MK637271	MK637269	MK637266	MK637268	MK637267
512964	5/21/14	A/RUDDY TURNSTONE/DE/537/2014	Ruddy Turnstone	Arenaria interpres	High's Beach, Delaware Bay	39.003848	-75.002814	H12N4	2014	MK637254	MK637251	MK637252	MK724063	MK637249	MK637253	MK637255	MK637250
512959	5/21/14	A/RUDDY TURNSTONE/DE/532/2014	Ruddy Turnstone	Arenaria interpres	High's Beach, Delaware Bay	39.003848	-75.002814	H12N4	2014	MK637325	MK637326	MK637322	MK637324	MK637323	MK637320	MK637319	MK637321
512952	5/21/14	A/RUDDY TURNSTONE/DE/525/2014	Ruddy Turnstone	Arenaria interpres	High's Beach, Delaware Bay	39.003848	-75.002814	H12N4	2014	MK637305	MK637303	MK637309	MK637304	MK637306	MK637310	MK637308	MK637307
512946	5/21/14	A/LAUGHING GULL/DE/519/2014	Laughing Gull	Leucophaeus atricilla	High's Beach, Delaware Bay	39.003848	-75.002814	H6N2	2014								
512943	5/21/14	A/RUDDY TURNSTONE/DE/516/2014	Ruddy Turnstone	Arenaria interpres	High's Beach, Delaware Bay	39.003848	-75.002814	H12N4	2014	MK637315	MK637312	MK637311	MK637314	MK637317	MK637316	MK637313	MK637318
512913	5/21/14	A/RUDDY TURNSTONE/DE/486/2014	Ruddy Turnstone	Arenaria interpres	East Point Beach, Delaware Bay	39.21825	-74.96042	H16N6	2014	MK637244	MK637246	MK637242	MK637241	MK637243	MK637248	MK637247	MK637245
512864	5/21/14	A/RUDDY TURNSTONE/DE/437/2014	Ruddy Turnstone	Arenaria interpres	East Point Beach, Delaware Bay	39.21825	-74.96042	H12N4	2014	MK637359	MK637364	MK637362	MK637358	MK637357	MK637363	MK637361	MK637360
512786	5/20/14	A/LAUGHING GULL/DE/359/2014	Laughing Gull	Leucophaeus atricilla	Moores Beach, Delaware Bay	39.1875	-74.95056	H6N4	2014	MK637386	MK637383	MK637384	MK637381	MK637385	MK637382	MK637388	MK637387
512785	5/20/14	A/LAUGHING GULL/DE/358/2014	Laughing Gull	Leucophaeus atricilla	Moores Beach, Delaware Bay	39.1875	-74.95056	H13N6	2014	MK637389	MK637395	MK637394	MK637396	MK637390	MK637391	MK637392	MK637393
512778	5/20/14	A/LAUGHING GULL/DE/351/2014	Laughing Gull	Leucophaeus atricilla	Moores Beach, Delaware Bay	39.1875	-74.95056	H13N6	2014	MK637366	MK637372	MK637367	MK637365	MK637370	MK637368	MK637369	MK637371
512777	5/20/14	A/LAUGHING GULL/DE/350/2014	Laughing Gull	Leucophaeus atricilla	Moores Beach, Delaware Bay	39.1875	-74.95056	H13N6	2014	MK637035	MK637031	MK637037	MK637038	MK637036	MK637034	MK637033	MK637032
512767	5/20/14	A/LAUGHING GULL/DE/340/2014	Laughing Gull	Leucophaeus atricilla	Moores Beach, Delaware Bay	39.1875	-74.95056	H13N6	2014	MK637349	MK637354	MK637350	MK637356	MK637355	MK637351	MK637352	MK637353
512755	5/20/14	A/LAUGHING GULL/DE/328/2014	Laughing Gull	Leucophaeus atricilla	Moores Beach, Delaware Bay	39.1875	-74.95056	H13N6	2014	MK637375	MK637374	MK637377	MK637376	MK637378	MK637379	MK637373	MK637380
512754	5/20/14	A/LAUGHING GULL/DE/327/2014	Laughing Gull	Leucophaeus atricilla	Moores Beach, Delaware Bay	39.1875	-74.95056	H13N6	2014	MK637289	MK637295	MK637294	MK637291	MK637290	MK637293	MK637292	MK637288
512686	5/20/14	A/RUDDY TURNSTONE/DE/259/2014	Ruddy Turnstone	Arenaria interpres	Pierces Point, Cape May County	39.084	-74.905	H12N4	2014	MK637403	MK637400	MK637404	MK637397	MK637401	MK637402	MK637399	MK637398
512662	5/20/14	A/RUDDY TURNSTONE/DE/235/2014	Ruddy Turnstone	Arenaria interpres	Pierces Point, Cape May County	39.084	-74.905	H12N4	2014	MK637208	MK637204	MK637202	MK637207	MK637201	MK637205	MK637206	MK637203
512629	5/20/14	A/SEMI-PALMATED SANDPIPER/DE/202/2014	Semipalmated Sandpiper	Calidris pusilla	Pierces Point, Cape May County	39.084	-74.905	H12N4	2014	MK637095	MK637092	MK637098	MK637096	MK637097	MK637093	MK637094	MK637099
512622	5/20/14	A/RUDDY TURNSTONE/DE/195/2014	Ruddy Turnstone	Arenaria interpres	Pierces Point, Cape May County	39.084	-74.905	H12N4	2014	MK637199	MK637200	MK637198	MK637195	MK637193	MK637196	MK637194	MK637197
512590	5/20/14	A/RUDDY TURNSTONE/DE/163/2014	Ruddy Turnstone	Arenaria interpres	Pierces Point, Cape May County	39.084	-74.905	H12N4	2014	MK637089	MK637085	MK637084	MK637090	MK637088	MK637086	MK637087	MK637091
512578	5/20/14	A/RUDDY TURNSTONE/DE/151/2014	Ruddy Turnstone	Arenaria interpres	Pierces Point, Cape May County	39.084	-74.905	H12N4	2014	MK637156	MK637158	MK637157	MK637159	MK637154	MK637153	MK637160	MK637155
512574	5/20/14	A/RUDDY TURNSTONE/DE/147/2014	Ruddy Turnstone	Arenaria interpres	Pierces Point, Cape May County	39.084	-74.905	H12N4	2014	MK637142	MK637141	MK637140	MK637145	MK637144	MK637143	MK637146	MK637139
512475	5/19/14	A/RUDDY TURNSTONE/DE/48/2014	Ruddy Turnstone	Arenaria interpres	Moores Beach, Delaware Bay	39.1875	-74.95056	H13N6	2014	MK637102	MK637103	MK637101	MK637106	MK637107	MK637104	MK637105	MK637100
512466	5/19/14	A/LAUGHING GULL/DE/39/2014	Laughing Gull	Leucophaeus atricilla	Moores Beach, Delaware Bay	39.1875	-74.95056	H13N6	2014	MK637115	MK637121	MK637120	MK637116	MK637117	MK637118	MK637122	MK637119
512457	5/19/14	A/LAUGHING GULL/DE/30/2014	Laughing Gull	Leucophaeus atricilla	Moores Beach, Delaware Bay	39.1875	-74.95056	H13N6	2014	MK637218	MK637222	MK637219	MK637221	MK637220	MK637224	MK637223	MK637217
512454	5/19/14	A/HERRING GULL/DE/27/2014	Herring Gull	Larus smithsonianus	Moores Beach, Delaware Bay	39.1875	-74.95056	H6N2	2014								
512449	5/19/14	A/HERRING GULL/DE/22/2014	Herring Gull	Larus smithsonianus	Moores Beach, Delaware Bay	39.1875	-74.95056	H13N6	2014	MK637082	MK637076	MK637079	MK637080	MK637081	MK637078	MK637083	MK637077
359528	5/23/13	A/RUDDY TURNSTONE/DE/552/2013	Ruddy Turnstone	Arenaria interpres	High's Beach, Delaware Bay	39.023	-74.931	H11N2	2013	MK628001	MK628002	MK628004	MK628005	MK628007	MK628006	MK628008	MK628003
359515	5/23/13	A/RUDDY TURNSTONE/DE/539/2013	Ruddy Turnstone	Arenaria interpres	High's Beach, Delaware Bay	39.023	-74.931	H6N8	2013	MK627994	MK627993	MK627997	MK627999	MK627998	MK628000	MK627996	MK627995
359514	5/23/13	A/RUDDY TURNSTONE/DE/538/2013	Ruddy Turnstone	Arenaria interpres	High's Beach, Delaware Bay	39.023	-74.931	H10N7	2013	MK628017	MK628022	MK628019	MK628024	MK628018	MK628021	MK628020	MK628023
359507	5/23/13	A/RUDDY TURNSTONE/DE/531/2013	Ruddy Turnstone	Arenaria interpres	High's Beach, Delaware Bay	39.023	-74.931	H10N7	2013	MK627988	MK627989	MK627990	MK627992	MK627986	MK627987	MK627985	MK627991
359495	5/23/13	A/RUDDY TURNSTONE/DE/519/2013	Ruddy Turnstone	Arenaria interpres	High's Beach, Delaware Bay	39.023	-74.931	H10N8	2013	MK628016	MK628013	MK628010	MK628014	MK628012	MK628011	MK628015	MK628009
359478	5/23/13	A/RUDDY TURNSTONE/DE/502/2013	Ruddy Turnstone	Arenaria interpres	High's Beach, Delaware Bay	39.023	-74.931	H11N2	2013	MK628034	MK628032	MK628033	MK628027	MK628030	MK628029	MK628028	MK628031
359477	5/23/13	A/GULL/DE/501/2013	Gull	Larus sp.	High's Beach, Delaware Bay	39.023	-74.931	H10N8	2013	MK628048	MK628050	MK628047	MK628049	MK628046	MK628045	MK628044	MK628043
359457	5/23/13	A/RUDDY TURNSTONE/DE/481/2013	Ruddy Turnstone	Arenaria interpres	High's Beach, Delaware Bay	39.023	-74.931	H11N2	2013	MK628060	MK628066	MK628064	MK628065	MK628062	MK628063	MK628061	MK628059
359455	5/23/13	A/RUDDY TURNSTONE/DE/479/2013	Ruddy Turnstone	Arenaria interpres	High's Beach, Delaware Bay	39.023	-74.931	H10N7	2013	MK628042	MK628039	MK628037	MK628040	MK628035	MK628041	MK628038	MK628036
359450	5/23/13	A/RUDDY TURNSTONE/DE/474/2013	Ruddy Turnstone	Arenaria interpres	High's Beach, Delaware Bay	39.023	-74.931	H10N1	2013	MK628053	MK628052	MK628058	MK628057	MK628056	MK628055	MK628051	MK628054
359444	5/23/13	A/RUDDY TURNSTONE/DE/468/2013	Ruddy Turnstone	Arenaria interpres	High's Beach, Delaware Bay	39.023	-74.931	H10N9	2013	MK628074	MK628076	MK628078	MK628079	MK628077	MK628072	MK628075	MK628073
359427	5/23/13	A/RUDDY TURNSTONE/DE/451/2013	Ruddy Turnstone	Arenaria interpres	High's Beach, Delaware Bay	39.023	-74.931	H10N7	2013	MK628084	MK628083	MK628081	MK628087	MK628082	MK628086	MK628080	MK628085
359409	5/23/13	A/RED KNOT/DE/433/2013	Red Knot	Calidris canutus	High's Beach, Delaware Bay	39.023	-74.931	H10N2	2013	MK726296	MK726294	MK726295	MK628067	MK628070	MK628068	MK628069	MK628071
359407	5/23/13	A/RED KNOT/DE/431/2013	Red Knot	Calidris canutus	High's Beach, Delaware Bay	39.023	-74.931	H11N7	2013	MK628090	MK628092	MK628093	MK628095	MK628089	MK628094	MK628088	MK628091
359400	5/23/13	A/RED KNOT/DE/424/2013	Red Knot	Calidris canutus	High's Beach, Delaware Bay	39.023	-74.931	H11N7	2013	MK628109	MK628110	MK628103	MK628107	MK628108	MK628106	MK628104	MK628105
359396	5/23/13	A/RED KNOT/DE/420/2013	Red Knot	Calidris canutus	High's Beach, Delaware Bay	39.023	-74.931	H10N7	2013	MK628120	MK628122	MK628125	MK628126	MK628121	MK628123	MK628124	MK628119
359392	5/23/13	A/RUDDY TURNSTONE/DE/416/2013	Ruddy Turnstone	Arenaria interpres	High's Beach, Delaware Bay	39.023	-74.931	H10N1	2013	MK628100	MK696421	MK628098	MK628096	MK628099	MK628101	MK628097	MK628102
359388	5/23/13	A/RUDDY TURNSTONE/DE/412/2013	Ruddy Turnstone	Arenaria interpres	High's Beach, Delaware Bay	39.023	-74.931	H10N8	2013	MK628129	MK628128	MK628130	MK628127	MK628132	MK628133	MK628131	MK628134
